# GINClus: RNA structural motif clustering using graph isomorphism network

**DOI:** 10.1093/nargab/lqaf050

**Published:** 2025-04-26

**Authors:** Nabila Shahnaz Khan, Md Mahfuzur Rahaman, Shaojie Zhang

**Affiliations:** Department of Computer Science, University of Central Florida, Orlando, FL 32816, United States; Department of Computer Science, University of Central Florida, Orlando, FL 32816, United States; Department of Computer Science, University of Central Florida, Orlando, FL 32816, United States

## Abstract

Ribonucleic acid (RNA) structural motif identification is a crucial step for understanding RNA structure and functionality. Due to the complexity and variations of RNA 3D structures, identifying RNA structural motifs is challenging and time-consuming. Particularly, discovering new RNA structural motif families is a hard problem and still largely depends on manual analysis. In this paper, we proposed an RNA structural motif clustering tool, named GINClus, which uses a semi-supervised deep learning model to cluster RNA motif candidates (RNA loop regions) based on both base interaction and 3D structure similarities. GINClus converts base interactions and 3D structures of RNA motif candidates into graph representations and using graph isomorphism network (GIN) model in combination with *K*-means and hierarchical agglomerative clustering, GINClus clusters the RNA motif candidates based on their structural similarities. GINClus has a clustering accuracy of 87.88% for known internal loop motifs and 97.69% for known hairpin loop motifs. Using GINClus, we successfully clustered the motifs of the same families together and were able to find 927 new instances of Sarcin-ricin, Kink-turn, Tandem-shear, Hook-turn, E-loop, C-loop, T-loop, and GNRA loop motif families. We also identified 12 new RNA structural motif families with unique structure and base-pair interactions.

## Introduction

Ribonucleic acid (RNA) is a single-stranded macromolecule that is responsible for essential cellular functions such as transcription, gene regulation, protein synthesis, and RNA interference. RNA folds into specific three-dimensional conserved regions to participate in different cellular functionalities. The conserved regions occur recurrently in nonhomologous RNA chains, and contain common base-pair interactions, base-stacking interactions, and structure conformation. These recurrent three-dimensional structures are known as RNA structural motifs. RNA structural motifs are structurally very stable and are considered the building blocks of RNA structures [1, 2]. According to research, RNA loop regions can act as binding sites for proteins or other molecules [3]. Many recurrent internal loops, hairpin loops, and junction loops embedded within regular helical regions form modular RNA structural motifs [4]. RNA structural motifs have been assigned to different well-known motif families based on their structural similarities [5].

Among different research works related to RNA structural motifs, motif search, classification, and clustering are most common. The existing works so far can be divided into two categories in general. The first category specifically focuses on the geometrical 3D structure comparison to calculate the backbone structure similarity. Apostolico *et al.* proposed a geometric shape histogram method to characterize RNA structural motifs at specific locations and later searched for other motifs by comparing the distance between multiple shape histograms [6]. This shape histogram approach along with backbone atom Root Mean Square Deviation (RMSD) was used by Sargsyan *et al.* [7] to analyze the distribution of RNA structural motifs along a given RNA chain. To search and identify RNA structural motifs, both PRIMOS [8] and COMPADRES [9] used the concept of “RNA worm” where they used sequentially ordered coordinates of pseudotorsion angles η and θ to define RNA conformation.

The second approach depends on the RNA molecular interactions, as base-pair interactions and base-stacking interactions are highly conserved in the recurrent regions of RNA. Djelloul *et al.* [10], Zhong *et al.* [11], and Ge *et al.* [5] used RNA interactions to identify and cluster RNA structural motifs. Both RNAMSC [11] and the extended version of RNAMSC [5] clustered RNA structural motifs using the base-pair interaction-based alignment tool RNAMotifScan. Djelloul *et al.* [10] also proposed an RNA structural motif clustering approach based on phosphodiester bonds and base-pair interactions. Here, they used graph representation for the RNA tertiary structures and hierarchically clustered secondary structural elements based on a similarity measure, which is calculated by generating the largest extensible common noncanonical subgraph (LENCS) using graph isomorphism algorithm. Apart from [10], both Wang *et al.* [12] and Harrison *et al.* [13] used graph representation to discover similar RNA motif substructures. To find similar structure patterns from RNA 3D database, Wang *et al.* [12] used a subgraph mining algorithm while NASSAM [13] used subgraph isomorphism algorithm. Oliver *et al.* [14] proposed VeRNAl that uses graph representation learning along with *K*-means clustering to identify recurrent fuzzy motifs with similar base pairing. As both backbone conformation and base interaction play important roles in RNA structure formation, focusing on either structure or interaction-based similarity will impose limitations on RNA motif identification. To overcome these limitations, the RNA 3D Motif Atlas introduced a combination of geometric structure and pairwise interaction information in classifying RNA structural motifs [4]. However, they perform all-against-all structural alignment using tool FR3D [15], which can be time-consuming.

Till now, different machine learning methods have been successfully utilized for RNA tertiary structure prediction, RNA–protein binding site prediction, and RNA sequence motif mining [16–18]. Among these methods, graph neural network (GNN) is frequently used in the field of bioinformatics and molecular biology, as GNN can capture the topological and structural information of molecules represented as graphs [19]. However, some variants of GNN fail to distinguish simple graph structures and isomorphic graphs [20]. The graph representation of similar RNA structural motifs can be isomorphic to each other due to their different structural orientations. Hence, for this work, we used graph isomorphism network (GIN), which is a powerful variation of GNN that can distinguish nonisomorphic graphs and has equal discriminative power as the Weisfeiler–Lehman graph isomorphism test [20].

In this paper, we proposed an RNA structural motif clustering tool, named GINClus, for clustering RNA motif candidates using GIN combined with *K*-means and hierarchical agglomerative clustering algorithms. Here, RNA loop regions are considered as RNA motif candidates. GINClus clusters the motif candidates based on both backbone conformation and base interaction similarity where base interaction refers to base-pair interactions and base-stacking interactions. GINClus uses the graph classification model of GIN, which maps graph representations of structural motif candidates in the embedding space as a feature set. Based on this feature set, GINClus first clusters the RNA motif candidates using *K*-means clustering, and then subclusters the motif candidates using hierarchical agglomerative clustering. GINClus has an overall clustering accuracy of 87.88% for known internal loop motifs and 97.69% for known hairpin loop motifs. Using GINClus, we generated 406 subclusters for 2656 internal loops and 413 subclusters for 2091 hairpin loops based on their structural similarities. After analyzing these subclusters, we found 81 new Sarcin-ricin (SR), 66 new Kink-turn (KT), 74 new Tandem-shear (TS), 47 new Hook-turn (HT), 92 new E-loop (EL), 102 new C-loop (CL), 85 new T-loop (TL), and 380 new GNRA tetraloop (GL) loop motifs and identified 12 new RNA structural motif families.

Most of the motif classification and clustering work done so far depends on comparative pairwise structural alignment and manual analysis which can be tedious and extremely time-consuming. GINClus is independent of all-against-all pairwise structural motif alignment and therefore is computationally faster in comparison to other existing tools. While most of the existing approaches either rely on motif backbone structure or molecular interactions, GINClus utilizes both of these specifications to distinguish motifs in a detailed and extensive manner. The self-trained, semi-supervised learning nature of GIN model further enables GINClus to accumulate the motifs of an unknown family based on their structural similarity. GINClus generates side-by-side images of motifs for each subcluster to help the users visualize the 3D structure similarities within a subcluster. Thus, GINClus will not only help to understand the existing motif families, it will also be useful in identifying new motif families by developing a structural pattern for unknown motif instances.

## Materials and methods

**Figure 1.**
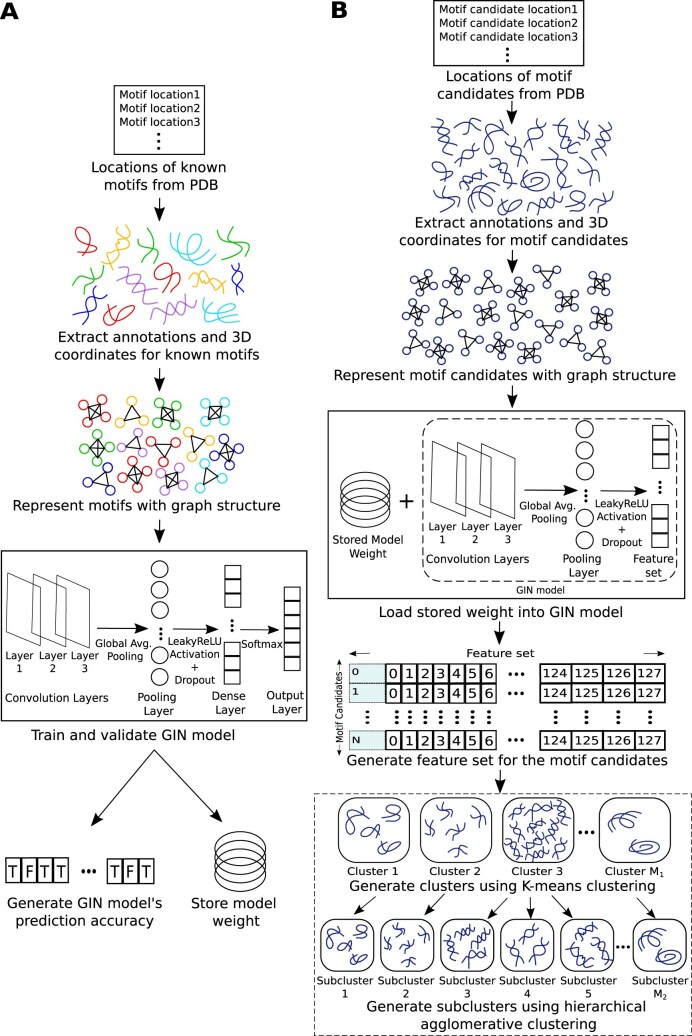
The overall pipeline used by GINClus. (**A**) Steps used to train the GIN model. (**B**) Steps used to cluster the motif candidates (loop regions) using the trained GIN model.

GINClus takes the locations of known motifs and motif candidates (loop regions) as input, and converts them into graph representations. Then, the GIN model is trained using the graph representations of known motif instances and using the trained model, an embedded feature set is generated for motif candidates. Finally, using this feature set, the motif candidates are clustered based on their structural similarities. Figure 1 shows the overall pipeline used by GINClus.

### Data collection

For creating the training dataset of GINClus, we collected known motifs from RNA structural motif families containing at least 30 motif members. Based on this criteria, we found six internal loop motif families known as Sarcin-ricin, Kink-turn, Tandem-shear, Hook-turn, E-loop, and C-loop and two hairpin loop motif families known as T-loop and GNRA tetraloop or GNRA. The locations of 313 well-known internal loop motifs from these six internal loop motif families are collected from Ge *et al.* [5] and RNA 3D Motif Atlas [4] by following the same procedure described in RNAMotifContrast [21]. After manual inspection, 20 internal loop motifs are filtered out due to incorrect motif family annotation, and the remaining 293 internal loop motif instances are used to train and test our model. Similarly, the locations of 403 well-known hairpin loop motifs from TL and GL motif families are collected from Ge *et al.* [5] and RNA 3D Motif Atlas [4]. The distribution of the motif instances is provided in the second column of Table 7. All these known internal loop and hairpin loop motif locations provided in RNAMotifContrast [21] were collected from NRList release 3.57 at resolution 4.0 Å [22]. So, to maintain consistency in the NRList versions for known motifs and motif candidates, we used the same NRList release for further data collection. The locations of 2656 internal loops and 2091 hairpin loops, also referred to as motif candidates, are collected from the representative RNA structures in the NRList release 3.57 at resolution 4.0 Å [22]. These larger sets of internal loop and hairpin loop motif candidates also include the known internal loop and hairpin loop motifs. It is also important to note that, hairpin loops, internal loops, and multi-branched loops (multiloops) were found during the loop collection process; however, we did not cluster multiloops using GINClus as enough training data are not available for multiloop motif families.

### Annotation and 3D coordinate extraction

The locations of known RNA structural motifs and RNA motif candidates are provided as input to GINClus. It then downloads the corresponding PDB and FASTA files from the PDB database [23]. For each of these PDB data, it collects FR3D annotations from the “RNA Structure Atlas” website [15] and DSSR annotations either using DSSR annotation tool v1.7.8-2018sep01 [24] or DSSR website [25]. GINClus then merges these two annotation outcomes to obtain a better quality base interaction information for the RNA structures. For some PDB files, conflicts might arise during the merging process when the annotations predict different base-pair interactions at the same site or predict that the same edge of one nucleotide was interacting with edges from two different nucleotides. GINClus resolves these conflicts by comparing the annotations across all nonredundant PDB structures in release 3.57 at 4.0 Å resolution and selecting the base interaction pattern that occurred most frequently. It then creates separate loop files with corresponding nucleotide sequences, base-pair and base-stacking interactions for known motifs and motif candidates. For each known motif and motif candidate, GINClus collects the 3D coordinates from the downloaded PDB files and generates a coordinate file.

### Graph representation

From the loop files and coordinate files, GINClus generates graph representations for each motif and motif candidate. These graph representations contain nucleotide distance information represented by a weighted adjacency matrix, nucleotide information as node features, base interaction information as edge features, and motif family information as graph label. The weight in the adjacency matrix represents the distance between two nucleotides, which is calculated by generating the Euclidean distance based on the RMSD values determined for the geometric centers of the backbone atoms of the nucleotides. Six atoms C3′, C4′, C5′, O3′, O5′, and P have been considered as the backbone atoms for each nucleotide [26], and their RMSD value is calculated using the Kabsch algorithm [27]. The nodes represent the nucleotides and node features represent the nucleotide type (adenine, cytosine, guanine, and uracil) using the one-hot encoding technique. Similarly, edge features represent the base-interaction and base-stacking information using the one-hot encoding technique.

**Figure 2.**
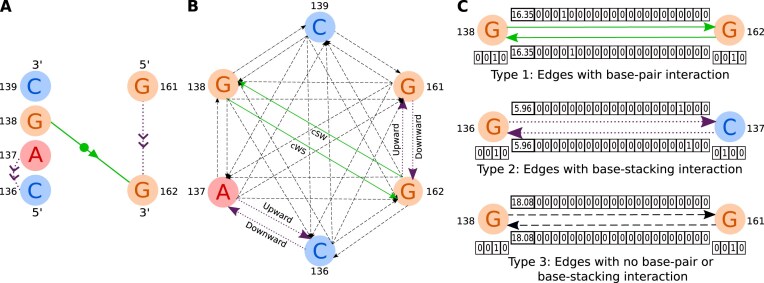
Graph representation of motif 1U9S_A:136-139_161-162. Here, A, G, and C represent adenine, guanine and cytosine, respectively. (**A**) 2D representation of the base-pair interaction (solid line) and the base-stacking (dotted line) of the motif using the notations proposed by [28]. (**B**) Connected directed graph representation of the motif. Here, solid lines represent base-pair interactions, dotted lines represent base-stackings, and dashed lines represent connection edges for the graph. (**C**) One hot encoding representation of node and edge features.

Based on the *cis* (c) or *trans* (t) orientation of glycosidic bonds, three base interaction edges (Watson–Crick edge denoted as “W,” Hoogsteen edge denoted as “H,” Sugar edge denoted as “S”), and directions (two different directions of base-pair interactions between two nucleotides), there are 18 different combinations of base-pair interactions: *cis* W/W, *cis* W/H, *cis* H/W, *cis* W/S, *cis* S/W, *cis* H/H, *cis* H/S, *cis* S/H, *cis* S/S, *trans* W/W, *trans* W/H, *trans* H/W, *trans* W/S, *trans* S/W, *trans* H/H, *trans* H/S, *trans* S/H, and *trans* S/S [28]. Base-stacking represents the relative orientation of two nucleotides with respect to each other and there are four types of base-stacking: upward, downward, inward, and outward [29]. Figure 2 shows an example of graph representation for the motif 1U9S_A:136-139_161-162. Two edges pointing toward the opposite directions (one inward and one outward), named connection edges, are added between all possible pairs of nodes making it a strongly connected directed graph. The edge feature vector contains information about pairwise nucleotide distance, base-pair interaction and base-stacking. The edge feature vector contains 23 elements in total where the first element contains the nucleotide distance, the next 18 elements contain information about base-pair interaction cWW, cWH, cHW, cWS, cSW, cHH, cHS, cSH, cSS, tWW, tWH, tHW, tWS, tSW, tHH, tHS, tSH, and tSS consecutively and the last four elements indicate information about whether base-stacking is upward, downward, inward, or outward.

### Train GIN model

The GIN model is implemented using the Spektral [30] python library developed based on TensorFlow and Keras. The GIN model first learns from the known motif instances (training data) that belong to the well-known motif families. These known motifs are represented using graphs where each graph is labeled with its family name. The dataset containing the known motifs are divided into three sets: training, validation, and testing data. We trained the model separately for internal loops and hairpin loops to generate separate datasets for internal loops and hairpin loops. If required, the model can also be trained with internal loops and hairpin loops at the same time. In case of internal loop motifs, there are a total of 293 known motifs where 253 are used for training, 20 used for validation, and 20 used for testing. For hairpin loop motifs, there are 406 known motifs among which, 354 are used for training, 26 used for validation, and 26 used for testing. GINClus was run on a 64-bit Ubuntu 20.04 system with an i7-8700 CPU and 64 GB RAM. The model took 152 seconds to train using 293 internal loops and 284 seconds to train using 406 hairpin loops. In GINClus, the model learns the graph network features by iteratively updating node representations based on the aggregation of its neighbors’ representations using three message-passing hidden perceptron layers. In each layer, the representation of the node is updated using Equation (1) [20].


(1) \begin{eqnarray*}
\mathrm{h_v^k} = MLP((1 + \epsilon ). h_v^{k-1} + \sum \limits _{u\epsilon N(v)} h_u^{k-1})
\end{eqnarray*}

In this equation, *v* represents the current node, *h* represents the hidden vector, *k* represents the layer number, *N*(*v*) represents the set of neighbor nodes to node *v*, and ε represents a learnable parameter, which is set to 0. Finally, global average pooling is used along with a LeakyReLU activation function for averaging the summation of all node representations from the same iterations to generate feature map for each motif. Here, we used the categorical cross-entropy loss function over softmax activation output to adjust model weights during training. The weight of the model with the highest accuracy is stored to train the GIN model in the next step.

### Feature set generation

In this step, the previously stored model weight is loaded into the GIN model and feature set is generated for all the motif candidates (RNA loop regions). To generate the feature set, the motif candidates are converted into graph representations and provided as input to the trained GIN model. The GIN model outputs 128 features for each RNA motif candidate. In case of internal loops, after training the model with 293 known motifs, we generated feature set for the 2656 internal loop motif candidates. Similarly for hairpin loops, after training the model with 403 known hairpin loop motifs, we generated the feature set for the 2091 hairpin loop motif candidates. The goal of this process is to generate a numerical feature set from the 3D structure and interaction-based graph representation of each motif candidate. Later, these features are used to cluster the RNA motif candidates based on their structural similarity.

### Cluster generation using *K*-means clustering

As the performance of clustering algorithms can vary widely based on the data distribution, different classical clustering algorithms such as *K*-means, *K*-median, Agglomerative, DBSCAN, Spectral, Gaussian mixture model, and Mean shift have been evaluated using the feature set generated for the 293 known internal loop motif instances. Adjusted Random Index (ARI) [31] and Adjusted Mutual Information (AMI) score [32] are used for evaluating the performance of the clustering algorithms. Both ARI and AMI values range between 0 to 1 where 1 indicates that the predicted clustering is highly similar to the original clustering. Table 1 shows the comparison of the performance of the clustering algorithms based on the known 293 internal loop motif instances. As can be seen from the table, clustering algorithm *K*-means, agglomerative, spectral, and mean shift have comparatively higher (>0.7) ARI and AMI values. Among them, *K*-means has the highest ARI value (0.92) with comparatively high AMI value. Hence, the *K*-means algorithm is selected to cluster the RNA motif candidates using the feature set generated based on their structural attributes.

**Table 1.** Performance comparison of different clustering algorithms

Algorithm	ARI	AMI	Parameters
*K*-means	0.92	0.73	n_clusters = 6
Agglomerative	0.76	0.75	distance_threshold=6
Mean shift	0.75	0.74	bandwidth=0.92
Spectral	0.73	0.73	n_clusters = 6
Gaussian mixture model	0.67	0.71	n_components=6
DBSCAN	0.51	0.58	eps=0.74, min_samples=5
*K*-median	0.25	0.54	n_clusters = 6

*K*-means is an unsupervised clustering algorithm that divides *N*-dimensional data objects into *K* number of nonoverlapping clusters where the number of clusters (value of *K*) has to be predefined [33]. While selecting the value of *K*, we had two goals: first, keeping the number of outliers in a cluster low; second, not generating too many clusters to avoid assigning similar motifs to separate clusters. We used evaluating metrics Silhouette Score and Davies–Bouldin Index to determine the number of clusters. Silhouette Score shows how closely connected the points in a cluster are. This ranges from 0 to 1 and a higher value represents better connected clusters. Davies–Bouldin Index represents the average similarity measure of the clusters to the other clusters they are most similar to. Its minimum possible value is 0 and the lower the value, the better the distance within different clusters.

**Figure 3.**
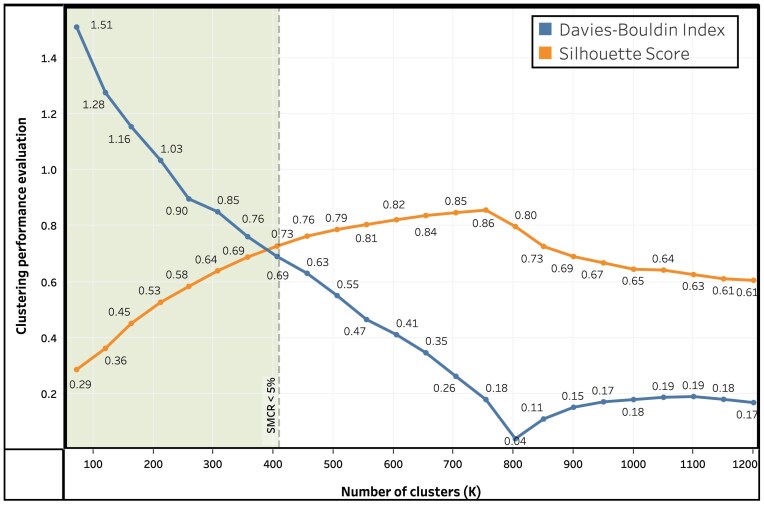
Evaluating *K*-means clustering algorithm performance on internal loop motif candidates for different number of clusters (*K*).

We ran the *K*-means algorithm on internal loop motif candidates for different values of *K* and generated the Silhouette Score and Davies–Bouldin Index as shown in Fig. 3. We only considered *K* values with Single Motif Cluster Ratio (SMCR) < 5% to avoid generating too many clusters containing a single motif. SMCR is calculated by dividing the number of clusters containing single motifs with the number of total clusters. As we can see from Fig. 3, for SMCR < 5%, we get the highest Silhouette Score (0.73) and the lowest Davies–Bouldin Index (0.69) for *K* = 400. So, using *K*-means clustering algorithm, the 2656 internal loop motif candidates are divided into 400 clusters based on their structural similarity. Similarly, we determined the value of *K* for hairpin loop motifs and the 2091 hairpin loop motif candidates are divided into 400 clusters using *K*-means clustering algorithm. To make sure that the clustering output remains consistent when the same data are clustered multiple times, we set the random initialization state to 0 while running *K*-means algorithm.

### Subcluster generation using agglomerative clustering

As the number of clusters is predefined in *K*-means, there is a possibility of clustering some structurally not so similar motifs in the same cluster. To handle this issue, we further divided the clusters generated using *K*-means into subclusters which is the final output generated by GINClus. As the number of clusters is not predefined in the hierarchical agglomerative clustering algorithm and according to Table 1, it has the second highest performance after *K*-means, it is selected as the subclustering algorithm. If all the members in a cluster are highly similar, then that cluster will not be divided any further using the subclustering algorithm. However, if there is structural dissimilarity among the members of a cluster, then the subclustering algorithm will divide it into multiple subclusters. Using the agglomerative clustering algorithm, the 2656 internal loops are finally divided into 406 subclusters and the 2091 hairpin loops are finally divided into 413 subclusters. The overall runtime was 490 seconds for internal loops and 477 seconds for hairpin loops using pregenerated loop annotation files. GINClus generates side-by-side and superimposed images of motifs for each subcluster using the open-source 3D structure rendering tool PyMOL [34].

### *Q*-score generation

GINClus generates *Q*-score (quality score) for each subcluster to evaluate the quality of the subclusters and to expedite subcluster analysis. To generate the *Q*-score, we determine the motif base interaction similarity using RNAMotifScanX and motif 3D structure similarity using TM-align. For a subcluster *i*, we generate the average alignment-score *SXsc*_*i*_, RMSD *SXd*_*i*_, and alignment length *SXal*_*i*_ using RNAMotifScanX. Similarly, we generate the average TM-score *TMsc*_*i*_, RMSD *TMd*_*i*_, and alignment length *TMal*_*i*_ using TM-align for subcluster *i*. Finally, we calculate the *Q* − *score*_*i*_ for subcluster *i* using Equations 2, 3, and 4. Here, *M*_*i*_ represents the number of motifs in the subcluster *i*. For any value *X*, here *X*_max_ represents the maximum *X* value among all the subclusters.


(2) \begin{eqnarray*}
SXQ_i = \frac{SXsc_i}{SXsc_{max}} + \frac{SXal_i}{SXal_{max}} + \frac{M_i}{M_{max}} - \frac{SXd_i}{SXd_{max}}
\end{eqnarray*}

(3) \begin{eqnarray*}
TMQ_i = \frac{TMsc_i}{TMsc_{max}} + \frac{TMal_i}{TMal_{max}} + \frac{M_i}{M_{max}} - \frac{TMd_i}{TMd_{max}}
\end{eqnarray*}

(4) \begin{eqnarray*}
Q-score_i = \frac{SXQ_i + TMQ_i}{2}
\end{eqnarray*}

## Results

### Ablation study

To evaluate the contribution of different algorithms in GINClus, we performed an ablation study using 293 known internal loop motifs. As shown in Table 2, the accuracy of GIN model is <60% for known internal loop motifs when we apply it based on just base interaction features or just 3D structure features. Combining both features, we get a higher accuracy for GIN model (69%). Again, using only GIN model with both features on 293 internal loop motifs, the precision, recall, F1-score, and accuracy is <70%. When we combine GIN with agglomerative clustering (AGL), the overall performance slightly improves. Combining GIN with *K*-means clustering, the accuracy improves up to 75%. But the performance significantly improves when we use GINClus where we combine GIN model first with *K*-means to generate clusters and then with AGL to generate subclusters. Using GINClus, the accuracy improved up to 87.88% while precision, recall, and F1-score improved up to 89%, 88%, and 88% respectively for known internal loop motifs.

**Table 2.** Ablation study of GINClus model based on known internal loop motifs

Model architecture	Precision	Recall	F1-score	Accuracy
GIN (based on base interaction features)	0.60	0.56	0.56	56%
GIN (based on 3D structure features)	0.55	0.51	0.51	51%
GIN (based on both features)	0.69	0.69	0.68	69%
GIN + AGL (based on both features)	0.72	0.70	0.70	70%
GIN + *K*-means (based on both features)	0.77	0.75	0.75	75%
GINClus: GIN + *K*-means + AGL (based on both features)	0.89	0.88	0.88	87.88%

### Clustering performance evaluation on known motifs

#### Performance on known motifs collected from NRList

In order to test the accuracy of GINClus, we randomly divided the 293 known internal loop motifs collected from the NRList release 3.57 at resolution 4.0 Å [22] into training, validation, and testing data. We used a 5-fold cross-validation technique where we split the input dataset randomly five times and ran the model. Each time, the training, validation, and testing dataset contained 253, 20, and 20 motifs, respectively. For each run, first, we generated the feature set of the test data using GIN model. Then we clustered them using *K*-means and subclusterd them using agglomerative clustering algorithm. Finally, comparing the true label with clustering labels, we generated precision, recall, F1-score, accuracy, and confusion matrix to evaluate the performance of GINClus. Table 3 shows the precision, recall, F1-score, and support values where the support value for each family represents the number of occurrences in the test dataset. The overall accuracy of GINClus is 87.88% for internal loop motifs.

**Table 3.** GINClus performance evaluation on known internal loop motifs

Motif family	Precision	Recall	F1-score	Support
CL	0.89	0.94	0.92	18
EL	0.73	0.92	0.81	12
HT	0.91	0.83	0.87	12
KT	0.90	0.86	0.88	21
SR	0.95	0.87	0.91	23
TS	0.86	0.86	0.86	14
Weighted average	0.89	0.88	0.88	100
Accuracy	87.88%			

**Figure 4.**
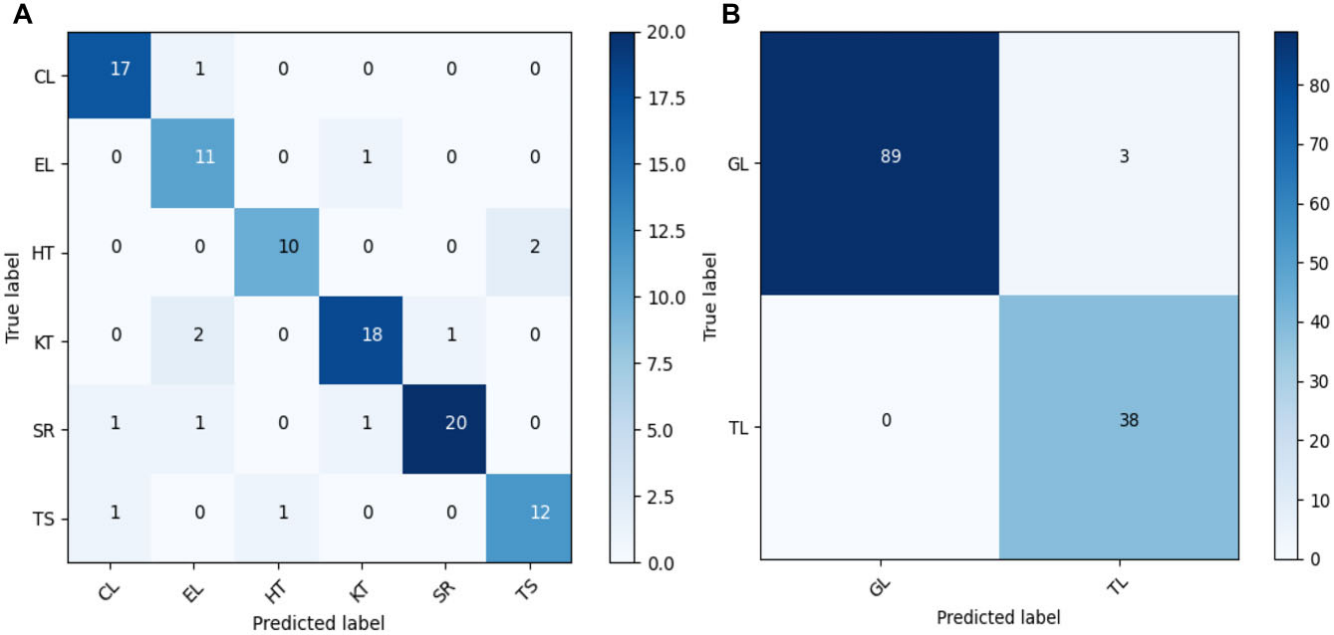
Confusion matrix of GINClus model on test data for (**A**) internal loop motifs and (**B**) hairpin loop motifs.

Similarly, as shown in Table 4, we evaluated the performance of GINClus on 406 known hairpin loop motifs collected from the nonredundant PDB list (NRList) release 3.57 at resolution 4.0 Å [22] using 5-fold cross-validation where each run contains 354 train, 26 validation, and 26 test data. For hairpin loop motifs, GINClus has an accuracy of 97.69%. The internal loop motif families have structural variations [21] and inter-family similarities [35], while the motifs belonging to hairpin loop motif families are short and have less diversity. So, clustering internal loops can be more challenging than clustering hairpin loops and as a result, GINClus achieves a lower accuracy for internal loops compared to hairpin loops. Figure 4 shows the confusion matrices for both internal loop motifs and hairpin loop motifs where the rows represent the true labels, the columns represent the predicted labels, the color scale changes from lighter to darker with increasing values, and the diagonal values represent the number of correctly predicted motif instances for each motif family. As we can see from the confusion matrices, in most of the cases, the model can predict the label of the motifs correctly despite the variations within each motif family and their inter-family similarities [35]. Since the known motif families contain a wide range of variation among them [21, 35], the training data contain significant amount of variations, which prevents the GIN model in GINClus from overfitting. The high accuracy of GINClus on the testing dataset also validates that the model is not overfitted.

**Table 4.** GINClus performance evaluation on known hairpin loop motifs

Motif family	Precision	Recall	F1-score	Support
GL	0.98	0.99	0.98	97
TL	0.97	0.94	0.96	33
Weighted average	0.98	0.98	0.98	130
Accuracy	97.69%			

#### Performance on known motifs collected from 23S rRNA

23S ribosomal RNA (rRNA) contains many well-defined motifs and hence is popularly used for benchmarking [11, 36]. To evaluate the clustering performance of GINClus on 23S rRNA, we collected the known motifs from PDB 1S72 chain 0. From these motifs, we picked the motifs from the SR and KT families, since the other motif families contain less than five motifs. Next, using the previously trained GINClus model (trained using 293 known internal loop motifs), we generated a feature set for these newly picked motifs from PDB 1S72 chain 0 and divided them into subclusters. While running the model, we set *K* = 4 after evaluating *K*-means performance for different numbers of clusters. Table 5 shows the subcluster output generated by GINClus for the motifs collected from PDB 1S72 chain 0. Since the motifs of the known families have structural variations [21], motifs of SR and KT families are divided into multiple clusters. Also, both SR and KT motifs share structure similarities (similar kink shape) and common base-pair interactions (A-G tH/S and G-A tS/H). So, in some cases, SR and KT motifs have been clustered together (subclusters 2 and 7 in Table 5). Supplementary Fig. S1 shows base-pair interactions and 3D structures of SR and KT motifs assigned to subclusters 2 and 7 in Table 5. However, as we can see from Table 5, in most of the cases GINClus is able to separately cluster motifs from SR and KT family. This shows that GINClus can cluster RNA structural motifs collected from any RNA chain.

**Table 5.** GINClus clustering output for the known motifs from PDB 1S72 23S rRNA

Motif location	Subcluster ID	Family
1S72_0:1971-1974_2008-2010*	0	SR
1S72_0:910-912_1292-1295*	0	SR
1S72_0:380-384_405-408*	1	SR
1S72_0:1147-1155_1212-1216	2	KT
1S72_0:158-164_171-178	2	SR
1S72_0:565-572_585-593	2	SR
1S72_0:1367-1373_2052-2057	3	SR
1S72_0:210-216_224-229	3	SR
1S72_0:2689-2695_2700-2705	3	SR
1S72_0:245-249_260-266	4	KT
1S72_0:43-50_111-113	4	KT
1S72_0:936-940_1026-1034	4	KT
1S72_0:462-467_474-479*	5	SR
1S72_0:952-956_1011-1015	5	SR
1S72_0:1312-1319_1338-1342	6	KT
1S72_0:2822-2829_2911-2914	6	KT
1S72_0:2845-2855_2903-2906	6	KT
1S72_0:77-81_93-100	6	KT
1S72_0:1587-1592_1602-1608	7	KT
1S72_0:290-296_355-362	7	SR

*Part of a multiloop junction.

#### Clustering performance comparison on known motifs

To compare the clustering performance of GINClus with other RNA motif clustering methods, we collected a dataset used by both RNAMSC and LENCS. This dataset contains 5S (*Haloarcula marismortui*, PDB 1S72 chain 9), 16S (*Thermus thermophilus*, PDB 1J5E chain A), and 23S (*Haloarcula marismortui*, PDB 1S72 chain 0) rRNAs. We clustered the motifs from this dataset using the previously trained GINClus (trained using 293 known internal loop motifs) and compared the clustering output with RNAMSC, LENCS, and RNA 3D Motif Atlas (release 3.93). The known motif instances from this dataset (shown in Supplementary Table S1) correspond to GNRA, SR, KT, TS, HT, CL, EL, and reverse Kink-turn motifs. The motifs identified by GINClus, RNAMSC, LENCS, and RNA 3D Motif Atlas among these known motifs are marked in Supplementary Table S1. The clusters generated by GINClus that correspond to these known motifs are shown in Supplementary Table S2. The clustering output for LENCS [10] and RNAMSC [11] for these known motifs are collected from their related publications. In case of RNA 3D Motif Atlas, the corresponding known motifs are gathered from their comprehensive collection of motifs [4]. However, as RNA 3D Motif Atlas uses the representative RNA chains from the NRList [22], it contains motifs from PDB 4V9F chain 0 (23S rRNA), PDB 4V9F chain 9 (5S rRNA), and PDB 4LFB chain A (16S rRNA) which, according to NRList release 3.57, are equivalent to PDB 1S72 chain 0, PDB 1S72 chain 9, and PDB 1J5E chain A, respectively. Therefore, we collected the annotated rRNA motifs from PDB 4V9F and 4LFB from RNA 3D Motif Atlas and mapped those motifs to PDB 1S72 and 1J5E, respectively. The motifs with the same known motif annotations are grouped together as shown in Supplementary Table S3. In RNA 3D Motif Atlas, EL motifs are annotated as tSH-tHW-tHS, TS motifs are annotated as triple/double sheared, and HT motifs are annotated as UAA/GAN. The comparison between the clustering results of LENCS, RNAMSC, RNA 3D Motif Atlas, and GINClus for known motifs collected from 5S, 16S, 23S rRNA of PDB 1S72, and 1J5E is summarized in Table 6. The two SR motifs (1S72_0:380-384_405-408, 1S72_0:451-467_474-479) that are part of multiloop junctions have been excluded from the comparison with RNA 3D Motif Atlas in Table 6 as RNA 3D Motif Atlas clusters the loops of varying junctions separately and, therefore, does not identify SR motifs from multiloop junctions. For finding similar RNA motifs, LENCS and RNAMSC can only consider base-pair interactions while RNA 3D Motif Atlas and GINClus can consider both base-pair interactions and 3D structures of motifs. As shown in Table 6, GINClus outperforms these motif clustering methods and gains higher sensitivity (SN) and specificity (SP).

**Table 6.** Clustering performance comparison of GINClus with LENCS, RNAMSC and RNA 3D Motif Atlas

Motif family	LENCS		RNAMSC		RNA 3D Motif Atlas		GINClus	
	SN^b^ (%)	SP^c^ (%)	SN (%)	SP (%)	SN (%)	SP (%)	SN (%)	SP (%)
SR	66.67 (8/12)	100 (8/8)	100 (12/12)	100 (12/12)	90.00 (9/10*)	90.00 (9/10)	100 (12/12)	100 (12/12)
KT	20.00 (2/10)	100 (2/2)	50.00 (5/10)	100 (5/5)	70.00 (7/10)	87.50 (7/8)	100 (10/10)	90.91 (10/11)
TS	100 (6/6)	75.00 (6/8)	33.33 (2/6)	100 (2/2)	83.33 (5/6)	83.33 (5/6)	83.33 (5/6)	100 (5/5)
HT	100 (3/3)	60.00 (3/5)	66.67 (2/3)	100 (2/2)	33.33 (1/3)	33.33 (1/3)	66.67 (2/3)	100 (2/2)
EL	100 (4/4)	57.14 (4/7)	100 (4/4)	66.67 (4/6)	100 (4/4)	80.00 (4/5)	100 (4/4)	80.00 (4/5)
CL	50.00 (2/4)	100 (2/2)	75.00 (3/4)	100 (3/3)	75.00 (3/4)	75.00 (3/4)	100 (4/4)	100 (4/4)
rKT^a^	100 (3/3)	42.80 (3/7)	100 (3/3)	100 (3/3)	–	–	100 (3/3)	100 (3/3)
GNRA	–	–	64.29 (18/28)	94.74 (18/19)	92.86 (26/28)	92.86 (26/28)	89.29 (25/28)	96.15 (25/26)
Average	76.67	76.42	73.66	95.18	77.79	77.43	**92.41**	**95.88**

^a^rKT refers to reverse Kink-turn motif family, ^b^Sensitivity (SN) = True positive/Number of total known motifs, ^c^Specificity (SP) = True Positive/Number of total cluster members, *Excludes the two SR motifs that are part of multiloop junctions from the number of total SR motifs.

### Clustering performance evaluation on motif candidates

#### Structural similarity within each subcluster

We performed statistical analysis on known motif families based on both base interaction and 3D structure similarity to determine how the RMSD values vary among structurally similar motifs. We calculated the range within two standard deviation for the known motif families. Approximately 95% of the known motif pairwise RMSD values fall within this range. So, if any RNA loop pair has RMSD below the upper-bound of this range, then we consider them to be structurally similar. Based on base interaction similarity, the upper-bound of range within two standard deviation is 4.72 Å for internal loops and 2 Å for hairpin loops. Similarly, based on 3D structure similarity, the upper-bound of the range is 2.46 Å for internal loops and 1.55 Å for hairpin loops. After filtering the subclusters that contain single RNA loop, there are a total of 387 internal loop subclusters and 293 hairpin loop subclusters. Based on base-interaction similarity, 92.76% internal loop subclusters and 67.57% hairpin loop subclusters have average pairwise RMSD below the upper-bound of the two standard deviation range. Similarly, 97.67% internal loop subclusters and 84.98% hairpin loop subclusters have average pairwise RMSD below the upper-bound based on 3D structure similarity. This shows that most of our internal loop and hairpin loop subclusters contain RNA loops that are structurally similar to each other. Supplementary Figs S2 and S3 show the distribution of number of subclusters over average pairwise RMSD value based on both base-interaction and 3D structure similarity for internal loops and hairpin loops, respectively. Supplementary Table S4 shows the detailed statistical analysis result on the pairwise RMSD of known motif family loops.

#### Subclusters generated for known motif families

We analyzed the 406 internal loop subclusters and 413 hairpin loop subclusters generated by GINClus to find subclusters that contain motifs from known motif families (KT, SR, TS, HT, EL, CL, GL, and TL) used for training GINClus. We shortlisted the subclusters that contain one or multiple motifs from the given 293 known internal loop and 406 known hairpin loop motif list. We found 238 such subclusters and we analyzed the motif candidates (loop regions) belonging to each of these subclusters. We closely looked at the base-pair interactions and 3D structures of these loops and if they possess structural similarity with any of the known motif families, we considered them as new members of that family. Among those 238 subclusters, if any subcluster has >60% of loop members belonging to a single known motif family, we assigned that subcluster to that specific motif family. The loops in a subcluster that do not share the common features with the majority of the motif members are considered to be the outliers in that subcluster. We also filtered out loops containing missing or conflicting annotations from these subclusters. Following these criteria, we found 23 subclusters belonging to SR motif family, 15 subclusters belonging to KT, 14 subclusters belonging to TS, 11 subclusters belonging to HT, 16 subclusters belonging to CL, 14 subclusters belonging to EL, 19 subclusters belonging to TL, and 42 subclusters belonging to GL. Similar to internal loop and hairpin loop, GINClus can generate clusters for multiloops based on structural similarity. But due to the unavailability of training data for RNA multiloop motif families, we have not evaluated the performance of GINClus on multiloops. Both [4] and [5] proposed some clusters for RNA multiloops. But on average, these clusters contain 5–7 motifs each; this number is too small to train GINClus.

Table 7 shows a summary of the known motif family subclusters. As we can see from the table, using GINClus, we found 81 new instances of SR, 66 new instances of KT, 74 new instances of TS, 47 new instances of HT, 92 new instances of EL, 102 new instances of CL, 85 new instances of TL, and 380 new instances of GL motif family. For internal loop motif families, Motif clustering accuracy (MCA) is > 70% and for hairpin loop motif families, MCA is > 80%. MCA for each family is calculated by dividing the number of known motifs of a family in the corresponding family clusters by the number of total known motifs for that family. The high MCA values indicate that the proposed GINClus model can successfully cluster together most of the RNA structural motifs of the known motif families of internal loop and hairpin loop. Supplementary Tables S5–S12 show the list of motifs belonging to the known motif family subclusters.

**Table 7.** Summary of clusters generated for motif families known to GINClus (used in GIN model training)

Motif family	No. of total known motifs	No. of corresponding motif family clusters	In corresponding motif family clusters				Motif clustering accuracy (MCA)
			No. of total	No. of known	No. of new	No. of	
			motifs	motifs	motifs	outliers	
SR	69	23	162	55	81	26	79.71%
KT	65	15	136	46	66	24	70.77%
TS	43	14	133	34	74	25	79.07%
HT	33	11	91	25	47	19	75.76%
EL	45	16	151	35	92	24	77.78%
CL	38	14	169	30	102	37	78.95%
TL	113	19	211	95	85	30	84.07%
GL	290	42	694	286	380	28	98.62%

As mentioned in RNAMotifContrast [21], RNA structural motifs belonging to the same family have base-pair interaction and structural variations and can be further divided into subfamilies. Due to their variations, the motifs from the same family are divided into multiple subclusters. Supplementary Table S13 shows that SR motif family contains 23 corresponding subclusters and base-pair interaction variations exist within these subclusters. While analyzing the variations, we did not consider the outliers within SR subclusters (only considered either previously known or new instances of SR motifs). From the table, we can see that most of the SR subclusters have an average RMSD < 2Å and average alignment length ≥ 6. This further shows how highly structure-wise similar the motifs are within each SR subcluster. Overall, GINClus is not only capable of clustering motifs from known families, it can also capture the variations within known motif families which can be overlooked by alignment-based motif searching tools.

#### Subclusters generated for unknown motif families

As mentioned before, GINClus employs the graph isomorphism neural network (GIN), which uses semi-supervised learning. So, it is capable of clustering RNA structural motifs from families that it was not trained with. To assess the performance of GINClus in clustering RNA structural motif families that it is not familiar with, we focused on motif families reverse Kink-turn, Tetraloop-receptor, L1-complex, and Rope-sling. These motif families have not been used in training GINClus as these families did not contain enough training data. Since reverse Kink-turn, Tetraloop-receptor, L1-complex, and Rope-sling are all internal loop motif families, we analyzed the 406 internal loop subclusters to asses the performance of GINClus. We used the same criteria that we previously used for finding corresponding subclusters for the known motif families. From Table 8, we can see that the GINClus model can successfully cluster motifs from the families reverse Kink-turn, Tetraloop-receptor, and L1-complex with an MCA ≥ 80%. This proves that GINClus is capable of clustering RNA structural motifs from families that it was not trained with. However, the model did not perform well in case of Rope-sling (MCA < 50%). According to the definition provided by [11], Rope-sling motifs generally have two nucleotides on one side and a long strand on the other side with a cS/H bond between two consecutive nucleotides. Since many other RNA structural motif instances also have a short strand-long strand structure and contain the cS/H bond between two consecutive nucleotides, it’s hard for GINClus to separate Rope-sling motifs from other motif instances. This explains the reason for its poor performance in case of Rope-sling. The lists of subclusters belonging to these four motif families are shown in Supplementary Tables S14–S17.

**Table 8.** Summary of clusters generated for motif families unknown to GINClus (not used in GIN model training)

Motif family	No. of total known motifs	No. of corresponding motif family clusters	In corresponding motif family clusters				Motif clustering accuracy (MCA)
			No. of total	No. of known	No. of new	No. of	
			motifs	motifs	motifs	outliers	
reverse Kink-turn	8	6	28	7	13	8	87.5%
Tetraloop-receptor	11	1	17	9	3	5	81.81%
L1-complex	5	2	7	4	2	1	80%
Rope-sling	9	3	9	3	4	2	33.33%

### Identifying new RNA structural motif families

Since GINClus can successfully cluster RNA structural motifs of families unknown to its GIN model, the output subclusters might contain potential new RNA structural motif families. To test this theory, we analyzed the output 406 subclusters of internal loops separately based on base interaction and 3D structure similarity. For base interaction similarity, we first sorted all the 406 subclusters based on their average alignment-score (high to low), RMSD (low to high), and alignment length (high to low) generated using the tool RNAMotifScanX. Then, we filtered out the previously identified known motif family subclusters from the sorted list. Finally, we selected the top 10 subclusters from the sorted list to check if there are any potential new motif families. After analyzing the top 10 subclusters, we found two new RNA structural motif families which we are referring to as Double-kink-turn (subclusters 53, 226, and 269) and 3-point-turn (subcluster 377) as shown in Table 9. We also found four new subclusters (subclusters 174, 184, 288, and 294) for SR family and two new subclusters (subclusters 238 and 321) for KT family. These subclusters have not been identified before as they did not contain any motif from the 293 known motif instances. Supplementary Table S18 shows the list of motifs belonging to these top 10 subclusters identified based on base interaction similarity. The motifs of the Double-kink-turn family have a short strand with four nucleotides and a long strand with 14 nucleotides with the base-pairs G-G tW/H, U-A tW/H, and G-A tS/H in the long strand. These motifs also contain base-pairs A-A tH/S, A-A cS/W, A-A tH/H, and G-G tS/S between the two strands. We named it Double-kink-turn because the 3D structure of these motifs contain two kink-shaped bends. The motifs from the new 3-point-turn motif family have two long strands and three consecutive tH/W bonds between the two strands. They also contain a cH/W bond that forms a two nucleotide UA bulge with a tH/W base-pair interaction. We named this family 3-point-turn because the 3D structure of the motifs take turns at three points.

**Table 9.** Top 10 motif families based on base interaction similarity and top 10 motif families based on 3D structure similarity

Based	Cluster	Family name	No. of cluster	No. of	Avg. motif	AS^a^/	TM-score/
on	number	(known/proposed)	members	outliers	length	RMSD/AL^b^	RMSD/AL
Base	269	Double-KT	2	0	18	307.10/0.58/18.00	0.60/0.56/18.00
interaction	53	Double-KT	3	0	18	254.63/0.90/18.00	0.46/0.88/18.00
similarity	288	Sarcin-ricin	2	0	14	197.30/0.66/14.00	0.47/0.67/14.00
	226	Double-KT	3	0	18	187.15/0.99/16.50	0.42/1.03/18.00
	294	Sarcin-ricin	2	0	13	166.20/0.37/09.00	0.67/0.43/13.00
	238	Kink-turn	2	0	16	155.30/0.50/16.00	0.57/0.50/16.00
	184	Sarcin-ricin	3	0	13	143.50/0.66/11.67	0.49/0.72/12.33
	321	Kink-turn	2	0	16	135.20/0.28/16.00	0.73/0.32/16.00
	377	3-point-turn	2	0	16	119.40/0.66/16.00	0.44/0.63/16.00
	174	Sarcin-ricin	2	0	13	119.30/0.36/13.00	0.67/0.37/13.00
3D	11	Anchor-loop	23	0	6	42.99/0.21/05.12	0.83/0.23/05.83
structure	20	Bow-loop	19	0	5	25.50/0.28/05.00	0.79/0.28/05.00
similarity	45	Parabolic-loop	8	0	5	25.64/01.14/04.84	0.73/0.46/05.00
	323	Tau-loop	2	0	9	24.90/0.35/07.00	0.71/0.34/09.00
	157	Beta-loop	8	0	7	44.55/0.35/06.50	0.71/0.38/07.00
	393	Double-handle-loop	2	0	14	75.60/0.39/14.00	0.71/0.40/14.00
	198	Pocket-loop	6	1	5	24.37/0.81/04.00	0.70/0.26/03.87
	243	Cross-loop	7	1	5	27.46/0.21/03.81	0.67/0.29/04.29
	143	Pi-loop	4	0	16	97.20/0.41/16.00	0.66/0.44/16.00
	299	Sine-loop	3	0	12	36.90/0.44/12.00	0.63/0.44/12.00

^a^AS refers to alignment-score, and ^b^AL refers to alignment length.

**Figure 5.**
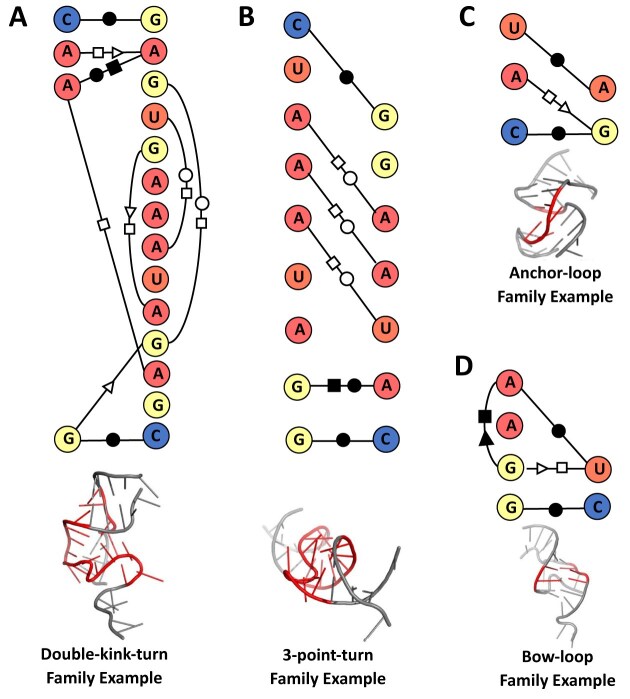
Panels (**A**–**D**) show the base-pair interaction (top) and 3D structure (bottom) of example motifs from the families double-KT, 3-point-turn, anchor-loop, and bow-loop, respectively. Base-pair interaction notations used here are collected from [28]. Here, A, C, G, and U represent adenine, cytosine, guanine, and uracil, respectively.

We followed a similar process to find new RNA families based on 3D structure similarity. We sorted the 406 subclusters based on average TM-score (high to low), RMSD (low to high), and alignment length (high to low) generated by 3D structure alignment tool RNA-align, filtered out the previously identified known motif family subclusters, and selected the top 10 subclusters. After analyzing the top 10 subclusters, we proposed 10 new motif families based on similar structural features as shown in Table 9. Among these, the families Anchor-loop (23 motifs) and Bow-loop (19 motifs) contained a high number of motif members and so, we are going to discuss their structural features. The Anchor-loop family (subcluster 11) motifs have a 3D structure shaped like an anchor and they contain the base-pairs G-U tS/H and G-A cS/H. The structure of the motifs from the Bow-loop family (subcluster 20) slightly look like archery bows and they contain the base-pair interaction A-G tH/S. Figure 5 shows the base-pair interactions and 3D structures of example motifs for the families double-KT, 3-point-turn, Anchor-loop, and Bow-loop. The list of motifs belonging to the top 10 subclusters identified based on 3D structure similarity is shown in Supplementary Table S19. In total, we proposed 12 new RNA structural motif families based on base interaction and 3D structure similarity.

### NRList release 3.364

In this paper, we clustered RNA loops from NRList release 3.57 which contained 2656 internal loops and 2091 hairpin loops. Since the number of RNA structures at RCSB PDB [23] are increasing at a rapid rate, NRList has newer versions containing higher number of RNA structures. To generate clustering output for newer version of data, we collected RNA loops from NRList release 3.364 (released on 4 December 2024). From NRList release 3.364, we collected 13866 internal loops and 9358 hairpin loops. Using GINClus, a total of 974 subclusters were generated for the internal loops and 667 subclusters were generated for the hairpin loops. The new clustering output (NRList release 3.364) along with the previously generated clustering output (NRList release 3.57) are available on GitHub.

## Discussion and conclusion

GINClus is a highly efficient and easy-to-use RNA structural motif clustering tool. It is independent of pairwise structural motif alignment and comparison, which makes it computationally efficient. The motifs belonging to the same family can have similar base interactions and/or 3D structures, so considering just base interaction or 3D structure can limit the search or clustering results and increase the number of false negatives. GINClus takes into consideration both the base interactions and the 3D structures of RNA motifs. As a result, it can find more motif instances of an RNA structural motif family and capture the variations that exist within the family. The ability of the GIN model to differentiate nonisomorphic graphs enables the tool GINClus to recognize motifs with similar structures irrespective of different node order representations. GINClus has the capability to find new motif instances of existing motif families as well as motif instances of new RNA structural motif families. As shown in the study, GINClus is successfully able to cluster the motif families known to GINClus (SR, KT, TS, HT, EL, CL, TL, and GL) with an MCA ≥ 70% and the motif families unknown to GINClus (reverse Kink-turn, Tetraloop-receptor, and L1-complex) with an MCA ≥ 80%. Using the clustering output generated by GINClus, we identified 12 new RNA structural motif families. It also generates side-by-side images of RNA structural motifs within a subcluster which makes it easier for users to visualize and analyze the clustering output.

The RNA structure research field is highly evolving and new RNA structural motif instances are being discovered on a regular basis. GINClus can be trained with these newly evolving RNA structural motif instances and families which makes it a highly scalable and adaptive tool. The clustering output generated by GINClus can be analyzed further to find conserved RNA regions with similar secondary and tertiary features and similar functionality. This will pave the way towards easily and efficiently finding new RNA structural motif families based on both structural and functional similarities. One limitation of the GINClus tool is that its performance depends on the availability of high quality training data. In future, we plan to include the newly found motif instances by GINClus in the training dataset and preprocess the training data to improve data quality which might further improve the performance of the tool. GINClus has the ability to handle internal loops, hairpin loops, and multiloops. However, in this paper, we only clustered internal loops and hairpin loops using GINClus as enough training data are not available for clustering multiloops. Overall, GINClus can make significant contributions to the field of RNA structure by making RNA structural motif clustering process way more efficient and simplified, and by introducing new RNA structural motif families with specific structural features and functionalities.

## Supplementary Material

lqaf050_Supplemental_File

## Data Availability

The source code for GINClus, along with input files containing motif locations, clustering results, and images of clustered motifs discussed in this paper, is available on GitHub (https://github.com/ucfcbb/GINClus) and Zenodo (https://doi.org/10.5281/zenodo.15220251).
