# Supplementary material for: GINClus: RNA structural motif clustering using graph isomorphism network
Source: NAR Genom Bioinform. 2025 Apr 26;7(2):lqaf050. doi: 10.1093/nargab/lqaf050 (PMC12034103; doi:10.1093/nargab/lqaf050)
Supplement: lqaf050_Supplemental_File [file lqaf050_supplemental_file.pdf]

Supplementary Data for "GINClus: RNA structural motif clustering using graph isomorphism network"

Nabila Shahnaz Khan, Md Mahfuzur Rahaman, Shaojie Zhang *

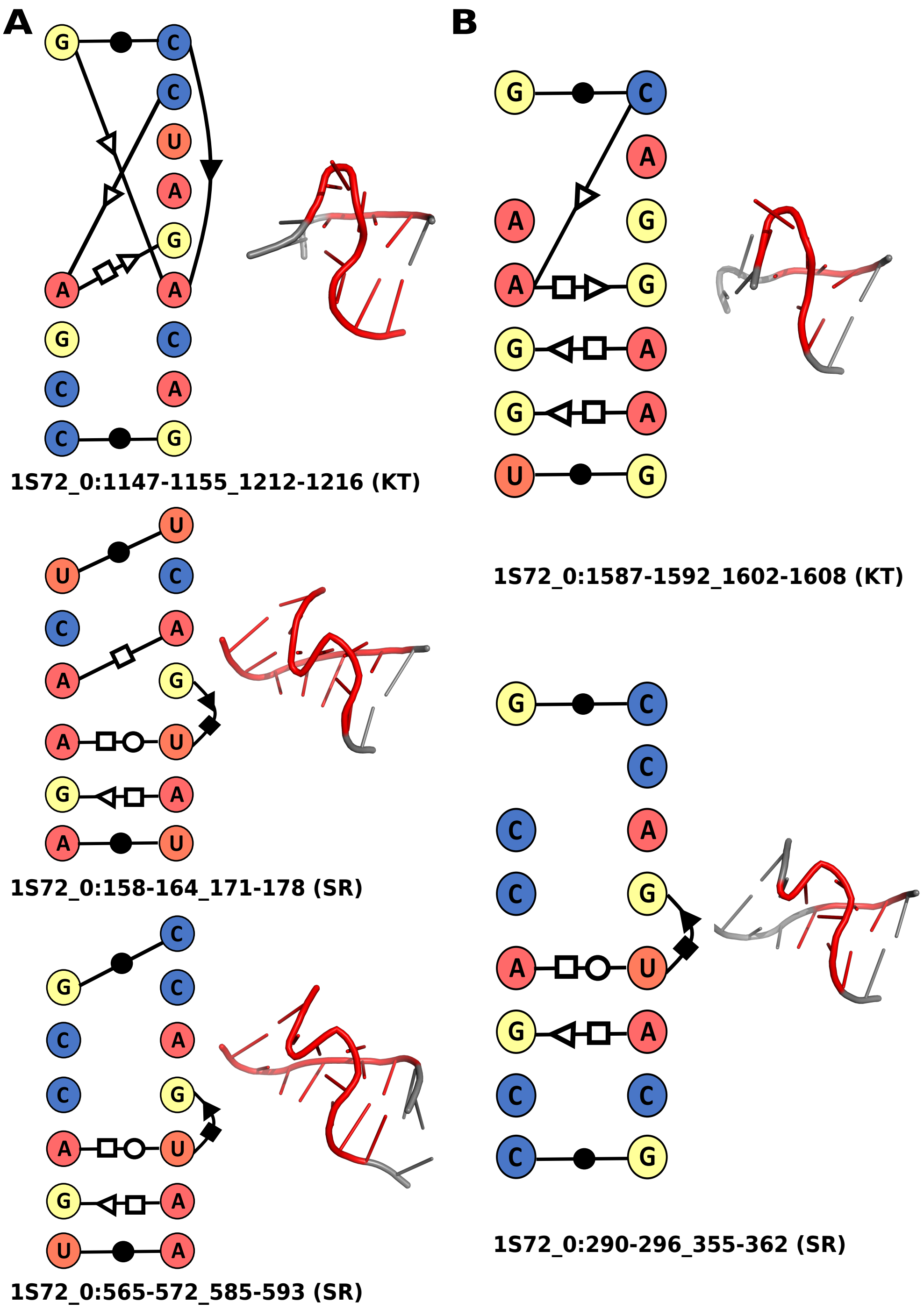

Figure S1: Base-pair interactions and 3D structures of Sarcin-ricin (SR) and Kink-turn (KT) motifs assigned to subcluster 2 and subcluster 7 in Table 5. (A) shows the base-pair interactions (left) and 3D structures (right) of motifs in subcluster 2. (B) shows the base-pair interactions (left) and 3D structures (right) of motifs in subcluster 7.

*To whom correspondence should be addressed. Email: shzhang@cs.ucf.edu

Table S1: List of known motifs for comparing the performance of GINClus, RNAMSC, LENCS and RNA 3D Motif Atlas

Motif Family	Motif location	GINClus	RNAMSC	LENCS	RNA 3D Motif Atlas
Sarcin-ricin	1J5E_A:1345-1350_1372-1376	*	*	*	-
	1J5E_A:887-894_905-910	*	*	*	*
	1S72_0:1367-1373_2052-2057	*	*	*	*
	1S72_0:158-164_171-178	*	*	*	*
	1S72_0:210-216_224-229	*	*	*	*
	1S72_0:2689-2695_2700-2705	*	*	*	*
	1S72_0:290-296_355-362	*	*	-	*
	1S72_0:380-384_405-408	*	*	*	(-)
	1S72_0:451-467_474-479	*	*	*	(-)
	1S72_0:562-572_585-595	*	*	-	*
	1S72_0:952-956_1011-1015	*	*	-	*
	1S72_9:75-81_101-106	*	*	-	*
Kink-turn	1J5E_A:683-688_699-707	*	*	-	*
	1S72_0:1147-1155_1212-1216	*	*	-	*
	1S72_0:1312-1319_1338-1342	*	*	-	*
	1S72_0:1586-1593_1601-1609	*	-	-	*
	1S72_0:244-250_259-267	*	-	-	*
	1S72_0:2822-2829_2911-2914	*	-	-	-
	1S72_0:2845-2855_2903-2906	*	-	-	-
	1S72_0:43-50_111-113	*	-	-	-
	1S72_0:77-81_93-100	*	*	*	*
	1S72_0:936-940_1026-1034	*	*	*	*
Tandem-shear	1J5E_A:1416-1419_1481-1484	*	*	*	*
	1J5E_A:1431-1435_1466-1469	*	-	*	-
	1S72_0:516-522_21-27	*	-	*	*
	1S72_0:794-798_815-819	-	-	*	*
	1S72_0:2501-2505_2515-2519	*	-	*	*
	1S72_0:2873-2876_2881-2884	*	*	*	*
Hook-turn	1S72_0:1457-1460_1483-1485	*	-	*	*
	1S72_0:2242-2245_2256-2260	*	*	*	-
	1S72_0:2672-2676_2809-2817	-	*	*	-
E-loop	1J5E_A:580-584_757-761	*	*	*	*
	1J5E_A:779-783_799-803	*	*	*	*
	1S72_0:1542-1546_1639-1643	*	*	*	*
	1S72_0:705-709_719-723	*	*	*	*
C-loop	1J5E_A:371-375_389-390	*	*	-	*
	1S72_0:1425-1429_1437-1439	*	*	*	*
	1S72_0:2717-2721_2761-2763	*	*	*	*
	1S72_0:958-963_1005-1008	*	-	-	-
Reverse Kink-turn	1S72_0:1122-1134_1228-1242	*	*	*	-
	1S72_0:1521-1529_1662-1665	*	*	*	-
	1S72_0:1531-1534_1657-1660	*	*	*	-
GNRA	1J5E_A:1076-1081	*	*	-	*
	1J5E_A:296-301	*	*	-	*
	1J5E_A:1265-1270	-	*	-	*
	1J5E_A:158-163	*	*	-	*
	1J5E_A:379-384	*	*	-	*
	1J5E_A:691-696	*	-	-	-
	1J5E_A:726-731	*	-	-	*
	1J5E_A:897-902	*	*	-	*
	1J5E_A:1012-1017	*	*	-	*
	1S72_0:1706-1712	*	-	-	*
	1S72_0:2248-2253	*	*	-	*
	1S72_0:2629-2634	*	*	-	*
	1S72_0:2695-2700	*	*	-	*
	1S72_0:468-473	*	*	-	*
	1S72_0:493-499	*	-	-	*
	1S72_0:576-581	*	*	-	*
	1S72_0:1054-1060	*	-	-	*
	1S72_0:1275-1281	*	-	-	*
	1S72_0:1326-1331	*	-	-	*
	1S72_0:1468-1474	*	-	-	*
	1S72_0:1628-1633	*	*	-	*
	1S72_0:1793-1799	-	-	-	*
	1S72_0:1862-1867	*	*	-	*
	1S72_0:252-257	*	*	-	*
	1S72_0:2876-2881	*	*	-	*
	1S72_0:481-487	-	-	-	-
	1S72_0:690-695	*	*	-	*
	1S72_9:89-94	*	*	-	*

The third to sixth column indicates whether the motif is identified by the corresponding method. * indicates identified, - indicates not identified and (-) indicates not identified by RNA 3D Motif Atlas due to being part of a multiloop junction.

Table S2: Clusters generated by GINClus for known motifs collected from rRNA of 1S72 and 1J5E

GNRA cluster			
1J5E_A:1076-1081	1J5E_A:1012-1017	1S72_0:576-581	1S72_0:252-257
1J5E_A:296-301	1S72_0:1706-1712	1S72_0:1054-1060	1S72_0:2876-2881
1J5E_A:158-163	1S72_0:2248-2253	1S72_0:1275-1281	1S72_0:690-695
1J5E_A:379-384	1S72_0:2629-2634	1S72_0:1326-1331	1S72_0:1769-1774
1J5E_A:691-696	1S72_0:2695-2700	1S72_0:1468-1474	1S72_9:89-94
1J5E_A:726-731	1S72_0:468-473	1S72_0:1628-1633	
1J5E_A:897-902	1S72_0:493-499	1S72_0:1862-1867	
**Sarcin-ricin cluster**	**Kink-turn cluster**	**Tandem-shear cluster**	**Hook-turn cluster**
1J5E_A:887-894_905-910	1J5E _A:683-688_699-707	1J5E_A:1416-1419_1481-1484	1S72_0:1457-1460_1483-1485
1J5E_A:1345-1350_1372-1376	1S72 _0:244-250_259-267	1J5E_A:1431-1435_1466-1469	1S72_0:2242-2245_2256-2260
1S72_0:158-164_171-178	1S72_0:936-940_1026-1034	1S72_0:516-522_21-27	
1S72_0:2689-2695_2700-2705	1S72_0:77-81_93-100	1S72_0:2873-2876_2881-2884	
1S72_0:210-216_224-229	1S72 _0:1586-1593_1601-1609	1S72_0:2501-2505_2515-2519	
1S72_0:562-572_585-595	1S72 _0:1312-1319_1338-1342		
1S72_0:1367-1373_2052-2057	1S72_0:1147-1155_1212-1216		
1S72_0:952-956_1011-1015	1S72_0:2822-2829_2911-2914		
1S72_0:290-296_355-362	1S72_0:2845-2855_2903-2906		
1S72_0:380-384_405-408	1S72_0:43-50_111-113		
1S72_0:451-467_474-479	1S72_0:2672-2676_2809-2817		
1S72_9:75-81_101-106			
**E-loop cluster**	**C-loop cluster**	**reverse Kink-turn cluster**	
1J5E A:580-584 757-761	1J5E_A:371-375_389-390	1S72_0:1122-1134_1228-1242	
1J5E_A:779-783_799-803	1S72_0:2717-2721_2761-2763	1S72_0:1521-1529_1662-1665	
1S72_0:1542-1546_1639-1643	1S72_0:1425-1429_1437-1439	1S72_0:1531-1534_1657-1660	
1S72_0:705-709_719-723	1S72_0:958-963_1005-1008		
1S72_0:794-798_815-819			

Table S3: Motif groups from RNA 3D Motif Atlas release 3.93 for known motifs collected from rRNA of 1S72 and 1J5E

GNRA group		
1J5E_A:1076-1081	1S72_0:2695-2700	1S72_0:1862-1867
1J5E_A:296-301	1S72_0:468-473	1S72_0:252-257
1J5E_A:1265-1270	1S72_0:493-499	1S72_0:2876-2881
1J5E_A:158-163	1S72_0:576-581	1S72_0:733-738
1J5E_A:379-384	1S72_0:1705-1713	1S72_0:690-695
1J5E_A:458-474	1S72_0:1054-1060	1S72_0:1793-1799
1J5E_A:726-731	1S72_0:2248-2253	1S72_0:2629-2634
1J5E_A:897-902	1S72_0:1326-1331	1S72_9:89-94
1J5E_A:1012-1017	1S72_0:1468-1474	
1J5E_A:1515-1520	1S72_0:1628-1633	
**Sarcin-ricin group**	**Kink-turn group**	**Tandem-shear* group**
1J5E_A:887-894_905-910	1J5E _A:683-688_699-707	1J5E_A:1416-1419_1481-1484
1J5E_A:446-450_484-488	1J5E _A:242-247_277-284	1J5E_A:1259-1262_1273-1276
1S72_0:158-164_171-178	1S72 _0:245-249_260-266	1S72_0:794-798 _815-819
1S72_0:2689-2695_2700-2705	1S72_0:936-940 _1026-1034	1S72_0:2873-2876_2881-2884
1S72_0:210-216_224-229	1S72_0:77-81_93-100	1S72_0:2501-2505 _2515-2519
1S72_0:355-362_290-296	1S72 _0:1587-1592_1602-1608	1S72_0:516-522_21-27
1S72_0:1367-1373_2052-2057	1S72 _0:1312-1319_1338-1342	
1S72_0:952-956_1011-1015	1S72_0:1147-1155_1212-1216	
1S72_0:585-591_567-572		
1S72_9:75-81_101-106		
**Hook-turn* group**	**E-loop* group**	**C-loop group**
1S72_0:1456-1460_1483-1489	1J5E A:580-584 757-761	1J5E_A:371-375_389-390
1S72_0:2772-2777_2797-2802	1J5E_A:779-783_799-803	1J5E_A:292-293_304-308
1S72_0:1095-1099_1257-1261	1S72_0:1542-1546_1639-1643	1S72_0:2717-2721_2761-2763
	1S72_0:705-709_719-723	1S72_0:1425-1429_1437-1439
	1S72_0:2672-2676_2809-2817	

*In RNA 3D Motif Atlas, Tandem-shear motifs are annotated as Triple/Double sheared, Hook-turn motifs are annotated as UAA/GAN and E-loop motifs are annotated as tSH-tHW-tHS.

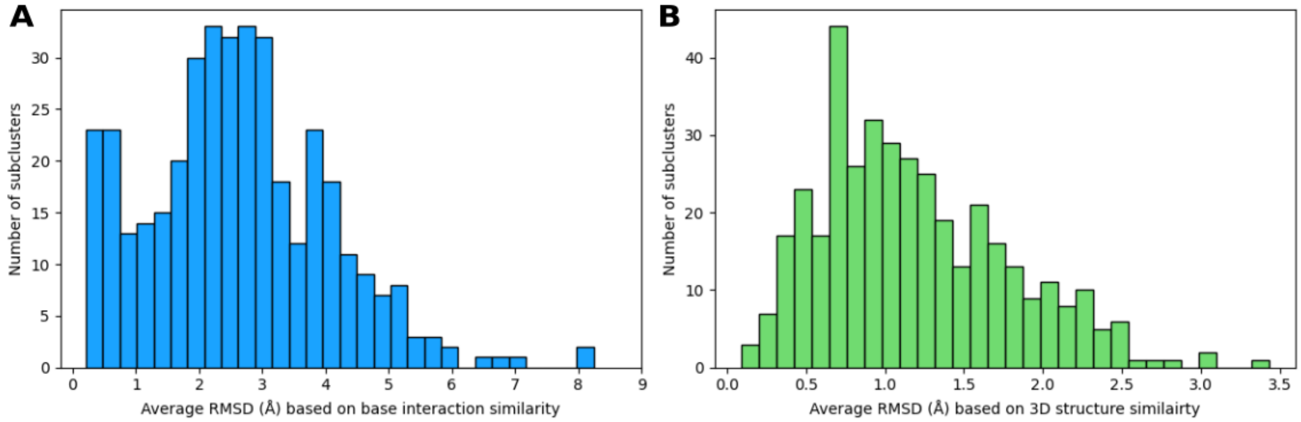

Figure S2: Distribution of number of internal loop subclusters generated by GINClus for average RMSD (Å) based on (A) interaction similarity and (B) 3D strucutre similarity.

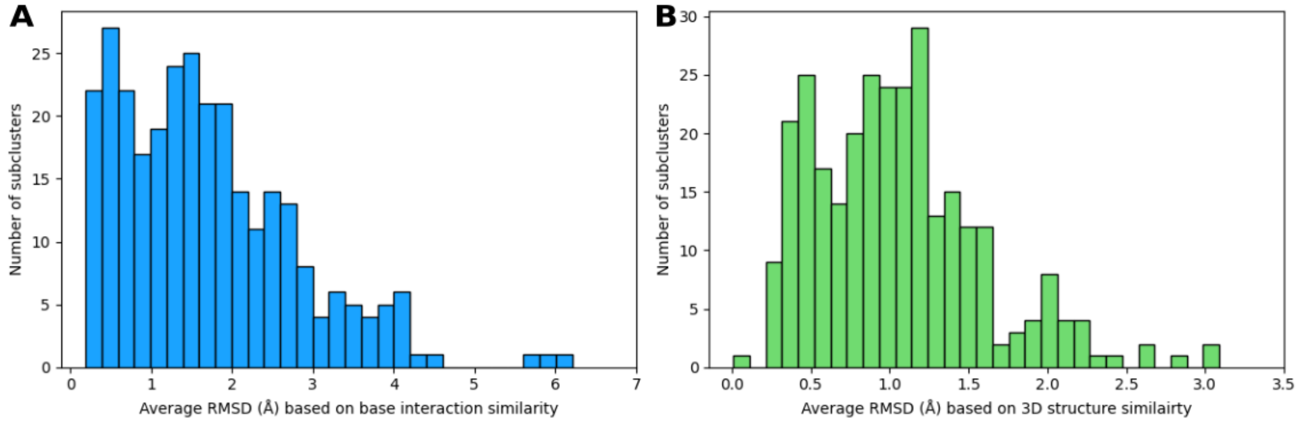

Figure S3: Distribution of number of hairpin loop subclusters generated by GINClus for average RMSD (Å) based on (A) interaction similarity and (B) 3D strucutre similarity.

Table S4: Statistics of RMSD values generated for known motif families

	RMSD	SR	KT	TS	HT	EL	CL	GNRA	TL	IL[a]	HL[b]
Base interaction similarity	Mean (Å)	1.83	2.30	1.36	1.52	1.64	2.40	0.63	1.00	1.84	0.82
	Maximum (Å)	7.77	8.23	6.04	8.06	6.14	7.83	4.75	4.18	8.23	4.75
	SD[c] (Å)	1.59	1.90	1.11	1.21	1.12	1.73	0.51	0.67	1.44	0.59
	Upper-bound[d] (Å)	5.01	6.10	3.58	3.94	3.88	5.86	1.65	2.34	4.72	2.00
3D structure similarity	Mean (Å)	1.28	1.55	1.05	1.17	1.41	1.10	0.59	0.87	1.26	0.73
	Maximum (Å)	3.78	4.15	2.99	2.84	3.18	3.25	2.61	2.94	4.15	2.94
	SD (Å)	0.73	0.76	0.47	0.51	0.53	0.59	0.37	0.45	0.60	0.41
	Upper-bound (Å)	2.74	3.07	1.99	2.19	2.47	2.28	1.33	1.77	2.46	1.55

[a]IL (internal loop) contains motifs from families SR, KT, TS, HT, EL and CL, [b]HL (hairpin loop) contains motifs from families GNRA and TL, [c]SD refers to standard deviation, [d]Upper-bound refers to upper-bound of range where range = mean $\pm 2 *$ standard deviation

Table S5: RNA motif subclusters belonging to Sarcin-ricin motif family

Subcluster ID	Motif location	Motif family	Avg. 3D structure-based RMSD/AL	Common base-pair interactions
16	5TBW_1:32-37_46-52	Known Sarcin-ricin	0.23/13	C-C tH/S, A-A tH/H, A-U tH/W, G-A tS/H
	4V91_1:32-37_46-52	New Sarcin-ricin		
	4V88_A6:144-149_165-171	Outlier		
27	4V8P_D1:830-834_956-963	Known Sarcin-ricin	1.49/10.14	A-G tH/W or tW/W, U-A tW/H or tS/H, A-A tH/H, G-U cS/H, A-G tH/S, U-A or G-A tS/H
	3J7Q_5:1522-1526_1648-1655	Known Sarcin-ricin		
	5TBW_1:1444-1450_2354-2359	Known Sarcin-ricin		
	6D9J_5:1522-1526_1648-1655	New Sarcin-ricin		
	6EK0_L5:2358-2364_3858-3863	New Sarcin-ricin		
	6EK0_L5:2867-2872_2877-2883	New Sarcin-ricin		
	3J7Q_5:2867-2872_2877-2883	New Sarcin-ricin		
	3J7Q_5:2358-2364_3858-3863	New Sarcin-ricin		
	5OOL_A:1906-1910_2009-2016	New Sarcin-ricin		
	5XXB_1:895-899_1021-1028	New Sarcin-ricin		
	6GAW_BA:242-246_346-353	New Sarcin-ricin		
	4V91_1:1444-1450_2354-2359	New Sarcin-ricin		
	6EK0_L5:1522-1526_1648-1655	New Sarcin-ricin		
	6D9J_5:2358-2364_3858-3863	New Sarcin-ricin		
	4V8P_D1:1942-1947_1952-1958	New Sarcin-ricin		
	4V91_1:1918-1923_1928-1934	New Sarcin-ricin		
	4V91_1:805-809_931-938	New Sarcin-ricin		
	6AZ3_1:856-860_982-989	New Sarcin-ricin		
	5TBW_1:805-809_931-938	New Sarcin-ricin		
	5T5H_A:972-976_1098-1105	New Sarcin-ricin		
	5TBW_1:1918-1923_1928-1934	New Sarcin-ricin		
	5XXB_1:1541-1547_2467-2472	New Sarcin-ricin		
	5TBW_1:1388-1395_1415-1419	Outlier		
	4LFB_A:684-688_699-706	Outlier		
	6CHR_A:429-434_491-497	Outlier		
	4V91_1:1388-1395_1415-1419	Outlier		
29	4LFB_A:887-894_905-910	Known Sarcin-ricin	0.91/11.62	A-G tH/S, U-A tW/H, G-U cS/H, A-A tH/H, G-A tS/H
	3IGI_A:296-303_316-321	Known Sarcin-ricin		
	5J7L_DA:239-245_253-258	Known Sarcin-ricin		
	6ERI_BA:837-844_855-860	New Sarcin-ricin		
	5NGM_Aa:896-903_914-919	New Sarcin-ricin		
	6AZ3_1:81-88_96-101	New Sarcin-ricin		
	6HA1_A:241-248_256-262	New Sarcin-ricin		
	5OPT_E:1579-1586_1597-1602	New Sarcin-ricin		
	6HA1_a:897-904_915-920	New Sarcin-ricin		
	6AZ1_1:1463-1470_1481-1486	New Sarcin-ricin		
	6ERI_AA:685-689_810-817	Outlier		
	6ERI_AA:1071-1079_1136-1140	Outlier		
	4IOA_X:715-721_736-743	Outlier		
34	4V8P_C3:72-78_98-103	Known Sarcin-ricin	1.22/9.79	A-G tH/S, U-A tW/H, G-U cS/H, A-A tH/H, C-C or U-C tS/H
	4IOA_X:165-170_179-185	Known Sarcin-ricin		
	4IOA_X:216-222_230-235	Known Sarcin-ricin		
	4Y4O_2A:188-193_202-208	Known Sarcin-ricin		
	5T5H_D:72-78_98-103	New Sarcin-ricin		
	6AZ3_8:74-80_100-105	New Sarcin-ricin		
	5XXB_1:593-598_612-618	Outlier		
	5O60_A:1136-1142_1258-1263	Outlier		
	5V7Q_A:1147-1153_1269-1274	Outlier		
63	4V88_A6:1111-1118_1129-1134	Known Sarcin-ricin	1.10/10.62	A-G tH/S, U-A tW/H, G-U cS/H, G-A tS/H
	3J7P_S2:1168-1175_1186-1191	Known Sarcin-ricin		
	5XXU_2:1106-1113_1124-1129	New Sarcin-ricin		
	4V88_A6:1111-1118_1129-1134	New Sarcin-ricin		
	6FYY_2:1110-1117_1128-1133	New Sarcin-ricin		
	4Y4O_2A:2636-2640_2774-2782	New Sarcin-ricin		
	4IOA_X:2615-2619_2754-2762	New Sarcin-ricin		
	4WF9_X:2663-2667_2801-2809	New Sarcin-ricin		
	6ERI_AA:2653-2657_2792-2800	New Sarcin-ricin		
	6HA1_A:2665-2669_2803-2811	New Sarcin-ricin		
	5XXB_1:1812-1818_1828-1834	Outlier		
70	5TBW_1:3332-3337_3367-3371	Known Sarcin-ricin	1.21/7.71	A-G tH/S, U-A cH/H or tH/H
	4V8P_D1:3290-3295_3323-3327	New Sarcin-ricin		
	4V91_1:3332-3337_3367-3371	New Sarcin-ricin		
	6ERI_AA:1224-1229_1259-1263	New Sarcin-ricin		
	6HA1_A:1756-1761_1772-1776	New Sarcin-ricin		
	6D9J_5:4632-4637_4662-4666	New Sarcin-ricin		
	5OQL_2:112-115_190-196	Outlier		
	3J7Q_5:4632-4637_4662-4666	Outlier		

Table S5: RNA motif subclusters belonging to Sarcin-ricin motif family (continued)

Subcluster ID	Motif location	Motif family	Avg. 3D structure-based RMSD/AL	Common base-pair interactions
89	6GAW_BA:73-79_87-92	Known Sarcin-ricin	0.53/13	A-G or A-A tH/S, U-A tW/H, G-U cS/H, A-A tH/H, A-A tS/H
	1NBS_B:138-143_165-171	Known Sarcin-ricin		
	5OOL_A:1739-1745_1753-1758	Known Sarcin-ricin		
95	6GAW_BA:641-647_1006-1011	Known Sarcin-ricin	0.50/13	A-G tH/S, U-A tW/H, G-U cS/H, A-A tH/H, G-A tS/H
	4IOA_X:1276-1282_1994-1999	Known Sarcin-ricin		
	5MRC_A:1296-1302_1911-1916	Known Sarcin-ricin		
	6ERI_AA:1284-1290_2025-2030	New Sarcin-ricin		
	5FLX_z:70-75_91-97	New Sarcin-ricin		
	4Y4O_2A:1263-1269_2011-2016	New Sarcin-ricin		
	4RGE_C:3-8_47-54	Outlier		
	3IGI_A:149-156_221-225	Outlier		
122	4IOA_X:2632-2637_2642-2646	Known Sarcin-ricin	0.59/9.73	A-G tH/S, U-A tW/H, G-U cS/H, A-C tH/H or tH/W
	4WF9_X:2680-2685_2690-2694	Known Sarcin-ricin		
	4Y4O_2A:2653-2658_2663-2667	Known Sarcin-ricin		
	5J7L_DA:2653-2658_2663-2667	Known Sarcin-ricin		
	5MRC_A:2920-2925_2930-2934	New Sarcin-ricin		
	5O60_A:2877-2882_2887-2891	New Sarcin-ricin		
	5V7Q_A:2891-2896_2901-2905	New Sarcin-ricin		
	6HA1_A:2682-2687_2692-2696	New Sarcin-ricin		
	6ERI_AA:2670-2675_2680-2684	New Sarcin-ricin		
	3NDB_M:192-198_223-226	New Sarcin-ricin		
	5XYI_2:1087-1091_1233-1238	Outlier		
134	3J79_A:1593-1599_2647-2652	Known Sarcin-ricin	1.50/11.15	A-A tW/W or tH/W, U-A tW/H, A-A or A-G tH/S, A-A tH/H, U-A tS/H
	4IOA_X:687-691_812-819	Known Sarcin-ricin		
	4WF9_X:719-723_844-851	Known Sarcin-ricin		
	4Y4O_2A:674-678_799-806	Known Sarcin-ricin		
	5J7L_DA:674-678_799-806	Known Sarcin-ricin		
	5XXB_1:2021-2026_2031-2037	New Sarcin-ricin		
	6HA1_A:721-725_846-853	New Sarcin-ricin		
	6AZ3_2:102-107_112-118	New Sarcin-ricin		
	5MRC_A:583-587_690-697	New Sarcin-ricin		
	4IOA_X:1703-1708_1713-1719	New Sarcin-ricin		
	5T5H_B:102-107_112-118	New Sarcin-ricin		
	5T2A_B:101-106_111-117	New Sarcin-ricin		
	3J79_A:2186-2191_2196-2202	New Sarcin-ricin		
	2YGH_A:17-21_31-38	Outlier (Kink-turn)		
151	3J79_B:72-78_98-103	Known Sarcin-ricin	1.44/13	A-G tH/S, A-A tH/H, tS/H
	5XY3_1:1583-1588_1593-1599	New Sarcin-ricin		
	5V7Q_A:356-365_439-441	Outlier		
167	4V9F_0:1367-1373_2052-2057	Known Sarcin-ricin	0.78/13	A-G tH/S, U-A tW/H, G-U cS/H, A-A tH/H, U-C tS/H
	4V9F_0:210-216_224-229	Known Sarcin-ricin		
	5J7L_DA:188-193_202-208	Known Sarcin-ricin		
	4V9F_0:2689-2695_2700-2705	Known Sarcin-ricin		
	5XXB_1:3131-3137_3142-3147	New Sarcin-ricin		
	4V91_1:3019-3025_3030-3035	New Sarcin-ricin		
	5TBW_1:3019-3025_3030-3035	New Sarcin-ricin		
	6HA1_A:191-196_205-211	New Sarcin-ricin		
191	4Y4O_2A:2847-2852_2865-2869	Known Sarcin-ricin	0.47/11	A-G tH/S, U-A tH/H or cH/H
	5J7L_DA:2847-2852_2865-2869	Known Sarcin-ricin		
	5XY3_1:2693-2698_2728-2732	New Sarcin-ricin		
205	3J7O_7:71-78_98-104	Known Sarcin-ricin	0.76/15	U-A tW/H, U-U tS/H, A-A tH/H, G-U cS/H, U-A tW/H, A-G tH/S
	5TBW_1:82-89_97-103	Known Sarcin-ricin		
	6EK0_L7:71-78_98-104	New Sarcin-ricin		
	5XXB_3:71-78_98-104	New Sarcin-ricin		
	4V91_1:82-89_97-103	New Sarcin-ricin		
	6D9J_7:71-78_98-104	New Sarcin-ricin		
247	3K0J_E:59-63_76-80	Known Sarcin-ricin	0.58/10	G-G cW/H
	2GDI_X:59-63_76-80	Known Sarcin-ricin		
	5J7L_DA:1669-1674_1990-1993	Outlier		
249	4LCK_F:16-23_83-90	Known Sarcin-ricin	1.58/11.19	G-A tS/H, A-A or A-G tH/H, G-U cS/H, A-U or A-A tW/H, A-G tH/S
	4V5O_BA:1083-1090_1102-1107	Known Sarcin-ricin		
	3DIL_A:22-30_63-70	Known Sarcin-ricin		
	3J7A_A:1212-1219_1230-1235	Known Sarcin-ricin		
	3D0U_A:20-27_60-66	Known Sarcin-ricin		
	5J7L_AA:409-416_427-433	New Sarcin-ricin		
	6HA1_a:417-424_435-441	New Sarcin-ricin		
	5NGM_Aa:417-425_435-441	New Sarcin-ricin		
	4LFB_A:409-416_427-433	New Sarcin-ricin		
	5MRC_A:58-63_407-413	Outlier		

Table S5: RNA motif subclusters belonging to Sarcin-ricin motif family (continued)

Subcluster ID	Motif location	Motif family	Avg. 3D structure-based RMSD/AL	Common base-pair interactions
252	4V9F_0:2864-2869_2888-2892<br>3J79_A:3406-3411_3416-3420<br>5OQL_2:80-86_263-266	Known Sarcin-ricin<br>New Sarcin-ricin<br>Outlier	1.88/8.0	A-G tH/S
277	5TBW_AS:72-80_100-106<br>4V91_3:72-80_100-106<br>6HA1_A:1827-1833_1842-1850<br>2Z75_B:101-108_123-131<br>5T5H_A:421-424_435-448	Known Sarcin-ricin<br>New Sarcin-ricin<br>New Sarcin-ricin<br>Outlier<br>Outlier	1.59/12.67	A-G tH/S,<br>U-A or A-A tW/H,<br>G-U cS/H,<br>A-A tH/H
293	4V9F_0:765-769_892-899<br>6GAW_BA:1455-1461_1466-1471<br>4LFB_A:450-455_477-483	Known Sarcin-ricin<br>New Sarcin-ricin<br>New Sarcin-ricin	2.24/7.33	U-A or A-A tW/H
313	5J7L_AA:887-894_905-910<br>5MRC_aa:952-959_970-975<br>5V93_a:880-887_898-903<br>5ZEB_a:869-876_887-892<br>5OQL_2:87-95_258-262<br>6D9J_5:3714-3720_3733-3739	Known Sarcin-ricin<br>New Sarcin-ricin<br>New Sarcin-ricin<br>New Sarcin-ricin<br>Outlier<br>Outlier	0.50/14.0	A-A or A-G tH/S,<br>U-A tW/H,<br>G-U cS/H,<br>A-A tH/H, G-A tS/H
358	3J7Q_5:31-37_44-51<br>6D9J_5:31-37_44-51<br>4WF9_X:1451-1460_1628-1632	Known Sarcin-ricin<br>New Sarcin-ricin<br>Outlier	0.87/15	A-A tH/H,<br>A-U tH/W
362	3J79_A:3724-3729_3759-3763<br>5J7L_DA:1712-1718_1742-1746	Known Sarcin-ricin<br>Known Sarcin-ricin	1.21/6	A-G tH/S
392	5J7L_DA:1263-1269_2011-2016<br>5MRC_A:279-285_293-298<br>5OOL_A:2307-2313_2674-2679	Known Sarcin-ricin<br>Known Sarcin-ricin<br>Known Sarcin-ricin	0.67/13.0	A-G tH/S, U-A tW/H<br>G-U cS/H, A-A tH/H<br>A-A or G-A tS/H

For each subcluster, the average (avg.) 3D structure-based RMSD, alignment length (AL) and common base-pairs are generated after excluding the outliers.

Table S6: RNA motif subclusters belonging to Kink-turn motif family

Subcluster ID	Motif location	Motif family	Avg. 3D structure-based RMSD/AL	Common base-pair interactions
14	4LFB_A:515-521_528-536<br>5J7L_AA:515-521_528-536<br>6HA1_a:524-530_537-545<br>5V93_a:506-512_519-527<br>5ZEB_a:495-501_508-516<br>5XY3_1:1672-1677_1690-1697<br>5NGM_Aa:523-529_536-544	Known Kink-turn<br>Known Kink-turn<br>New Kink-turn<br>New Kink-turn<br>New Kink-turn<br>New Kink-turn<br>New Kink-turn	1.20/12.90	A-G tH/S, tW/W,<br>U-A tW/H,<br>U-C cS/W or cW/W
24	4V8P_D1:1414-1421_1441-1445<br>5Y7M_D:17-24_29-33<br>5NGM_Aa:692-696_707-714<br>5V7Q_A:803-807_928-935<br>5O60_A:789-793_914-921	Known Kink-turn<br>New Kink-turn<br>New Kink-turn<br>Outlier<br>Outlier	1.4/9.17	A-G tH/S,<br>G-A tS/H,<br>G-A tS/S
46	4IOA_X:1221-1228_1247-1251<br>2HW8_B:10-13_22-29<br>6ERI_AA:80-84_96-103<br>5V7Q_A:1339-1346_1365-1369<br>4Y1M_B:8-15_96-101	Known Kink-turn<br>New Kink-turn<br>New Kink-turn<br>New Kink-turn<br>Outlier	1.32/9.83	A-G tH/S,<br>A-G tH/S,<br>G-A tS/H, G-A tS/S
51	4V5O_BA:556-562_569-577<br>4V88_A6:562-568_575-583<br>3J7A_A:569-575_582-590<br>5XXU_2:561-567_574-582<br>3J7P_S2:611-617_624-632<br>5XYI_2:484-490_497-505<br>6D9J_2:611-617_624-632<br>6AZ1_1:611-617_624-632<br>5OPT_E:616-622_629-637<br>6FYY_2:561-567_574-582<br>6EK0_S2:611-617_624-632<br>6FRK_1:107-111_239-247	Known Kink-turn<br>Known Kink-turn<br>Known Kink-turn<br>New Kink-turn<br>New Kink-turn<br>New Kink-turn<br>New Kink-turn<br>New Kink-turn<br>New Kink-turn<br>New Kink-turn<br>New Kink-turn<br>Outlier	0.62/15.82	A-G tH/S,<br>A-A tW/W,<br>U-C cS/S,<br>U-A tW/H

Table S6: RNA motif subclusters belonging to Kink-turn motif family (continued)

Subcluster ID	Motif location	Motif family	Avg. 3D structure-based RMSD/AL	Common base-pair interactions
54	5J7L_AA:684-688_699-706	Known Kink-turn	2.0/10.2	A-G tW/S, A-G tH/S, G-A tS/S, G-G or G-A tS/H
	4IOA_X:1426-1430_1598-1605	New Kink-turn		
	6HA1_a:693-697_708-715	New Kink-turn		
	5MRC_aa:689-695_821-826	New Kink-turn		
	6HA1_A:2158-2165_2184-2188	New Kink-turn		
65	3J7Q_5:2302-2309_2329-2333	Known Kink-turn	0.80/13.0	A-G tH/S, A-G tH/S, G-A tS/H
	6EK0_L5:2302-2309_2329-2333	New Kink-turn		
	6D9J_5:2302-2309_2329-2333	New Kink-turn		
	5T5H_A:248-249_253-263	Outlier		
	6AZ3_1:244-245_249-259	Outlier		
74	3J7A_A:317-322_354-361	Known Kink-turn	1.75/8.2	G-A or G-G tS/S, C-A cS/S, A-G tH/S
	5AN9_N:2423-2428_2439-2446	New Kink-turn		
	5MRC_aa:246-251_282-289	New Kink-turn		
	5XYI_2:715-719_730-737	New Kink-turn		
	4V88_A6:1488-1494_1513-1518	New Kink-turn		
83	5TBW_1:2156-2161_2173-2180	Known Kink-turn	1.40/8.19	G-G tW/W, A-U tW/W, G-G or A-U tS/S, A-G tH/S, G-U tS/H
	4WF9_X:81-85_97-104	Known Kink-turn		
	6HA1_A:81-85_97-104	New Kink-turn		
	5O60_A:78-82_95-102	New Kink-turn		
	5XXB_1:2270-2275_2286-2293	New Kink-turn		
	4V91_1:2156-2161_2173-2180	New Kink-turn		
	6EK0_L5:2482-2487_2491-2496	Outlier		
87	4LCK_F:6-12_94-97	Known Kink-turn	1.05/8.86	A-G tH/S, G-A tS/H, G-A tS/S or tS/W
	5XTM_D:16-23_30-33	New Kink-turn		
	5DCV_D:18-25_32-35	New Kink-turn		
	5GAN_V:28-34_43-46	New Kink-turn		
	5GAP_V:28-34_43-46	New Kink-turn		
	5XTM_B:16-23_30-33	New Kink-turn		
	6D9J_2:563-565_585-592	Outlier		
	5OPT_E:564-566_590-597	Outlier		
	4OJI_A:5-9_44-50	Outlier		
	5NGM_Aa:454-458_491-496	Outlier		
121	4KQY_A:17-21_31-38	Known Kink-turn	0.66/11.0	A-G tH/S, G-A tS/H, A-A or G-A tS/H or tS/W
	5XY3_1:1385-1391_1401-1407	New Kink-turn		
161	4V9F_0:937-940_1026-1033	Known Kink-turn	0.92/10.2	tS/S, A-G tH/S, G-A tS/H
	3NMU_E:14-17_24-30	Known Kink-turn		
	3NVI_F:5-8_15-21	Known Kink-turn		
	1RLG_D:5-8_15-21	Known Kink-turn		
	3NVK_K:6-9_16-22	New Kink-turn		
	1OOA_C:5-10_21-25	Outlier		
	5J7L_DA:2129-2136_2155-2159	Outlier		
182	4V9F_0:77-81_93-100	Known Kink-turn	1.06/10.9	A-G tS/S or tW/S, A-G tH/S, G-A tS/H, G-A tS/H
	5FJ4_H:3-7_26-33	Known Kink-turn		
	5FJC_A:17-21_31-38	Known Kink-turn		
	4BW0_A:3-7_16-23	Known Kink-turn		
	4CS1_A:3-10_13-17	New Kink-turn		
230	3J7Q_5:2656-2662_2672-2678	Known Kink-turn	0.80/14.0	A-C tS/S, A-G tH/S, G-A tS/H, G-A tS/H
	3J79_A:1868-1874_1884-1890	Known Kink-turn		
	6EK0_L5:2656-2662_2672-2678	New Kink-turn		
	6D9J_5:2656-2662_2672-2678	New Kink-turn		
233	5TBW_1:1711-1717_1727-1733	Known Kink-turn	1.31/12.4	A-G tS/S, A-G tH/S, G-A tS/H, G-A or G-G tS/H
	4V9F_0:1312-1319_1338-1342	Known Kink-turn		
	4Y4O_2A:1208-1215_1234-1238	Known Kink-turn		
	4GMA_Z:93-97_143-150	Known Kink-turn		
	6ERI_BA:633-637_648-655	New Kink-turn		
	4V91_1:1711-1717_1727-1733	New Kink-turn		
	3D2V_A:10-13_26-36	Outlier (T-loop)		
	5J7L_DA:2636-2640_2774-2782	Outlier		
239	4V9F_0:1587-1592_1602-1608	Known Kink-turn	0.61/11.0	A-C tS/S or G-A tS/W, A-G tH/S, G-A tS/H, G-A tS/H
	6ERI_AA:1229-1236_1255-1259	New Kink-turn		
242	4IOA_X:1789-1795_1805-1813	Known Kink-turn	0.4/16.0	A-C tS/S or G-A tS/W, A-G tH/S, G-A tS/H, G-A tS/H
	6ERI_AA:1808-1814_1823-1831	New Kink-turn		
244	5J7L_DA:1043-1050_1109-1112	Known Kink-turn	0.75/12.0	G-A or C-A tS/H, G-A tW/H
	4GXY_A:82-86_139-146	Known Kink-turn		
	4WF9_X:1087-1094_1153-1156	New Kink-turn		
	3KTW_C:201-206_217-222	Outlier		

Table S6: RNA motif subclusters belonging to Kink-turn motif family (continued)

Subcluster ID	Motif location	Motif family	Avg. 3D structure-based RMSD/AL	Common base-pair interactions
248	4WF9_X:1246-1253_1272-1276	Known Kink-turn	1.05/13.0	A-G tH/S, G-A tS/H, G-A tS/H,
	6HA1_A:1248-1255_1274-1278	New Kink-turn		
	5O60_A:2352-2359_2378-2382	New Kink-turn		
276	3J7A_A:963-968_979-987	Known Kink-turn	1.44/7.67	A-G tS/S, A-G tH/S
	6AZ3_3:105-114_124-129	New Kink-turn		
	4V9F_0:1975-1986_2002-2005	Outlier		
279	5J7L_DA:1798-1804_1813-1821	Known Kink-turn	2.02/11.33	A-C tS/S, A-G tH/S, G-A tS/H or tW/H, C-C tW/H
	5TBW_1:1218-1226_1283-1287	Known Kink-turn		
	4V91_1:1218-1226_1283-1287	New Kink-turn		
300	4V8P_D1:1245-1252_1311-1314	Known Kink-turn	1.87/12.0	G-A tS/H
	5D8H_A:1153-1160_1219-1222	New Kink-turn		
	6EK0_L5:732-734_929-937	Outlier		
306	4V88_A6:311-316_348-355	Known Kink-turn	0.36/14.0	A-U tS/S, A-A tH/S, C-C cW/W, A-A tW/H
	6FYY_2:310-315_347-354	New Kink-turn		
	6AZ3_1:30-35_44-50	Outlier		
310	3J79_A:2449-2455_2466-2473	Known Kink-turn	0.37/13.0	A-A tW/W, C-A tW/H,
	5V93_a:1239-1245_1276-1282	New Kink-turn		
	5ZEB_a:1228-1234_1265-1272	Outlier		
327	5J7L_DA:1208-1215_1234-1238	Known Kink-turn	1.35/9.2	G-G tW/W, A-U tW/W, G-G tS/S, A-G tH/S, G-A tS/H, G-A tS/H
	3J7Q_5:3660-3665_3677-3684	Known Kink-turn		
	3U4M_B:2129-2136_2155-2159	Known Kink-turn		
	6EK0_L5:3660-3665_3677-3684	New Kink-turn		
	6D9J_5:3660-3665_3677-3684	New Kink-turn		
366	4V88_A6:895-899_910-917	Known Kink-turn	0.48/13.0	G-A tS/S, A-G tH/S, C-A tW/H
	6FYY_2:894-898_909-916	Known Kink-turn		
367	4V9F_0:1854-1859_1870-1877	Known Kink-turn	2.01/7.7	tH/S, cS/S, tW/H
	5XYI_2:243-248_278-285	New Kink-turn		
	6HIW_CA:230-236_243-251	New Kink-turn		
	6EK0_L5:466-471_683-689	New Kink-turn		
	3J7Q_5:466-472_682-688	New Kink-turn		
	5XXB_1:3291-3299_3309-3314	Outlier		
390	5J7L_AA:242-247_277-284	Known Kink-turn	1.57/8.68	G-A tS/S, A-G tH/S, A-A tW/H, C-A cS/S
	6HA1_a:250-255_285-292	New Kink-turn		
	4P8Z_A:72-75_86-94	New Kink-turn		
	4P95_A:290-293_304-312	New Kink-turn		
	6EK0_S2:952-956_967-974	New Kink-turn		
	5XXU_2:311-316_348-355	New Kink-turn		
	3J7P_S2:952-956_967-974	New Kink-turn		
	6D9J_5:1926-1933_2053-2058	Outlier		
	4LVW_A:5-9_77-84	Outlier		
	4NLF_A:2652-2658_2663-2668	Outlier		

For each subcluster, the average (avg.) 3D structure-based RMSD, alignment length (AL) and common base-pairs are generated after excluding the outliers.

Table S7: RNA motif subclusters belonging to Tandem-shear motif family

Subcluster ID	Motif location	Motif family	Avg. 3D structure-based RMSD/AL	Common base-pair interactions
17	4WF9_X:1461-1465_1624-1627	Known Tandem-shear	0.93/5.43	A-G tH/S, U-A tW/H
	6D9J_2:189-193_207-210	New Tandem-shear		
	3AGV_S:4-9_18-20	New Tandem-shear		
	5O60_A:1638-1642_1797-1800	New Tandem-shear		
	6ERI_AA:1458-1461_1585-1589	New Tandem-shear		
	4V5O_BA:354-356_367-372	New Tandem-shear		
	5XY3_1:2508-2511_2535-2539	New Tandem-shear		
	5OPT_E:1678-1681_1965-1969	Outlier		
	6ERI_BA:49-54_331-333	Outlier		
	4V5O_BA:354-356_367-372	Outlier		

Table S7: RNA motif subclusters belonging to Tandem-shear motif family (continued)

Subcluster ID	Motif location	Motif family	Avg. 3D structure-based RMSD/AL	Common base-pair interactions
21	4V88_A6:186-189_196-199	Known Tandem-shear	1.11/6.64	A-G or A-A tH/S, G-A or A-A tS/H
	4V8P_D1:162-165_254-257	Known Tandem-shear		
	6ERI_AA:2367-2370_2381-2384	New Tandem-shear		
	4V9F_0:2331-2333_2351-2355	New Tandem-shear		
	6ERI_AA:1487-1490_1554-1557	New Tandem-shear		
	6ERI_AA:1866-1869_1897-1900	New Tandem-shear		
	5V7Q_A:2588-2591_2602-2605	New Tandem-shear		
	4GXY_A:105-107_122-126	New Tandem-shear		
	6FYY_2:185-188_195-198	New Tandem-shear		
	5OQL_1:88-91_116-119	New Tandem-shear		
	5XYM_B:24-29_60-61	Outlier		
	6DZP_B:24-29_60-61	Outlier		
	3P49_A:109-113_142-144	Outlier		
	3OXE_A:31-35_70-72	Outlier		
	5WTI_B:1-5_25-27	Outlier		
	6DZI_B:24-29_60-61	Outlier		
31	4V8P_D1:2824-2827_2837-2840	Known Tandem-shear	0.76/7.64	A-G tH/S, A-A tS/H
	5J7L_DA:2467-2470_2480-2483	Known Tandem-shear		
	4V9F_0:2502-2505_2515-2518	Known Tandem-shear		
	4WF9_X:2494-2497_2507-2510	New Tandem-shear		
	6ERI_AA:2484-2487_2497-2500	New Tandem-shear		
	5O60_A:2691-2694_2704-2707	New Tandem-shear		
	6AZ3_2:1275-1278_1288-1291	New Tandem-shear		
	5J7L_AA:1259-1262_1273-1276	New Tandem-shear		
	5V7Q_A:2705-2708_2718-2721	New Tandem-shear		
	6HA1_A:2496-2499_2509-2512	New Tandem-shear		
	6EK0_L5:4413-4416_4426-4429	New Tandem-shear		
	3J7P_S2:1453-1455_1471-1475	Outlier		
	6ERI_AA:1340-1344_1352-1354	Outlier		
38	4V88_A6:1653-1656_1744-1747	Known Tandem-shear	0.98/7.87	A-G tH/S or tW/S, G-A tS/H or tS/W
	4V8P_D1:3300-3303_3316-3319	Known Tandem-shear		
	5AOX_C:58-61_105-108	Known Tandem-shear		
	5BTP_B:12-15_41-44	Known Tandem-shear		
	3J7P_S2:1733-1736_1798-1801	Known Tandem-shear		
	3J7P_S2:1717-1720_1813-1816	New Tandem-shear		
	5OPT_E:2187-2190_2262-2265	New Tandem-shear		
	5XXB_1:850-853_859-862	New Tandem-shear		
	5XYI_2:1459-1462_1524-1527	New Tandem-shear		
	5XXU_2:1651-1654_1735-1738	New Tandem-shear		
	6D9J_2:1733-1736_1798-1801	New Tandem-shear		
	6AZ1_1:2073-2076_2147-2150	New Tandem-shear		
	6D9J_2:1717-1720_1813-1816	New Tandem-shear		
	6FYY_2:1651-1654_1742-1745	New Tandem-shear		
	6EK0_S2:1717-1720_1813-1816	New Tandem-shear		
	6EK0_S2:1733-1736_1798-1801	New Tandem-shear		
	4V91_1:3342-3345_3360-3363	New Tandem-shear		
	6EK0_L5:5014-5017_5032-5035	New Tandem-shear		
	3J7Q_5:5014-5017_5032-5035	New Tandem-shear		
	5XXB_1:3425-3428_3441-3444	New Tandem-shear		
	5TBW_1:3342-3345_3360-3363	New Tandem-shear		
	6D9J_5:5014-5017_5032-5035	New Tandem-shear		
	3IGI_A:158-161_216-219	Outlier		
59	4V9F_0:2873-2876_2881-2884	Known Tandem-shear	0.99/7.41	A-G tH/S, G-A tS/H
	4WF9_X:2377-2380_2391-2394	Known Tandem-shear		
	4Y4O_2A:2350-2353_2364-2367	Known Tandem-shear		
	4ZNP_A:9-12_37-40	Known Tandem-shear		
	5J7L_DA:2350-2353_2364-2367	Known Tandem-shear		
	3D0U_A:16-19_67-70	Known Tandem-shear		
	3DIL_A:19-22_70-73	Known Tandem-shear		
	3J7Q_5:1431-1434_1450-1453	Known Tandem-shear		
	4IOA_X:2329-2332_2343-2346	Known Tandem-shear		
	5O60_A:2574-2577_2588-2591	New Tandem-shear		
	6HA1_A:2379-2382_2393-2396	New Tandem-shear		
	6EK0_L5:1431-1434_1450-1453	New Tandem-shear		
	5XY3_1:2703-2706_2721-2724	New Tandem-shear		
	6D9J_5:1431-1434_1450-1453	New Tandem-shear		
	5OQL_1:454-457_475-478	New Tandem-shear		
	4IOA_X:1332-1336_1344-1346	Outlier (C-loop)		
	6HA1_A:1358-1362_1370-1372	Outlier		
	4WF9_X:1356-1360_1368-1370	Outlier		

Table S7: RNA motif subclusters belonging to Tandem-shear motif family (continued)

Subcluster ID	Motif location	Motif family	Avg. 3D structure-based RMSD/AL	Common base-pair interactions
84	4V8P_D1:125-128_138-141	Known Tandem-shear	1.21/7.61	A-A or A-G tH/S, A-A or G-A tS/H
	5ZEB_a:1414-1418_1450-1453	New Tandem-shear		
	6HA1_a:1440-1444_1476-1479	New Tandem-shear		
	5NGM_Aa:1441-1445_1477-1480	New Tandem-shear		
	4R4V_A:619-623_637-640	New Tandem-shear		
	6GAZ_AA:860-863_907-910	New Tandem-shear		
	6ERI_BA:1381-1385_1416-1419	New Tandem-shear		
	6HA1_A:1696-1701_2033-2035	New Tandem-shear		
	5J7L_DA:1651-1656_2004-2006	Outlier		
	4Y4O_2A:1651-1656_2004-2006	Outlier		
	4IOA_X:1668-1673_1987-1989	Outlier		
	5ZEB_a:440-442_472-477	Outlier		
165	4V8P_D1:3043-3048_3073-3077	Known Tandem-shear	1.30/11.0	A-G tH/S, A-A tW/H
	6FYY_2:64-69_82-86	New Tandem-shear		
	4V88_A6:64-69_82-86	New Tandem-shear		
178	4XWF_A:7-10_36-39	Known Tandem-shear	1.12/6.91	A-G tH/S, G-A tS/H
	5J7L_AA:1416-1419_1481-1484	Known Tandem-shear		
	5J7L_DA:536-539_554-557	Known Tandem-shear		
	4LFB_A:1416-1419_1481-1484	Known Tandem-shear		
	6ERI_AA:547-550_564-567	New Tandem-shear		
	3IGI_A:267-270_283-286	New Tandem-shear		
	5J7L_DA:2807-2811_2889-2891	New Tandem-shear		
	3J7P_S2:189-192_207-210	New Tandem-shear		
	6ERI_BA:1208-1211_1222-1225	New Tandem-shear		
	6ERI_BA:1366-1369_1431-1434	New Tandem-shear		
	6EK0_S2:189-192_207-210	New Tandem-shear		
	6AZ1_1:1966-1969_1980-1983	Outlier		
186	4WF9_X:581-584_597-600	Known Tandem-shear	0.99/8.0	A-G tH/S, G-A tS/H
	6HA1_a:1425-1428_1491-1494	New Tandem-shear		
	5NGM_Aa:1426-1429_1492-1495	New Tandem-shear		
	5O60_A:623-626_646-649	New Tandem-shear		
	6HA1_A:582-585_598-601	New Tandem-shear		
	5XXB_1:1986-1991_2460-2462	Outlier		
	4V5O_BA:1197-1202_1229-1230	Outlier		
240	4V5O_BA:1640-1643_1682-1685	Known Tandem-shear	0.35/8.0	A-A tH/S, G-A tS/H
	3J7A_A:1966-1969_2021-2024	Known Tandem-shear		
	5XYI_2:1475-1478_1509-1512	New Tandem-shear		
	5XXU_2:1667-1670_1720-1723	New Tandem-shear		
	5MRC_A:735-738_858-860	Outlier		
	5O60_A:374-376_425-429	Outlier		
264	4V8P_D1:2993-2996_3128-3133	Known Tandem-shear	0.65/7.0	A-G or A-A tH/s, G-A tS/H
	5OPT_E:2211-2215_2237-2241	New Tandem-shear		
	6AZ1_1:2097-2101_2122-2126	New Tandem-shear		
273	4LFB_A:1431-1435_1466-1469	Known Tandem-shear	0.63/9.0	A-G tH/S, G-A tS/H
	6HA1_A:1573-1576_1590-1594	New Tandem-shear		
312	4V8P_D1:571-574_594-598	Known Tandem-shear	1.34/8.6	A-G or A-A tH/S, A-C or A-A tS/H, A-C or A-A tS/H
	2R8S_R:112-116_205-208	Known Tandem-shear		
	4V5O_BA:37-40_458-462	New Tandem-shear		
	5XXU_2:38-41_466-470	New Tandem-shear		
	6D3P_A:6-9_32-36	New Tandem-shear		
	5XY3_1:725-729_772-775	Outlier		
383	5TBW_1:725-728_741-745	Known Tandem-shear	0.34/9.0	G-G tH/S, G-A tS/H
	4V91_1:725-728_741-745	New Tandem-shear		

For each subcluster, the average (avg.) 3D structure-based RMSD, alignment length (AL) and common base-pairs are generated after excluding the outliers.

Table S8: RNA motif subclusters belonging to Hook-turn motif family

Subcluster ID	Motif location	Motif family	Avg. 3D structure-based RMSD/AL	Common base-pair interactions
9	5J7L_DA:1417-1421_1577-1581	Known Hook-turn		
	4WF9_X:1042-1046_1197-1201	Known Hook-turn		
	4Y4O_2A:998-1002_1153-1157	Known Hook-turn		
	4Y4O_2A:1351-1355_1376-1380	Known Hook-turn		
	4IOA_X:1009-1013_1164-1168	Known Hook-turn		
	5J7L_DA:998-1002_1153-1157	Known Hook-turn		
	3J9M_AA:1388-1392_1414-1418	New Hook-turn		
	3GS5_A:4-8_19-23	New Hook-turn		
	5V7Q_A:1579-1583_1621-1625	New Hook-turn		
	5T83_A:12-15_42-47	New Hook-turn		
	4Y4O_2A:1851-1855_1887-1891	New Hook-turn	1.44/9.32	

Table S8: RNA motif subclusters belonging to Hook-turn motif family (continued)

Subcluster ID	Motif location	Motif family	Avg. 3D structure-based RMSD/AL	Common base-pair interactions
9	6ERI_AA:1861-1865_1901-19051	New Hook-turn	1.44/9.32	A-G tH/S, U-A tW/H
	2XXA_F:40-44_55-59	New Hook-turn		
	6HA1_A:1044-1048_1199-1203	New Hook-turn		
	5O60_A:1116-1120_1272-1276	New Hook-turn		
	3J79_A:3440-3444_3469-3473	New Hook-turn		
	6ERI_AA:1026-1030_1180-1184	New Hook-turn		
	5V7Q_A:1127-1131_1283-1287	New Hook-turn		
	4V9F_0:952-956_1011-1015	Outlier (C-loop)		
	5J7L_AA:779-783_799-803	Outlier (E-loop)		
	4LFB_A:779-783_799-803	Outlier (E-loop)		
	4V5O_BA:968-972_988-992	Outlier (E-loop)		
	6AZ1_1:599-606_636-637	Outlier		
	5OPT_E:604-611_641-642	Outlier		
	5NGM_Aa:787-791_807-811	Outlier		
32	4V8P_D1:3092-3096_3116-3120	Known Hook-turn	0.67/9.43	A-G tH/S, U-A tW/H
	3J7Q_5:4681-4685_4705-4709	Known Hook-turn		
	5TBW_1:3103-3107_3127-3131	Known Hook-turn		
	4V91_1:3103-3107_3127-3131	New Hook-turn		
	6EK0_L5:4681-4685_4705-4709	New Hook-turn		
	5XXB_1:3215-3219_3239-3243	New Hook-turn		
	5TBW_1:1167-1170_1329-1332	New Hook-turn		
	6BK8_i:622-624_1004-1007	Outlier		
	6ELZ_6:30-34_45-48	Outlier		
43	4V8P_D1:1194-1198_1355-1359	Known Hook-turn	0.97/9.56	A-G tH/S, U-A tW/H
	3J79_A:1295-1299_1456-1460	Known Hook-turn		
	5J7L_DA:2738-2742_2762-2766	Known Hook-turn		
	5OOL_A:2149-2153_2248-2252	New Hook-turn		
	5XY3_1:1245-1249_1262-1267	New Hook-turn		
	5O60_A:2962-2966_2986-2990	New Hook-turn		
	5V7Q_A:2976-2980_3000-3004	New Hook-turn		
	5OQL_1:651-656_668-672	New Hook-turn		
	6ERI_AA:2756-2760_2780-2784	New Hook-turn		
	4V8P_D1:563-568_601-604	Outlier		
140	4V9F_0:2724-2728_2754-2758	Known Hook-turn	1.20/9.6	A-G tH/S, G-A tS/H
	4Y4O_2A:1469-1473_1518-1523	Known Hook-turn		
	6ERI_AA:2704-2708_2736-2740	New Hook-turn		
	4WF9_X:2714-2718_2745-2749	New Hook-turn		
	6HA1_A:2716-2720_2747-2751	New Hook-turn		
	5NGM_Aa:588-592_765-769	New Hook-turn		
	5OPT_E:2079-2083_2094-2098	Outlier		
	4V91_1:759-763_768-772	Outlier		
	5TBW_1:759-763_768-772	Outlier		
148	4IOA_X:1431-1435_1593-1597	Known Hook-turn	1.56/7.73	A-U tH/W or A-G tH/S, G-A tS/H or U-A tW/H
	4Y4O_2A:280-284_357-360	New Hook-turn		
	3J9M_AA:1500-1504_1546-1549	New Hook-turn		
	5MRC_A:1603-1608_1904-1906	New Hook-turn		
	5O60_A:1748-1751_1762-1766	New Hook-turn		
	5XXB_1:1254-1258_1415-1419	New Hook-turn		
	3JAN_4:117-120_230-234	Outlier		
	4V5O_BA:41-47_423-425	Outlier		
204	4IOA_X:2718-2722_2742-2746	Known Hook-turn	0.30/6.5	A-G tH/S, U-A tW/H
	4V9F_0:2773-2777_2797-2801	Known Hook-turn		
	4Y4O_2A:2738-2742_2762-2766	Known Hook-turn		
	5V7Q_A:2090-2094_2120-2124	New Hook-turn		
	4V5O_BA:1649-1653_1672-1676	Outlier (E-loop)		
	5O60_A:2073-2077_2106-2110	Outlier		
220	5TBW_1:1532-1536_1585-1590	Known Hook-turn	1.13/10.83	A-G tH/S, U-A tW/H
	4V91_1:1532-1536_1585-1590	New Hook-turn		
	3OXE_A:38-42_61-66	New Hook-turn		
	5XXB_1:1629-1633_1679-1684	New Hook-turn		
229	4V8P_D1:1558-1562_1610-1615	Known Hook-turn	1.08/8.52	A-A or G-G tH/S, U-A or A-A tW/H
	3J79_A:1681-1685_1728-1733	Known Hook-turn		
	3J7P_S2:63-68_81-85	New Hook-turn		
	6D9J_2:63-68_81-85	New Hook-turn		
	6AZ1_1:64-69_80-84	New Hook-turn		
	5OPT_E:64-69_79-83	New Hook-turn		
	6EK0_S2:63-68_81-85	New Hook-turn		
	4FRN_B:6-14_75-76	Outlier		

Table S8: RNA motif subclusters belonging to Hook-turn motif family (continued)

Subcluster ID	Motif location	Motif family	Avg. 3D structure-based RMSD/AL	Common base-pair interactions
235	4V5O_BA:63-67_78-82<br>6HA1_A:922-926_945-949<br>6AZ3_7:122-126_137-141<br>5XY3_1:891-895_1050-1054<br>2IL9_A:6102-6106_6134-6138	Known Hook-turn<br>New Hook-turn<br>New Hook-turn<br>New Hook-turn<br>Outlier	1.73/9.67	A-G tH/S,<br>U-A tW/H
339	5MRC_A:3074-3078_3099-3103<br>5MRC_A:924-928_1173-1177<br>5AN9_N:3439-3443_3463-3467<br>5XY3_1:2554-2558_2579-2583<br>3J7A_A:64-68_84-88	Known Hook-turn<br>Known Hook-turn<br>New Hook-turn<br>New Hook-turn<br>New Hook-turn	1.25/9.9	A-G tH/S,<br>U-A tW/H
382	4V9F_0:2672-2676_2809-2817<br>3IGI_A:76-79_103-111	Known Hook-turn<br>New Hook-turn	2.22/10.0	A-G tH/S

For each subcluster, the average (avg.) 3D structure-based RMSD, alignment length (AL) and common base-pairs are generated after excluding the outliers.

Table S9: RNA motif subclusters belonging to E-loop motif family

Subcluster ID	Motif location	Motif family	Avg. 3D structure-based RMSD/AL	Common base-pair interactions
5	4YAZ_R:16-20_35-39<br>4V88_A6:990-994_1010-1014<br>4V9F_0:663-667_679-683<br>5XXU_2:987-991_1007-1011<br>6D9J_2:1047-1051_1067-1071<br>5V93_a:770-774_790-794<br>6HA1_a:788-792_808-812<br>6EK0_S2:1047-1051_1067-1071<br>5GAH_1:45-49_60-64<br>3LQX_B:146-150_161-165<br>5U3G_B:6-9_30-35<br>5ZEB_a:759-763_779-783<br>1DUH_A:45-49_60-64<br>3J7P_S2:1047-1051_1067-1071<br>4V5O_BA:543-550_581-582<br>4V88_A6:550-557_587-588<br>6FYY_2:549-556_586-587<br>6EK0_S2:599-606_636-637<br>3J7A_A:557-564_594-595<br>5XXB_1:1597-1602_1610-1613<br>5T83_A:69-75_87-89	Known E-loop<br>Known E-loop<br>Known E-loop<br>New E-loop<br>New E-loop<br>New E-loop<br>New E-loop<br>New E-loop<br>New E-loop<br>New E-loop<br>New E-loop<br>New E-loop<br>New E-loop<br>New E-loop<br>Outlier (Rope-sling)<br>Outlier (Rope-sling)<br>Outlier<br>Outlier<br>Outlier (Rope-sling)<br>Outlier<br>Outlier	0.94/7.69	A-G or G-G tH/S,<br>A-U or A-C tH/W,<br>G-A or C-A tS/H
22	4V5O_BA:621-625_946-950<br>4V88_A6:627-631_968-972<br>4V8P_D1:3189-3193_3217-3221<br>3J7A_A:634-638_1037-1041<br>3J7P_S2:676-680_1025-1029<br>5XXU_2:626-630_965-969<br>5XYI_2:549-553_788-792<br>5XXU_2:1538-1542_1565-1569<br>6AZ1_1:676-680_1215-1219<br>5OPT_E:681-685_1303-1307<br>5V7Q_A:2925-2929_2956-2960<br>6FYY_2:626-630_967-971<br>6EK0_S2:676-680_1025-1029<br>6DLR_A:12-15_45-50<br>4V88_A6:1540-1544_1567-1571<br>6FYY_2:1538-1542_1565-1569<br>4Y4O_2A:1417-1421_1577-1581<br>3J7P_S2:41-47_479-481<br>3J7A_A:42-48_437-439<br>6EK0_S2:1281-1287_1314-1316<br>6D9J_2:1281-1287_1314-1316<br>4V88_A6:42-48_431-433<br>6FYY_2:1684-1688_1709-1713	Known E-loop<br>Known E-loop<br>Known E-loop<br>Known E-loop<br>Known E-loop<br>New E-loop<br>New E-loop<br>New E-loop<br>New E-loop<br>New E-loop<br>New E-loop<br>New E-loop<br>New E-loop<br>New E-loop<br>New E-loop<br>New E-loop<br>Outlier (Hook-turn)<br>Outlier<br>Outlier<br>Outlier<br>Outlier<br>Outlier<br>Outlier	1.22/9.09	A-C tH/W or tH/S,<br>U-A tW/H,<br>G-A tS/H

Table S9: RNA motif subclusters belonging to E-loop motif family (continued)

Subcluster ID	Motif location	Motif family	Avg. 3D structure-based RMSD/AL	Common base-pair interactions
36	5J7L_DA:606-610_618-622	Known E-loop	1.34/9.89	A-G tH/S, U-A tW/H or G-A tS/H, G-A tS/H
	4Y4O_2A:1357-1361_1370-1374	Known E-loop		
	4Y4O_2A:606-610_618-622	Known E-loop		
	4IOA_X:616-620_629-633	New E-loop		
	5OPT_E:1325-1329_1345-1349	New E-loop		
	6ERI_AA:616-620_630-634	New E-loop		
	4LFB_A:1303-1307_1330-1334	New E-loop		
	5ZEB_a:1285-1289_1312-1316	New E-loop		
	6HA1_a:1312-1316_1339-1343	New E-loop		
	5NGM_Aa:1313-1317_1340-1344	New E-loop		
	3J7A_A:1837-1841_1864-1868	New E-loop		
	5V93_a:1295-1299_1322-1326	New E-loop		
	5J7L_AA:1303-1307_1330-1334	New E-loop		
	6AZ1_1:1237-1241_1257-1261	New E-loop		
	4V8P_D1:782-786_794-798	New E-loop		
	6HA1_A:650-654_664-668	New E-loop		
	5XYI_2:1347-1351_1373-1377	New E-loop		
	6ERI_BA:1252-1256_1279-1283	New E-loop		
	6D9J_2:1604-1608_1631-1635	New E-loop		
	4V5O_BA:1512-1516_1539-1543	New E-loop		
	6EK0_S2:1604-1608_1631-1635	New E-loop		
	6HA1_A:905-909_962-966	New E-loop		
	4IOA_X:871-875_927-931	Outlier (C-loop)		
	4Y4O_2A:858-862_915-919	Outlier (C-loop)		
44	4Y4O_2A:702-707_724-730	Known E-loop	0.49/13.0	A-G tH/S, A-G tH/S, G-A tS/H
	5V7Q_A:831-836_853-859	New E-loop		
	6HA1_A:749-754_771-777	New E-loop		
	5O60_A:817-822_839-845	New E-loop		
	4V91_1:833-838_855-861	New E-loop		
	5TBW_1:833-838_855-861	New E-loop		
	5T5H_A:1000-1005_1022-1028	New E-loop		
	6AZ3_1:884-889_906-912	New E-loop		
	5O60_A:1322-1329_1349-1353	Outlier		
66	4V88_A6:1678-1682_1719-1723	Known E-loop	1.02/10.0	A-G tH/S or tW/S, G-A tS/H, G-A tS/H
	4V9F_0:794-798_815-819	Known E-loop		
	5TBW_1:1645-1649_1806-1810	New E-loop		
	4V8P_D1:859-863_880-884	New E-loop		
	4V91_1:1645-1649_1806-1810	New E-loop		
	6FYY_2:1676-1680_1717-1721	New E-loop		
	5MRC_A:2732-2736_2746-2750	New E-loop		
	4WF9_X:2765-2769_2789-2793	Outlier		
	6HA1_A:2767-2771_2791-2795	Outlier		
105	5J7L_DA:24-29_511-516	Known E-loop	0.35/12.0	A-G tH/S, G-A tS/H, G-A tS/H, U-A tW/H
	4Y4O_2A:24-29_511-516	Known E-loop		
	4IOA_X:24-29_521-526	Known E-loop		
	6HA1_A:24-29_557-562	New E-loop		
	5O60_A:21-26_598-603	New E-loop		
	4WF9_X:24-29_556-561	New E-loop		
	5V7Q_A:24-29_599-604	New E-loop		
112	3IWN_A:12-16_31-35	Known E-loop	1.47/8.67	A-G tH/S, A-C tS/H
	3MXH_R:22-26_41-45	Known E-loop		
	4IOA_X:303-306_354-359	New E-loop		
	3NDB_M:201-205_216-220	New E-loop		
	3J79_A:2689-2691_3340-3343	Outlier		
132	1MFQ_A:190-194_205-209	Known E-loop	0.47/10.0	G-G tH/S, A-C tH/W, C-A tW/H
	3JAN_4:190-194_205-209	New E-loop		
	6FRK_1:190-194_205-209	New E-loop		
	4V9F_0:1728-1734_2045-2047	Outlier		
	5J7L_AA:1255-1258_1277-1282	Outlier		
141	4WF9_X:1394-1398_1407-1411	Known E-loop	1.49/10.0	A-G tH/S, U-A tW/H, G-G tW/H or tS/H
	5ZEB_a:1240-1244_1253-1257	New E-loop		
	5V93_a:571-575_748-752	New E-loop		
	5ZEB_a:560-564_737-741	New E-loop		

Table S9: RNA motif subclusters belonging to E-loop motif family (continued)

Subcluster ID	Motif location	Motif family	Avg. 3D structure-based RMSD/AL	Common base-pair interactions
175	4WF9_X:1883-1887_1909-1913<br>4IOA_X:1848-1852_1865-1869<br>3J7P_S2:1742-1746_1788-1792<br>3J7A_A:1975-1979_2011-2015<br>6D9J_2:1742-1746_1788-1792<br>3J79_A:1800-1804_2028-2032<br>6HA1_A:1885-1889_1911-1915<br>6EK0_S2:1742-1746_1788-1792<br>5XXB_1:1739-1743_1908-1912	Known E-loop<br>Known E-loop<br>New E-loop<br>New E-loop<br>New E-loop<br>New E-loop<br>New E-loop<br>New E-loop<br>New E-loop	0.82/10.0	A-G tH/S or tW/S,<br>G-A tS/H,<br>G-A tS/H
177	4Y4O_2A:1856-1860_1882-1886<br>2A64_A:284-288_297-301<br>2A64_A:347-351_378-382<br>3DHS_A:284-288_297-301<br>3DHS_A:347-351_378-382<br>5V7Q_A:1955-1958_1970-1974<br>5XXU_2:1676-1680_1710-1714	Known E-loop<br>Known E-loop<br>Known E-loop<br>Known E-loop<br>New E-loop<br>New E-loop<br>New E-loop	0.82/9.67	A-G tH/S,<br>G-A tS/H,<br>G-A tS/H
180	4WF9_X:649-653_663-667<br>5O60_A:1695-1699_1735-1739<br>6ERI_BA:529-533_706-710	Known E-loop<br>New E-loop<br>New E-loop	1.52/9.67	A-G tH/S,<br>U-A tW/H or tW/W
197	5J7L_DA:1357-1361_1370-1374<br>4IOA_X:1370-1374_1383-1387<br>6HA1_A:1396-1400_1409-1413<br>6EK0_L5:2589-2593_2752-2756<br>3J7Q_5:2589-2593_2752-2756<br>6ERI_AA:1874-1877_1888-1892<br>5O60_A:1472-1476_1485-1489<br>5V7Q_A:1488-1492_1501-1505<br>6HA1_a:589-593_766-770<br>3J7P_S2:1098-1102_1130-1134	Known E-loop<br>Known E-loop<br>New E-loop<br>New E-loop<br>New E-loop<br>New E-loop<br>New E-loop<br>New E-loop<br>New E-loop<br>Outlier	1.18/9.56	G-G or A-G tH/S,<br>G-A tS/H or A-U tH/W,<br>G-A or G-U tS/H
202	5J7L_DA:702-707_724-730<br>4WF9_X:747-752_769-775<br>5XXB_1:923-928_945-951<br>6ERI_AA:713-718_735-741<br>3J79_A:952-957_974-980<br>5XY3_1:559-564_581-587	Known E-loop<br>Known E-loop<br>New E-loop<br>New E-loop<br>New E-loop<br>New E-loop	0.45/13.0	A-G tH/S,<br>G-A tS/H,<br>G-A tS/H
266	4UYK_R:86-90_111-115<br>6D9J_5:1551-1555_1572-1576<br>6EK0_L5:1551-1555_1572-1576<br>4GMA_Z:97-101_139-143<br>3J7Q_5:1551-1555_1572-1576	Known E-loop<br>New E-loop<br>New E-loop<br>New E-loop<br>New E-loop	0.94/9.6	A-G tH/S,<br>G-A tS/H,<br>G-A tS/H
318	5J7L_DA:1474-1478_1513-1517<br>4LFB_A:580-584_757-761<br>4V8P_D1:2418-2421_2592-2596<br>5V7Q_A:3082-3087_3100-3104	Known E-loop<br>Known E-loop<br>New E-loop<br>Outlier	1.87/8.67	A-G tH/S,<br>U-A tW/H,<br>G-G tS/H

For each subcluster, the average (avg.) 3D structure-based RMSD, alignment length (AL) and common base-pairs are generated after excluding the outliers.

Table S10: RNA motif subclusters belonging to C-loop motif family

Subcluster ID	Motif location	Motif family	Avg. 3D structure-based RMSD/AL	Common base-pair interactions
37	4V8P_D1:1025-1030_1072-1075<br>5TBW_1:999-1004_1046-1049<br>6D9J_5:1739-1744_1786-1789<br>6EK0_L5:1739-1744_1786-1789<br>3J7Q_5:1739-1744_1786-1789<br>4V91_1:999-1004_1046-1049<br>5XXB_1:1088-1093_1135-1138<br>4V91_1:3004-3007_3139-3144<br>5NGM_Aa:1269-1273_1282-1286<br>5TBW_1:3004-3007_3139-3144	Known C-loop<br>Known C-loop<br>New C-loop<br>New C-loop<br>New C-loop<br>New C-loop<br>New C-loop<br>Outlier<br>Outlier<br>Outlier	0.83/10.0	A-C cW/S,<br>C-A tW/H,<br>A-G tH/S
76	3J7Q_5:1732-1737_1792-1797<br>6AZ3_1:1043-1048_1103-1108<br>6EK0_L5:1732-1737_1792-1797<br>5MRC_A:780-785_841-846<br>5T5H_A:1163-1168_1223-1228	Known C-loop<br>New C-loop<br>New C-loop<br>New C-loop<br>New C-loop	0.71/11.1	A-A tH/S,<br>U-A tW/H,<br>G-U cS/H

Table S10: RNA motif subclusters belonging to C-loop motif family (continued)

Subcluster ID	Motif locatio	Motif family	Avg. 3D structure-based RMSD/AL	Common base-pair interactions
111	4LFB_A:371-375_389-390	Known C-loop	0.80/6.15	A-C or A/G cW/S, C-A tW/H, A-C cH/S, A-C cH/S, A-C or A-G cH/S
	5XXU_2:443-447_460-461	New C-loop		
	6ERI_BA:943-947_994-995	New C-loop		
	5NGM_Aa:379-383_397-398	New C-loop		
	3J7A_A:449-453_466-467	New C-loop		
	5OQL_1:1027-1031_1044-1045	New C-loop		
	5J7L_AA:993-997_1044-1045	New C-loop		
	3J9M_AA:834-838_852-853	New C-loop		
	6AZ1_1:486-490_508-509	New C-loop		
	5OPT_E:490-494_512-513	New C-loop		
	6HA1_a:1003-1007_1054-1055	New C-loop		
	5NGM_Aa:1002-1006_1055-1056	New C-loop		
	6FYY_2:442-446_459-460	New C-loop		
	4LFB_A:993-997_1044-1045	New C-loop		
	6HA1_a:379-383_397-398	New C-loop		
	6ERI_BA:343-347_361-362	New C-loop		
	5MRC_aa:1470-1472_1589-1592	New C-loop		
	4V88_A6:443-447_460-461	New C-loop		
	4V5O_BA:435-439_452-453	New C-loop		
	5XXB_1:2509-2511_3092-3095	Outlier		
	6ERI_BA:605-607_696-699	Outlier		
	6AZ3_2:642-644_1420-1423	Outlier		
	6D9J_5:2843-2846_3842-3845	Outlier		
	4V8P_D1:2391-2393_2969-2972	Outlier		
	5AN9_N:2662-2664_3314-3317	Outlier		
	4V91_1:2396-2398_2981-2984	Outlier		
	5T5H_B:742-744_1551-1554	Outlier		
	6EK0_L5:3900-3902_4558-4561	Outlier		
	5TBW_1:2396-2398_2981-2984	Outlier		
	3J79_A:2689-2691_3340-3343	Outlier		
137	1KOG_O:74-76_96-100	Known C-loop	1.13/7.46	C-G cW/S, C-A tW/H, C-C cS/H
	4JRC_B:29-33_73-75	Known C-loop		
	4MGN_A:29-33_73-75	Known C-loop		
	4V5O_BA:1130-1132_1589-1593	Known C-loop		
	6ERI_AA:2697-2701_2743-2745	New C-loop		
	4LFB_A:933-935_1380-1384	New C-loop		
	6HA1_a:943-945_1389-1393	New C-loop		
	5XYI_2:966-968_1423-1427	New C-loop		
	5J7L_AA:933-935_1380-1384	New C-loop		
	5B2P_B:76-80_88-90	New C-loop		
	6ERI_BA:883-885_1330-1334	New C-loop		
	5XY3_1:2501-2505_2542-2544	New C-loop		
	5V7Q_A:2918-2922_2963-2965	New C-loop		
	5V93_a:926-928_1373-1377	New C-loop		
	5ZEB_a:915-917_1363-1367	New C-loop		
	5NGM_Aa:942-944_1390-1394	New C-loop		
	5O60_A:2904-2908_2949-2951	New C-loop		
	6AZ1_1:58-63_85-87	Outlier		
	5OPT_E:58-63_84-86	Outlier		
	5O60_A:1551-1554_1617-1619	Outlier		
183	4V9F_0:1425-1429_1437-1439	Known C-loop	0.62/4.94	C-A cS/W, U-A cS/H
	3J9M_AA:1486-1488_1562-1565	New C-loop		
	5OPT_E:2173-2175_2278-2281	New C-loop		
	4LFB_A:1402-1404_1497-1500	New C-loop		
	5ZEB_a:1385-1387_1481-1484	New C-loop		
	3J7P_S2:491-495_508-509	New C-loop		
	6HA1_a:1411-1413_1507-1510	New C-loop		
	3J7A_A:1936-1938_2052-2055	New C-loop		
	5XYI_2:374-378_391-392	New C-loop		
	6D9J_2:491-495_508-509	New C-loop		
	5XXU_2:1637-1639_1751-1754	New C-loop		
	5MRC_A:1745-1748_1803-1804	New C-loop		
	5J7L_AA:1402-1404_1497-1500	New C-loop		
	6AZ1_1:2059-2061_2163-2166	New C-loop		
	6GAZ_AA:846-848_923-926	New C-loop		
	5V7Q_A:2306-2308_2676-2680	New C-loop		
	6GAZ_AA:190-194_208-209	New C-loop		
	6EK0_S2:491-495_508-509	New C-loop		
	5XYI_2:1445-1447_1540-1543	New C-loop		
	6ERI_BA:1074-1077_1094-1098	New C-loop		
	6ERI_BA:1352-1354_1447-1450	New C-loop		
	6D9J_2:1703-1705_1829-1832	New C-loop		
	4V88_A6:1639-1641_1760-1763	New C-loop		

Table S10: RNA motif subclusters belonging to C-loop motif family (continued)

Subcluster ID	Motif location	Motif family	Avg. 3D structure-based RMSD/AL	Common base-pair interactions
183	4V5O_BA:1611-1613_1713-1716<br>6FYY_2:1637-1639_1758-1761<br>6EK0_S2:1703-1705_1829-1832<br>3J7P_S2:1703-1705_1829-1832<br>4WF9_X:42-44_480-483<br>6GAW_BA:1290-1293_1303-1306<br>6GAZ_AA:339-341_352-355<br>3J7P_S2:1537-1540_1593-1595<br>5LYS_B:33-35_74-78	New C-loop<br>New C-loop<br>New C-loop<br>New C-loop<br>Outlier<br>Outlier<br>Outlier<br>Outlier<br>Outlier	0.62/4.94	
213	4IOA_X:1838-1841_1876-1877<br>4V8P_C3:28-29_48-51<br>4V9F_9:29-30_50-53<br>5FDV_1B:30-31_51-54<br>5J7L_DA:1846-1849_1893-1894<br>5J7L_DB:30-31_51-54<br>5V7Q_A:2080-2083_2131-2132<br>5V7Q_B:29-30_50-53<br>6EK0_L7:28-29_48-51<br>6AZ3_8:30-31_50-53<br>6DZP_B:31-32_52-55<br>6ERI_AA:1856-1859_1907-1908<br>1U9S_A:133-134_164-167<br>4WF9_Y:28-29_49-52<br>5T5H_D:28-29_48-51<br>3J79_B:28-29_48-51<br>3J7O_7:28-29_48-51<br>6DZI_B:31-32_52-55<br>5XYM_B:31-32_52-55<br>5O60_A:2063-2066_2117-2118<br>6HA1_A:1875-1878_1922-1923<br>6HA1_B:28-29_49-52<br>6D9J_7:28-29_48-51<br>5OQL_1:137-139_199-201<br>3Q1Q_B:126-127_148-151<br>4M4O_B:26-28_39-41<br>4M6D_H:26-28_39-41<br>5XJC_H:112-114_142-144<br>6AHD_H:112-114_142-144	Known C-loop<br>Known C-loop<br>Known C-loop<br>Known C-loop<br>Known C-loop<br>Known C-loop<br>New C-loop<br>New C-loop<br>New C-loop<br>New C-loop<br>New C-loop<br>New C-loop<br>New C-loop<br>New C-loop<br>New C-loop<br>New C-loop<br>New C-loop<br>New C-loop<br>New C-loop<br>New C-loop<br>New C-loop<br>New C-loop<br>New C-loop<br>Outlier<br>Outlier<br>Outlier<br>Outlier<br>Outlier<br>Outlier	0.74/5.47	C-A cS/W,<br>A-A or A-G cS/H
216	4IOA_X:877-881_921-924<br>4WF9_X:909-913_954-957<br>4Y4O_2A:864-868_909-912<br>5J7L_DA:864-868_909-912<br>6HA1_A:911-915_956-959<br>6ERI_AA:874-878_918-921<br>6GAZ_AA:868-870_897-902<br>5GAH_1:38-43_66-68	Known C-loop<br>Known C-loop<br>Known C-loop<br>Known C-loop<br>New C-loop<br>New C-loop<br>Outlier<br>Outlier	0.40/9.0	C-C cW/S,<br>C-A tW/H,<br>C-C cH/S,<br>A-G tW/S or tH/S,
265	3J7A_A:1258-1261_1914-1918<br>3J7P_S2:1214-1217_1681-1685<br>4V88_A6:1157-1160_1617-1621<br>5OPT_E:1625-1628_2151-2155<br>6FYY_2:1156-1159_1615-1619<br>5OQL_1:1741-1744_2200-2204<br>6EK0_S2:1214-1217_1681-1685<br>5XXU_2:1152-1155_1615-1619<br>6AZ1_1:1509-1512_2037-2041<br>6D9J_2:1214-1217_1681-1685<br>5OOL_A:2058-2062_2077-2081<br>5XXB_3:32-36_41-45	Known C-loop<br>Known C-loop<br>Known C-loop<br>New C-loop<br>New C-loop<br>New C-loop<br>New C-loop<br>New C-loop<br>New C-loop<br>New C-loop<br>Outlier<br>Outlier	0.56/9.0	C-A cW/S,<br>C-A tW/H,<br>C-C cS/H,<br>U-C cS/H
292	4V9F_0:958-963_1005-1008	Known C-loop	0/10	C-C cS/W, A-C tH/W,<br>C-C cS/H, G-A tS/W
319	4WF9_X:2707-2711_2752-2754<br>4Y4O_2A:2680-2684_2725-2727<br>4LFB_A:1124-1127_1145-1149<br>6HA1_a:1134-1137_1154-1158<br>6ERI_AA:292-296_363-367	Known C-loop<br>Known C-loop<br>New C-loop<br>New C-loop<br>Outlier	0.76/6.0	C-G cW/S,<br>C-A tW/H,<br>C-C cS/H
334	5J7L_DA:857-862_915-920<br>5V7Q_A:986-991_1044-1049<br>4WF9_X:902-907_960-965<br>5O60_A:972-977_1030-1035	Known C-loop<br>New C-loop<br>New C-loop<br>New C-loop	0.66/12.0	A-G tH/S,<br>U-A tW/H,<br>G-U cS/H
360	4V8P_D1:1018-1023_1078-1083	Known C-loop	0/12.0	A-A tH/S, U-A tW/H,<br>G-U cS/H

Table S10: RNA motif subclusters belonging to C-loop motif family (continued)

Subcluster ID	Motif location	Motif family	Avg. 3D structure-based RMSD/AL	Common base-pair interactions
397	5MRC_A:787-791_835-838<br>4V8P_D1:3036-3040_3080-3083<br>4V91_1:3047-3051_3091-3094<br>5TBW_1:3047-3051_3091-3094<br>4IOA_X:1451-1454_1567-1571	Known C-loop<br>New C-loop<br>New C-loop<br>New C-loop<br>Outlier	0.78/9.0	C-A tW/H or A-A tH/H
400	4IOA_X:2659-2663_2705-2707<br>4V9F_0:2717-2721_2761-2763<br>5OOL_A:3148-3152_3161-3163<br>6AZ1_1:115-119_340-343<br>6HA1_A:2709-2713_2754-2756<br>4IOA_Y:32-33_53-56<br>3J9M_AA:986-988_999-1002<br>3J7Q_5:4996-4997_5052-5055	Known C-loop<br>Known C-loop<br>New C-loop<br>New C-loop<br>New C-loop<br>Outlier<br>Outlier<br>Outlier	0.67/6.70	C-G cW/S,<br>C-C cS/H,<br>C-A tW/H

For each subcluster, the average (avg.) 3D structure-based RMSD, alignment length (AL) and common base-pairs are generated after excluding the outliers.

Table S11: RNA motif subclusters belonging to GNRA motif family

Subcluster ID	Motif location	Motif family	Avg. 3D structure-based RMSD/AL	Common base-pair interactions
0	4NLF_A:2658-2663<br>4IOA_X:2637-2642<br>4LVW_A:58-63<br>4V9F_0:1326-1331<br>3NPQ_A:20-25<br>4WF9_X:2685-2690<br>5J7L_DA:2658-2663<br>4V8P_D1:3014-3019<br>4V9F_0:2695-2700<br>4V88_A6:1501-1506<br>3AM1_B:45-50<br>4Y4O_2A:2658-2663<br>4OQU_A:76-81<br>5O60_A:2882-2887<br>5TBW_1:3025-3030<br>6AZ3_4:53-58<br>5XXB_1:3137-3142<br>6DME_A:25-30<br>6FYY_2:1499-1504<br>3J79_A:3411-3416<br>5OQL_1:2084-2089<br>5AN9_N:3361-3366<br>6ERI_AA:2675-2680<br>6HA1_A:2687-2692<br>1F1T_A:14-19<br>6ERI_BA:268-273<br>3J7Q_5:4603-4608<br>5V7Q_A:2896-2901<br>4V91_1:3025-3030	Known GNRA loop<br>Known GNRA loop<br>Known GNRA loop<br>Known GNRA loop<br>Known GNRA loop<br>Known GNRA loop<br>Known GNRA loop<br>Known GNRA loop<br>Known GNRA loop<br>Known GNRA loop<br>Known GNRA loop<br>Known GNRA loop<br>Known GNRA loop<br>New GNRA loop<br>New GNRA loop<br>New GNRA loop<br>New GNRA loop<br>New GNRA loop<br>New GNRA loop<br>New GNRA loop<br>New GNRA loop<br>New GNRA loop<br>New GNRA loop<br>New GNRA loop<br>New GNRA loop<br>New GNRA loop<br>New GNRA loop<br>New GNRA loop<br>New GNRA loop	0.5/6	G-A tS/H
1	3KTW_C:209-214<br>1G1X_J:726-731<br>5J7L_AA:726-731<br>2XXA_F:47-52<br>4LFB_A:726-731<br>3NDB_M:208-213<br>1G1X_E:726-731<br>4WF9_X:533-538<br>4OQU_A:57-62<br>4OQU_A:13-18<br>3J79_B:86-91<br>4IOA_X:1235-1240<br>4C7O_E:19-24<br>5MRC_aa:1607-1612<br>5OPT_E:1272-1277<br>5XYI_2:757-762<br>5NGM_Aa:1526-1531<br>6AZ1_1:1184-1189<br>4LFB_A:1515-1520<br>3J7O_7:86-91<br>6AGB_A:177-182	Known GNRA loop<br>Known GNRA loop<br>Known GNRA loop<br>Known GNRA loop<br>Known GNRA loop<br>Known GNRA loop<br>Known GNRA loop<br>Known GNRA loop<br>Known GNRA loop<br>Known GNRA loop<br>Known GNRA loop<br>Known GNRA loop<br>Known GNRA loop<br>New GNRA loop<br>New GNRA loop<br>New GNRA loop<br>New GNRA loop<br>New GNRA loop<br>New GNRA loop<br>New GNRA loop<br>New GNRA loop	0.68/5.34	G-A tS/H or G-G tS/H<br>or G-A tW/H or G-G tS/H

Table S11: RNA motif subclusters belonging to GNRA motif family (continued)

Subcluster ID	Motif location	Motif family	Avg. 3D structure-based RMSD/AL	Common base-pair interactions
1	6HA1_A:534-539	New GNRA loop		
	5J7L_AA:1515-1520	New GNRA loop		
	5V93_a:717-722	New GNRA loop		
	6HA1_a:1525-1530	New GNRA loop		
	5GAH_1:52-57	New GNRA loop		
	6HA1_a:735-740	New GNRA loop		
	3R9W_B:1515-1520	New GNRA loop		
	6D9J_7:86-91	New GNRA loop		
	5ZEB_a:1499-1504	New GNRA loop		
	6HA1_A:1262-1267	New GNRA loop		
	5NGM_Aa:734-739	New GNRA loop		
	1DUH_A:52-57	Outlier		
4	1XJR_A:21-27	Known GNRA loop	0.71/5.89	G-A tS/H
	3J79_A:1254-1260	Known GNRA loop		
	5TBW_1:1126-1132	Known GNRA loop		
	4V8P_D1:1153-1159	Known GNRA loop		
	4V9F_0:1054-1060	Known GNRA loop		
	5OOL_A:1746-1752	Known GNRA loop		
	4V8P_D1:88-94	Known GNRA loop		
	5XXB_1:1213-1219	New GNRA loop		
	5AN9_N:1363-1369	New GNRA loop		
	3J7Q_5:1864-1870	New GNRA loop		
	5T5H_A:1309-1315	New GNRA loop		
	5XY3_1:850-856	New GNRA loop		
	5XY3_1:88-94	New GNRA loop		
	6AZ3_1:1183-1189	New GNRA loop		
	4V91_1:1126-1132	New GNRA loop		
8	4LCK_B:71-76	Known GNRA loop		
	6CHR_A:173-178	Known GNRA loop		
	5D5L_A:8-13	Known GNRA loop		
	3LQX_B:153-158	Known GNRA loop		
	2IL9_A:6119-6119.E	Known GNRA loop		
	1HMH_E:104-114	Known GNRA loop		
	2OIU_Q:10-15	Known GNRA loop		
	2OIU_P:10-15	Known GNRA loop		
	1RLG_D:9-14	Known GNRA loop		
	3NVI_F:9-14	Known GNRA loop		
	1HMH_A:104-114	Known GNRA loop		
	4FRG_B:46-51	Known GNRA loop		
	5J7L_DA:2374-2379	Known GNRA loop		
	4GMA_Z:119-124	Known GNRA loop		
	3E5C_A:41-46	Known GNRA loop		
	4QK8_A:13-18	Known GNRA loop		
	3NKB_B:49-54	Known GNRA loop		
	3P22_A:17-22	Known GNRA loop		
	3IWN_A:21-26	Known GNRA loop		
	4RUM_A:51-56	Known GNRA loop		
	3CUL_C:7-12	Known GNRA loop		
	5TPY_A:13-18	Known GNRA loop		
	3NMU_E:18-23	Known GNRA loop		
	4Y1J_A:76-81	Known GNRA loop		
	3D0U_A:142-147	Known GNRA loop		
	4KQY_A:60-65	Known GNRA loop		
	4V8P_D1:2733-2738	Known GNRA loop		
	1G1X_D:590-649	Known GNRA loop		
	4LFB_A:1165-1171	Known GNRA loop		
	5TPY_A:62-67	Known GNRA loop		
	4Y4O_2A:1363-1368	Known GNRA loop		
	3SUX_X:65-70	Known GNRA loop		
	5M0J_E:12-17	Known GNRA loop		
	4WF9_X:2401-2406	Known GNRA loop		
	5M0I_E:12-17	Known GNRA loop		
	2OIU_P:28-33	Known GNRA loop		
	4V9F_0:2411-2416	Known GNRA loop		
	4V88_A6:937-942	Known GNRA loop		
	2OIU_P:59-64	Known GNRA loop		
	3MXH_R:31-36	Known GNRA loop		
	5M0I_F:12-17	Known GNRA loop		
	1KH6_A:28-33	Known GNRA loop		
	2OIU_Q:59-64	Known GNRA loop		
	4V9F_0:804-809	Known GNRA loop		
	4IOA_X:640-645	Known GNRA loop		
	4W90_C:82-87	Known GNRA loop		

Table S11: RNA motif subclusters belonging to GNRA motif family (continued)

Subcluster ID	Motif location	Motif family	Avg. 3D structure-based RMSD/AL	Common base-pair interactions
8	4PQV_A:13-18	Known GNRA loop	0.50/5.95	G-A tS/H or tW/H
	1G1X_I:590-649	Known GNRA loop		
	4WF9_X:674-679	Known GNRA loop		
	4Y1M_B:24-29	Known GNRA loop		
	5U3G_B:47-52	Known GNRA loop		
	5J7L_DA:1363-1368	Known GNRA loop		
	4PLX_A:24-29	Known GNRA loop		
	4V8P_D1:732-737	Known GNRA loop		
	4V5O_BA:915-920	Known GNRA loop		
	5M0J_F:12-17	Known GNRA loop		
	4JF2_A:8-13	Known GNRA loop		
	4BW0_A:9-14	Known GNRA loop		
	4P8Z_A:157-162	Known GNRA loop		
	4QLM_A:54-59	Known GNRA loop		
	4Y4O_2A:2374-2379	Known GNRA loop		
	6ERI_AA:641-646	New GNRA loop		
	6GAZ_AA:376-381	New GNRA loop		
	6CB3_B:76-81	New GNRA loop		
	3J7A_A:1797-1802	New GNRA loop		
	6GAZ_AA:198-203	New GNRA loop		
	6HA1_A:2403-2408	New GNRA loop		
	5Y7M_D:24-29	New GNRA loop		
	3J9M_AA:1023-1028	New GNRA loop		
	6ERI_BA:351-356	New GNRA loop		
	6FYY_2:936-941	New GNRA loop		
	6GAW_BA:1461-1466	New GNRA loop		
	5O60_A:723-728	New GNRA loop		
	6HA1_A:675-680	New GNRA loop		
	6AZ3_2:1173-1178	New GNRA loop		
	5UZ6_A:15-20	New GNRA loop		
	5XXU_2:934-939	New GNRA loop		
	3J7A_A:1006-1011	New GNRA loop		
	6ERI_BA:675-680	New GNRA loop		
	5U3G_B:18-23	New GNRA loop		
	6ERI_AA:2391-2396	New GNRA loop		
	6AZ1_1:1434-1439	New GNRA loop		
	2OIU_Q:28-33	New GNRA loop		
	6CU1_A:42-47	New GNRA loop		
	6HA1_a:387-392	New GNRA loop		
	5XY3_1:2198-2203	New GNRA loop		
	5OOL_A:3126-3131	New GNRA loop		
	5OPT_E:1550-1555	New GNRA loop		
	3J79_A:963-968	New GNRA loop		
	5MRC_aa:383-388	New GNRA loop		
	5O60_A:2598-2603	New GNRA loop		
	5T5H_B:1304-1309	New GNRA loop		
	5VCI_A:16-21	New GNRA loop		
	5V7Q_A:733-738	New GNRA loop		
	5B2P_B:35-40	New GNRA loop		
	5J7L_DA:1806-1811	New GNRA loop		
	6HA1_A:1402-1407	New GNRA loop		
	2IL9_A:6084.A-6084.F	New GNRA loop		
	3J9M_AA:842-847	New GNRA loop		
	283D_A:4-9	Outlier		
9	4Y4O_2A:629-634	Known GNRA loop		
	1U6B_B:23-28	Known GNRA loop		
	1UN6_F:82-94	Known GNRA loop		
	2YGH_A:73-78	Known GNRA loop		
	3IGI_A:274-279	Known GNRA loop		
	4V9F_0:690-695	Known GNRA loop		
	1MFQ_A:197-202	Known GNRA loop		
	5TBW_1:844-849	Known GNRA loop		
	3RW6_H:27-32	Known GNRA loop		
	5FJC_A:73-78	Known GNRA loop		
	1Y0Q_A:204-209	Known GNRA loop		
	4V9F_0:1628-1633	Known GNRA loop		
	4LFB_A:158-163	Known GNRA loop		
	5DCV_D:26-31	Known GNRA loop		
	6CHR_A:369-374	Known GNRA loop		
	5J7L_DA:629-634	Known GNRA loop		
	4V8P_D1:869-874	Known GNRA loop		
	3MOJ_A:2529-2534	Known GNRA loop		
	5DDO_B:11-16	Known GNRA loop		

Table S11: RNA motif subclusters belonging to GNRA motif family (continued)

Subcluster ID	Motif location	Motif family	Avg. 3D structure-based RMSD/AL	Common base-pair interactions
9	5DDP_A:11-16	Known GNRA loop	0.51/5.89	G-A tS/H or tS/W or tW/W
	5DDO_A:11-16	Known GNRA loop		
	3IAB_R:78-83	Known GNRA loop		
	4Y1J_A:20-25	Known GNRA loop		
	1M5K_B:74-79	Known GNRA loop		
	3V7E_C:105-110	Known GNRA loop		
	5J7L_AA:158-163	Known GNRA loop		
	5CZZ_B:34-39	Known GNRA loop		
	3E5C_A:18-23	Known GNRA loop		
	3Q1Q_B:135-140	Known GNRA loop		
	4AOB_A:73-78	Known GNRA loop		
	4IOA_X:2353-2358	Known GNRA loop		
	2R8S_R:149-154	Known GNRA loop		
	6D9J_5:1561-1566	New GNRA loop		
	6ERI_BA:143-148	New GNRA loop		
	3JAN_4:197-202	New GNRA loop		
	6AZ3_1:895-900	New GNRA loop		
	3OXE_A:49-54	New GNRA loop		
	5XY3_1:570-575	New GNRA loop		
	3J7P_S2:1141-1146	New GNRA loop		
	5XTM_D:24-29	New GNRA loop		
	6HA1_a:157-162	New GNRA loop		
	6CB3_B:20-25	New GNRA loop		
	4V91_1:844-849	New GNRA loop		
	6FRK_1:197-202	New GNRA loop		
	5XTM_B:24-29	New GNRA loop		
	5T5H_A:1011-1016	New GNRA loop		
	4KR9_X:19-24	New GNRA loop		
	5V93_a:154-159	New GNRA loop		
	5AN9_N:3077-3082	New GNRA loop		
	6CB3_B:50-55	New GNRA loop		
	3J7Q_5:1561-1566	New GNRA loop		
	5ZEB_a:155-160	New GNRA loop		
	5T2A_B:1108-1113	New GNRA loop		
	4Y1J_A:50-55	Outlier		
	5G2X_A:322-327	Outlier		
10	5MRC_A:2861-2866	Known GNRA loop	0.61/5.49	G-A tS/H or tS/W or tW/W
	5F9F_F:10-15	Known GNRA loop		
	4V5O_BA:1268-1273	Known GNRA loop		
	4V88_A6:1296-1301	Known GNRA loop		
	5J7L_AA:1076-1081	Known GNRA loop		
	5TBW_1:357-362	Known GNRA loop		
	4V9F_0:468-473	Known GNRA loop		
	5OPT_E:1764-1769	New GNRA loop		
	5O60_A:1336-1341	New GNRA loop		
	3JCS_3:5-10	New GNRA loop		
	6AZ3_3:5-10	New GNRA loop		
	6AZ1_1:1648-1653	New GNRA loop		
	6AZ3_8:88-93	New GNRA loop		
	6ERI_AB:60-65	New GNRA loop		
	3T4B_A:294-299	New GNRA loop		
	5FLX_z:294-299	New GNRA loop		
	5XXU_2:1291-1296	New GNRA loop		
	4V91_1:357-362	New GNRA loop		
	6FYY_2:1295-1300	New GNRA loop		
	5XY3_1:330-335	New GNRA loop		
	1OOA_C:13-18	New GNRA loop		
11	4V88_A6:1121-1126	Known GNRA loop	0.53/5.83	G-A tS/H or tS/W or tW/W
	4LFB_A:379-384	Known GNRA loop		
	3W3S_B:47.F-47.K	Known GNRA loop		
	4LFB_A:897-902	Known GNRA loop		
	3J7A_A:1222-1227	Known GNRA loop		
	5J7L_AA:379-384	Known GNRA loop		
	5J7L_AA:186-191	Known GNRA loop		
	4V8P_D1:757-762	Known GNRA loop		
	5J7L_AA:897-902	Known GNRA loop		
	2EZ6_D:11-16	Known GNRA loop		
	4V5O_BA:1094-1099	Known GNRA loop		
	1MZP_B:25-30	Known GNRA loop		
	4V9F_0:1862-1867	Known GNRA loop		
	5J7L_DA:1868-1873	Known GNRA loop		
	6ERI_BA:847-852	New GNRA loop		

Table S11: RNA motif subclusters belonging to GNRA motif family (continued)

Subcluster ID	Motif location	Motif family	Avg. 3D structure-based RMSD/AL	Common base-pair interactions
11	4V91_1:732-737<br>6FYY_2:1120-1125<br>5OOL_A:1786-1791<br>6HA1_a:907-912<br>5O60_A:2023-2028<br>5ZEB_a:879-884<br>5XYI_2:930-935<br>5MRC_A:2925-2930<br>5XXU_2:1116-1121<br>6HA1_A:2823-2828<br>5V93_a:378-383<br>5V7Q_A:2040-2045<br>6ERI_BA:1399-1404<br>5TBW_1:732-737<br>5ZEB_a:379-384<br>5OPT_E:1589-1594<br>4V91_1:2470-2475<br>5V93_a:890-895<br>6AZ1_1:1473-1478<br>5NGM_Aa:906-911<br>2NUE_C:21-26	New GNRA loop<br>New GNRA loop	0.53/5.83	G-A tS/H or tS/W or tW/W
12	3Q1Q_B:187-192<br>4WF9_X:1400-1405<br>1U9S_A:204-209<br>4LFB_A:1265-1270<br>6FYY_2:413-418<br>6D9J_2:462-467<br>4V88_A6:414-419<br>3IGI_A:368-373<br>5OPT_E:212-217<br>5O60_A:1478-1483<br>3J7P_S2:462-467<br>5XY3_1:1306-1311<br>5V7Q_A:1494-1499	Known GNRA loop<br>Known GNRA loop<br>Known GNRA loop<br>New GNRA loop<br>New GNRA loop<br>New GNRA loop<br>New GNRA loop<br>New GNRA loop<br>New GNRA loop<br>New GNRA loop<br>New GNRA loop<br>New GNRA loop<br>New GNRA loop	0.74/5.37	G-A tS/H or G-A tW/H<br>or G-A tS/W or C-G tS/W
24	4V9F_0:2876-2881<br>1Y0Q_A:130-135<br>4WFL_A:81-86<br>3J79_A:1783-1788<br>4IOA_X:1856-1861<br>3Q1Q_B:284-289<br>4Y4O_2A:1864-1878<br>1U9S_A:99-104<br>4V8P_D1:1658-1663<br>4WZJ_XXX:50-55<br>4V8P_D1:356-361<br>5J7L_DA:2856-2861<br>5XXU_2:414-419<br>6HA1_A:1764-1769<br>5XXU_2:1499-1504<br>3J7P_S2:1353-1358<br>5TBW_1:1634-1639<br>5XXB_1:1728-1733<br>5T2A_B:403-408<br>3J7Q_5:2578-2583<br>6AZ3_2:466-471<br>3J7A_A:420-425<br>1Y0Q_A:146-151<br>4V91_1:1634-1639<br>3DD2_B:11-16	Known GNRA loop<br>Known GNRA loop<br>Known GNRA loop<br>Known GNRA loop<br>Known GNRA loop<br>Known GNRA loop<br>Known GNRA loop<br>Known GNRA loop<br>Known GNRA loop<br>Known GNRA loop<br>Known GNRA loop<br>Known GNRA loop<br>New GNRA loop<br>New GNRA loop<br>New GNRA loop<br>New GNRA loop<br>New GNRA loop<br>New GNRA loop<br>New GNRA loop<br>New GNRA loop<br>New GNRA loop<br>New GNRA loop<br>New GNRA loop<br>New GNRA loop<br>Outlier	0.61/5.51	G-A tS/H or G-A tW/H<br>or U-G tS/W
25	5MRC_A:393-398<br>4R4V_A:735-740<br>5MRC_A:2641-2646<br>4WF9_X:1260-1265<br>4Y4O_2A:1806-1811<br>4QK9_A:56-61<br>6GAW_BA:121-126<br>4V91_1:1403-1408<br>4C8Z_C:20-25<br>6HA1_A:1835-1840<br>5TBW_1:1403-1408<br>3J7Q_5:368-373<br>5TBW_1:599-604<br>6ERI_AA:1816-1821	Known GNRA loop<br>Known GNRA loop<br>Known GNRA loop<br>Known GNRA loop<br>Known GNRA loop<br>Known GNRA loop<br>New GNRA loop<br>New GNRA loop<br>New GNRA loop<br>New GNRA loop<br>New GNRA loop<br>New GNRA loop<br>New GNRA loop<br>New GNRA loop	0.48/5.59	G-A tS/H or G-A tW/H<br>or U-G tS/W

Table S11: RNA motif subclusters belonging to GNRA motif family (continued)

Subcluster ID	Motif location	Motif family	Avg. 3D structure-based RMSD/AL	Common base-pair interactions
25	5XXB_1:1500-1505	New GNRA loop		
	6ERI_BA:1026-1031	New GNRA loop		
	6HA1_A:1897-1902	New GNRA loop		
	4WF9_X:1833-1838	New GNRA loop		
	4C8Y_C:20-25	New GNRA loop		
	359D_B:154-164	New GNRA loop		
	4V91_1:599-604	New GNRA loop		
27	4WF9_X:2621-2626	Known GNRA loop	0.35/5.97	G-A tS/H or tW/H or tS/W
	3J79_A:365-370	Known GNRA loop		
	4V9F_0:2629-2634	Known GNRA loop		
	5J7L_DA:462-467	Known GNRA loop		
	4LFB_A:1076-1081	Known GNRA loop		
	3J79_A:3322-3327	Known GNRA loop		
	4V8P_D1:1429-1434	Known GNRA loop		
	3J7Q_5:4540-4545	Known GNRA loop		
	4Y4O_2A:2594-2599	Known GNRA loop		
	4V8P_D1:2951-2956	Known GNRA loop		
	4Y4O_2A:462-467	Known GNRA loop		
	5J7L_DA:2594-2599	Known GNRA loop		
	4IOA_X:473-478	Known GNRA loop		
	5TBW_1:2963-2968	Known GNRA loop		
	3J79_A:1551-1556	Known GNRA loop		
	4IOA_X:2573-2578	Known GNRA loop		
	4WF9_X:508-513	Known GNRA loop		
	6AZ3_1:397-402	New GNRA loop		
	5O60_A:2818-2823	New GNRA loop		
	5V7Q_A:551-556	New GNRA loop		
	6HA1_A:2623-2628	New GNRA loop		
	5T5H_A:1627-1632	New GNRA loop		
	5XXB_1:3074-3079	New GNRA loop		
	6ERI_AA:474-479	New GNRA loop		
	5XXB_1:365-370	New GNRA loop		
	5AN9_N:3296-3301	New GNRA loop		
	6ERI_AA:1243-1248	New GNRA loop		
	5O60_A:550-555	New GNRA loop		
	5NGM_Aa:1087-1092	New GNRA loop		
	5T5H_B:1533-1538	New GNRA loop		
	6AZ3_1:1494-1499	New GNRA loop		
	5ZEB_a:1246-1251	New GNRA loop		
	5ZEB_a:1056-1061	New GNRA loop		
	5XY3_1:2418-2423	New GNRA loop		
	6ERI_AA:2611-2616	New GNRA loop		
	4V91_1:2963-2968	New GNRA loop		
	6HA1_a:1086-1091	New GNRA loop		
	5V93_a:1067-1072	New GNRA loop		
	5V7Q_A:2832-2837	New GNRA loop		
	5T2A_B:1337-1342	New GNRA loop		
	4LGT_E:2594-2599	New GNRA loop		
	6AZ3_2:1402-1407	New GNRA loop		
	5T5H_A:401-406	New GNRA loop		
	6HA1_A:509-514	New GNRA loop		
28	2ZZM_B:47-47.E	Known GNRA loop	0.44/6	G-A tS/H or G-G tS/H or G-A tW/H
	3Q1Q_B:92-97	Known GNRA loop		
	5J7L_DA:2356-2361	Known GNRA loop		
	4WF9_X:2876-2881	Known GNRA loop		
	5TBW_AS:88-93	Known GNRA loop		
	3G9C_Q:108-113	Known GNRA loop		
	4V91_3:88-93	New GNRA loop		
	3J7P_S2:1421-1426	New GNRA loop		
35	4V9F_0:576-581	Known GNRA loop	0.41/6	G-A tS/H
	4YAZ_R:25-30	Known GNRA loop		
	4V9F_9:89-94	Known GNRA loop		
	4Y4O_2A:2856-2861	Known GNRA loop		
	5J7L_AA:1265-1270	Known GNRA loop		
	4Y4O_2A:1222-1227	Known GNRA loop		
	6ERI_BA:1214-1219	New GNRA loop		
	6HA1_a:1274-1279	New GNRA loop		
	5V93_a:1257-1262	New GNRA loop		
	3J7Q_5:2561-2566	New GNRA loop		
	5NGM_Aa:1275-1280	New GNRA loop		

Table S11: RNA motif subclusters belonging to GNRA motif family (continued)

Subcluster ID	Motif location	Motif family	Avg. 3D structure-based RMSD/AL	Common base-pair interactions
36	3IGI_A:89-94	Known GNRA loop	0.74/5.25	G-A tS/H or G-G tS/H or C-G tS/W
	5FDV_1B:86-91	Known GNRA loop		
	2Z75_B:113-118	Known GNRA loop		
	4IOA_Y:88-93	Known GNRA loop		
	6HA1_A:2881-2886	New GNRA loop		
	3J7Q_5:4102-4107	New GNRA loop		
	6D9J_2:1565-1570	New GNRA loop		
	3J7P_S2:1565-1570	New GNRA loop		
	361D_B:86-90	New GNRA loop		
	361D_A:86-90	New GNRA loop		
	5XY3_3:86-91	New GNRA loop		
43	4PCJ_A:16-21	Known GNRA loop	0.43/6.0	G-A tS/H
	6CHR_A:563-568	Known GNRA loop		
	5TBW_1:2744-2749	Known GNRA loop		
	4K50_B:587-592	Known GNRA loop		
	4K27_U:26-31	Known GNRA loop		
	4LFB_A:1012-1017	Known GNRA loop		
	5TBW_1:707-712	Known GNRA loop		
	5M0H_A:21-26	Known GNRA loop		
	6CHR_A:481-486	Known GNRA loop		
	5BTM_A:26-31	Known GNRA loop		
	1KXK_A:33-38	Known GNRA loop		
	3J79_A:3083-3088	Known GNRA loop		
	6AZ3_1:745-750	New GNRA loop		
	5XXB_1:2855-2860	New GNRA loop		
	4V91_1:2744-2749	New GNRA loop		
	5MRC_aa:302-307	New GNRA loop		
	5T5H_A:842-847	New GNRA loop		
	6ERI_BA:962-967	New GNRA loop		
	6D9J_5:4321-4326	New GNRA loop		
	6AZ4_A:20-25	New GNRA loop		
	3J7Q_5:4321-4326	New GNRA loop		
	5XXB_1:794-799	New GNRA loop		
	4V91_1:707-712	New GNRA loop		
44	1Q96_A:13-18	Known GNRA loop	0.65/5.99	G-A tS/H or G-A tW/H or G-G tS/H
	6CHR_A:600-605	Known GNRA loop		
	5T5H_A:427-432	New GNRA loop		
	5XXB_1:390-395	New GNRA loop		
	3J79_A:391-396	New GNRA loop		
	5NGM_Aa:1021-1026	New GNRA loop		
	6AZ3_1:423-428	New GNRA loop		
	6ERI_BA:170-175	New GNRA loop		
	5AN9_N:2486-2491	New GNRA loop		
	5J7L_AA:1012-1017	New GNRA loop		
	3J7Q_5:393-398	New GNRA loop		
	6HA1_a:1022-1027	New GNRA loop		
	3J9M_AA:1580-1585	New GNRA loop		
	5ZEB_a:995-1000	New GNRA loop		
	6D9J_5:393-398	New GNRA loop		
	5T5H_E:4-9	New GNRA loop		
	6GAZ_AA:941-946	New GNRA loop		
53	4WF9_X:686-692	Known GNRA loop	1.14/5.8	G-A tS/H or A-A tS/H or U-A tS/H or G-A tW/H
	6HA1_A:687-693	New GNRA loop		
	5XY3_1:1527-1533	New GNRA loop		
	3J9M_AA:1281-1287	New GNRA loop		
	4WF9_X:1674-1680	New GNRA loop		
	6HA1_A:826-832	New GNRA loop		
	4JYA_Y:32-38	New GNRA loop		
	5O60_A:735-741	New GNRA loop		
	6ERI_AA:790-796	New GNRA loop		
	5V7Q_A:745-751	Outlier		
55	6CHR_A:390-395	Known GNRA loop	0.92/5.59	G-A tS/H or tW/H
	4IOA_X:2831-2836	Known GNRA loop		
	4V8P_D1:2531-2536	Known GNRA loop		
	4V8P_D1:582-587	Known GNRA loop		
	6GAW_BA:1417-1422	New GNRA loop		
	5MRC_aa:1123-1128	New GNRA loop		
	5XYI_2:566-571	New GNRA loop		
	6D9J_2:1178-1183	New GNRA loop		
	5MRC_aa:962-967	New GNRA loop		
	3J7P_S2:1178-1183	New GNRA loop		

Table S11: RNA motif subclusters belonging to GNRA motif family (continued)

Subcluster ID	Motif location	Motif family	Avg. 3D structure-based RMSD/AL	Common base-pair interactions
55	5OQL_2:141-146<br>6HA1_A:228-233	New GNRA loop<br>Outlier		
56	4P8Z_A:99-104<br>4LFB_A:862-867<br>5J7L_AA:862-867<br>5V93_a:855-860<br>5NGM_Aa:871-876<br>5V7Q_A:767-772<br>6ERI_BA:812-817<br>4V8P_D1:2215-2220<br>5ZEB_a:844-849<br>6HA1_a:872-877<br>4IOA_X:2335-2340	Known GNRA loop<br>Known GNRA loop<br>Known GNRA loop<br>New GNRA loop<br>New GNRA loop<br>New GNRA loop<br>New GNRA loop<br>New GNRA loop<br>New GNRA loop<br>New GNRA loop<br>New GNRA loop	0.52/6	U-C tS/H or U-C tW/H or<br>G-A tS/H or A-C tS/H
61	3D0U_A:121-127<br>4V9F_0:493-499<br>5J7L_DA:611-617<br>3DIL_A:124-130<br>5TBW_1:1023-1029<br>5V7Q_A:1865-1871<br>5MRC_A:1713-1719<br>3J7Q_5:2810-2816<br>4V91_1:1023-1029<br>4V91_1:1862-1868<br>6HA1_a:472-478<br>5TBW_1:1862-1868<br>6HA1_x:49-55<br>5XXB_4:108-114<br>1N33_Y:32-38<br>6D9J_5:2810-2816<br>6D9J_3:49-55	Known GNRA loop<br>Known GNRA loop<br>New GNRA loop<br>New GNRA loop<br>New GNRA loop<br>New GNRA loop<br>New GNRA loop<br>New GNRA loop<br>New GNRA loop<br>New GNRA loop<br>New GNRA loop<br>New GNRA loop<br>New GNRA loop<br>New GNRA loop<br>New GNRA loop<br>New GNRA loop<br>Outlier	1.09/5.93	G-A tS/H or G-A tW/H or G-A tS/W<br>C-A tS/H or A-A tS/H<br>or U-A tW/H or A-U tS/H
76	5TBW_1:90-96<br>4Y4O_2A:246-252<br>4IOA_X:223-229<br>3J79_A:90-96<br>5J7L_DA:487-493<br>5J7L_DA:246-252<br>4WF9_X:249-255<br>5T5H_A:90-96<br>3J7Q_5:89-95<br>6ERI_AA:231-237<br>6HA1_A:249-255<br>5XXB_1:90-96<br>6D9J_5:89-95<br>6AZ3_1:89-95<br>4V91_1:90-96	Known GNRA loop<br>Known GNRA loop<br>Known GNRA loop<br>Known GNRA loop<br>Known GNRA loop<br>Known GNRA loop<br>Known GNRA loop<br>New GNRA loop<br>New GNRA loop<br>New GNRA loop<br>New GNRA loop<br>New GNRA loop<br>New GNRA loop<br>New GNRA loop<br>New GNRA loop	0.46/6.96	G-A tS/H
80	4IOA_X:498-503<br>4Y4O_2A:487-493<br>4V8P_C3:86-91<br>4V9F_0:2248-2253<br>5XXB_3:86-91<br>5V7Q_A:576-581<br>5O60_A:575-580	Known GNRA loop<br>Known GNRA loop<br>Known GNRA loop<br>Known GNRA loop<br>New GNRA loop<br>New GNRA loop<br>New GNRA loop	0.35/6	G-A tS/H
83	4V88_A6:1396-1402<br>3CUL_C:70-76<br>3J7A_A:1642-1648<br>6FYY_2:1394-1400	Known GNRA loop<br>New GNRA loop<br>New GNRA loop<br>New GNRA loop	1.03/5.7	U-A tS/H or U-A tW/H<br>or G-A tS/H
86	5TBW_1:2164-2170<br>5J7L_DA:1630-1636<br>5O60_A:1848-1854<br>4Y4O_2A:779-785<br>6HA1_A:1001-1008<br>6GAW_BA:326-332<br>4IOA_X:792-798<br>6HA1_A:656-662<br>5MRC_A:670-676<br>5LM7_G:21-27<br>6HA1_A:1675-1681<br>6ERI_AA:1666-1672<br>4V91_1:2164-2170	Known GNRA loop<br>Known GNRA loop<br>New GNRA loop<br>New GNRA loop<br>New GNRA loop<br>New GNRA loop<br>New GNRA loop<br>New GNRA loop<br>New GNRA loop<br>New GNRA loop<br>New GNRA loop<br>New GNRA loop<br>New GNRA loop	1.31/6.35	G-A tS/H or tS/W

Table S11: RNA motif subclusters belonging to GNRA motif family (continued)

Subcluster ID	Motif location	Motif family	Avg. 3D structure-based RMSD/AL	Common base-pair interactions
97	4V9F_0:1468-1474	Known GNRA loop	0.77/6.96	C-A tW/H or C-C tW/H or U-A tS/H
	6ERI_BA:566-572	New GNRA loop		
	5J7L_AA:617-623	New GNRA loop		
	6HA1_a:626-632	New GNRA loop		
	4LFB_A:617-623	New GNRA loop		
	5NGM_Aa:625-631	New GNRA loop		
	6AZ3_1:1130-1136	New GNRA loop		
	2AZX_D:532-538	New GNRA loop		
98	4V8P_D1:1886-1892	Known GNRA loop	1.13/5.49	G-A tS/H or U-A tS/H or U-C tW/H or G-U tW/H or U-A tW/H or U-G tW/H or A-U tS/H
	6CHR_A:447-453	Known GNRA loop		
	4AL5_B:10-16	Known GNRA loop		
	2XLK_C:10-16	Known GNRA loop		
	4V9F_0:1594-1600	New GNRA loop		
	1J1U_B:533-539	New GNRA loop		
	5MGP_x:32-38	New GNRA loop		
	4V9F_0:670-676	New GNRA loop		
	5AN9_N:1238-1244	Outlier		
105	3K0J_E:66-73	Known GNRA loop	1.29/7.62	U-A tS/H or U-U tW/H or U-C tW/H, A-U tW/H or G-U tW/H or G-A tS/H
	6GAW_BA:536-543	Known GNRA loop		
	6ERI_AA:1120-1127	New GNRA loop		
	5V7Q_A:1221-1228	New GNRA loop		
	4IOA_X:1103-1110	New GNRA loop		
	1HC8_C:142-149	New GNRA loop		
	6HA1_A:1138-1145	New GNRA loop		
	5O60_A:1210-1217	New GNRA loop		
	5NGM_Aa:471-478	New GNRA loop		
	5TBW_1:194-201	Outlier		
	4V8P_D1:194-201	Outlier		
	4V91_1:194-201	Outlier		
	1S03_B:22-29	Outlier		
106	4Y4O_2A:955-962	Known GNRA loop	0.94/7.43	G-A tS/H or tS/W, U-C tS/H or U-C tW/H or U-A tS/H
	4V9F_0:1916-1923	New GNRA loop		
	1MMS_C:1092-1099	New GNRA loop		
	4V9F_0:1196-1203	New GNRA loop		
	5J7L_DA:712-719	New GNRA loop		
	6HA1_A:759-766	New GNRA loop		
	5D8H_A:1202-1209	New GNRA loop		
	3J79_A:135-142	Outlier		
108	4Y4O_2A:641-647	Known GNRA loop	0.94/5.19	G-A tS/H or G-A tH/H or A-C tS/H
	4IOA_X:1797-1803	Known GNRA loop		
	5OPT_E:158-164	New GNRA loop		
	3J7Q_5:1763-1769	New GNRA loop		
	6AZ1_1:159-165	New GNRA loop		
	6AZ3_7:107-113	New GNRA loop		
	3IGI_A:135-141	New GNRA loop		
117	3Q1Q_B:77-83	Known GNRA loop	1.15/4.89	G-A tS/H or U-C tW/H or G-U tS/W or C-G tS/W or G-A tS/W
	3SIU_F:40-46	New GNRA loop		
	5XJC_F:64-70	New GNRA loop		
	5ZEB_a:1146-1152	New GNRA loop		
	5T5H_B:510-516	New GNRA loop		
	5V93_a:1157-1163	New GNRA loop		
	6FF4_6:64-70	New GNRA loop		
	2ZZN_D:32-38	Outlier		
	5XXB_1:499-502	Outlier		
119	4IOA_X:966-973	Known GNRA loop	1.06/5.14	U-A tW/H or G-A tS/H or U-U tW/H or U-C tS/H or U-A tS/H
	5L4O_A:32-38	New GNRA loop		
	6AZ3_1:129-136	New GNRA loop		
	3RG5_B:32-38	New GNRA loop		
	5T83_A:57-63	New GNRA loop		
	6GAW_BA:1489-1495	New GNRA loop		
	2ZNI_C:32-38	New GNRA loop		

Table S11: RNA motif subclusters belonging to GNRA motif family (continued)

Subcluster ID	Motif location	Motif family	Avg. 3D structure-based RMSD/AL	Common base-pair interactions
127	3J79_A:2513-2518	Known GNRA loop	0.84/4.99	G-A tS/H
	6CHR_A:87-92	Known GNRA loop		
	1Y0Q_A:21-26	Known GNRA loop		
	1Y0Q_A:101-106	Known GNRA loop		
	5G2X_A:2458-2463	New GNRA loop		
	5OOL_A:3081-3086	New GNRA loop		
	5O60_A:3077-3082	New GNRA loop		
	5XXB_1:2333-2338	New GNRA loop		
	5M3H_V:3-8	Outlier		
	5FMZ_H:3-8	Outlier		
	4IOA_X:202-207	Outlier		
	4WSB_V:3-8	Outlier		
	4WRT_V:3-8	Outlier		
144	4LFB_A:296-301	Known GNRA loop	0.75/5.67	G-A tS/H or G-G tS/H or G-G tW/h or G-A tW/H
	5J7L_AA:296-301	Known GNRA loop		
	4V5O_BA:1472-1477	Known GNRA loop		
	5T5H_B:566-571	New GNRA loop		
	5NGM_Aa:304-309	New GNRA loop		
	6D9J_5:1389-1394	New GNRA loop		
	6ERI_BA:1465-1470	New GNRA loop		
	5ZEB_a:296-301	New GNRA loop		
	5XYI_2:1309-1314	New GNRA loop		
	6HA1_a:304-309	New GNRA loop		
	5O60_A:1754-1759	Outlier		
147	3PDR_X:68-73	Known GNRA loop	0.76/4	C-A tS/H or A-a tS/W or C-A tW/W
	5MRC_A:265-270	New GNRA loop		
	5T5H_A:1250-1263	New GNRA loop		
148	5F9R_A:90-95	Known GNRA loop	0.59/5.91	G-A tS/H or tS/W or tS/S
	4ENC_A:28-33	Known GNRA loop		
	4WZJ_XXX:12-17	Known GNRA loop		
	5FQ5_A:72-77	Known GNRA loop		
	5B2T_A:72-77	Known GNRA loop		
	4ZT0_D:72-77	Known GNRA loop		
	5X2G_B:32-37	Known GNRA loop		
	5F9R_A:41-46	Known GNRA loop		
	4V8P_D1:381-386	Known GNRA loop		
	3J79_A:1693-1698	Known GNRA loop		
	5TBW_1:382-387	Known GNRA loop		
	4Y1M_B:55-60	Known GNRA loop		
	5MRC_aa:928-933	New GNRA loop		
	6ERI_AA:261-266	New GNRA loop		
	4V91_1:382-387	New GNRA loop		
	5MRC_aa:792-797	New GNRA loop		
	5VZL_B:90-95	New GNRA loop		
	4IOA_X:622-627	Outlier		
149	4V5O_BA:1056-1061	Known GNRA loop	0.54/5.88	G-A tS/H
	4V88_A6:1084-1089	Known GNRA loop		
	4ZT0_D:32-37	Known GNRA loop		
	1NBS_B:204-209	Known GNRA loop		
	5FQ5_A:32-37	Known GNRA loop		
	5FJC_A:49-54	Known GNRA loop		
	3J7A_A:1185-1190	Known GNRA loop		
	5B2T_A:32-37	Known GNRA loop		
	4Y1M_B:81-86	Known GNRA loop		
	3J7Q_5:3724-3729	New GNRA loop		
	5XYI_2:893-898	New GNRA loop		
	2YGH_A:49-54	New GNRA loop		
	4V91_1:2220-2225	New GNRA loop		
	5TBW_1:2220-2225	New GNRA loop		
	6FYY_2:1083-1088	New GNRA loop		
	6D9J_5:3724-3729	New GNRA loop		
	4Y4O_2A:225-230	Outlier		
177	3Q1Q_C:37-42	Known GNRA loop	1.35/5.67	C-C tW/H
	6GAZ_AA:160-165	New GNRA loop		
	3J9M_AA:894-899	Outlier		
182	4V9F_0:1793-1799	Known GNRA loop	1.45/4.33	G-A tS/H
	4LFB_A:458-474	New GNRA loop		
	4RUM_A:27-33	Outlier		

Table S11: RNA motif subclusters belonging to GNRA motif family (continued)

Subcluster ID	Motif location	Motif family	Avg. 3D structure-based RMSD/AL	Common base-pair interactions
205	4AOB_A:49-54<br>5MRC_A:539-546<br>4V5O_BA:1330-1336<br>5MRC_A:324-332<br>6GAZ_AV:13-19<br>6ERI_AA:1195-1202	Known GNRA loop<br>New GNRA loop<br>New GNRA loop<br>New GNRA loop<br>Outlier<br>Outlier	1.19/5.53	A-A or A-U or G-A tS/H
218	5J7L_DA:1222-1227<br>5XY3_1:1116-1121<br>3NDB_M:162-167	Known GNRA loop<br>New GNRA loop<br>New GNRA loop	0.46/4.33	G-A tS/H or G-A tW/H or G-G tW/H
224	4WF9_X:999-1006<br>5XXB_1:197-204<br>5XY3_1:172-179	Known GNRA loop<br>New GNRA loop<br>New GNRA loop	1.07/6	C-A tS/H or A-a tS/W or C-A tW/W
277	3KTW_C:163-168<br>6ERI_Ax:86-91	Known GNRA loop<br>New GNRA loop	0.79/6	G-G tW/H or tS/H

For each subcluster, the average (avg.) 3D structure-based RMSD, alignment length (AL) and common base-pairs are generated after excluding the outliers.

Table S12: RNA motif subclusters belonging to T-loop motif family

Subcluster ID	Motif location	Motif family	Avg. 3D structure-based RMSD/AL	Common base-pair interactions
2	2ZUE_B:953-961<br>2ZZN_D:53-61<br>2ZZM_B:53-61<br>1J1U_B:554-562<br>4WT8_D4:54-62<br>4YYE_C:53-61<br>4TUE_QV:53-61<br>4V9Q_DV:53-61<br>3WQY_C:1653-1661<br>5WT1_C:53-61<br>5WWT_C:53-61<br>5L4O_A:53-61<br>3J92_2:53-61<br>6CFJ_1x:53-61<br>6I0Y_V:53-61<br>5E81_2K:54-62<br>5X6B_P:52-60	Known T-loop<br>Known T-loop<br>Known T-loop<br>Known T-loop<br>Known T-loop<br>Known T-loop<br>Known T-loop<br>Known T-loop<br>Known T-loop<br>New T-loop<br>New T-loop<br>New T-loop<br>New T-loop<br>New T-loop<br>New T-loop<br>New T-loop<br>New T-loop	0.43/8.22	U-A tW/H, U-A tS/H
5	5J7L_DA:305-312<br>4WF9_X:348-355<br>4IOA_X:316-323<br>6ERI_AA:983-990<br>5V7Q_A:1083-1090<br>6HA1_A:349-356<br>5MRC_A:881-888<br>5O60_A:1072-1079<br>6ERI_AA:723-730<br>5O60_A:827-834<br>4KJI_C:5-12	Known T-loop<br>Known T-loop<br>Known T-loop<br>New T-loop<br>New T-loop<br>New T-loop<br>New T-loop<br>New T-loop<br>Outlier<br>Outlier<br>Outlier	1.18/6.15	U-A tW/H or G-A tS/H
15	1U0B_A:53-61<br>1B23_R:53-61<br>2D6F_F:953-961<br>2DLC_Y:553-561<br>1QF6_B:53-61<br>4V8Q_BY:66-74<br>2CZJ_H:53-61<br>5LZD_y:53-61<br>5T5H_B:1393-1437	Known T-loop<br>Known T-loop<br>Known T-loop<br>Known T-loop<br>Known T-loop<br>Known T-loop<br>Known T-loop<br>New T-loop<br>Outlier	0.88/6.44	U-A tW/H

Table S12: RNA motif subclusters belonging to T-loop motif family (continued)

Subcluster ID	Motif location	Motif family	Avg. 3D structure-based RMSD/AL	Common base-pair interactions
	3EPH_E:53-61	Known T-loop		
	5AXM_P:53-61	Known T-loop		
	3VJR_B:13-21	Known T-loop		
	3ICQ_E:53-61	Known T-loop		
	1EVV_A:53-61	New T-loop		
	5IBB_1K:53-61	New T-loop		
16	4V4J_2:53-61	New T-loop	0.99/7.31	U-A tW/H
	6AH3_T:53-61	New T-loop		
	6GZ4_Bw:53-61	New T-loop		
	5IBB_1L:53-61	New T-loop		
	1ZL3_B:408-416	New T-loop		
	1R3E_C:408-416	New T-loop		
	5JU8_AY:53-61	New T-loop		
	1H3E_B:53-61	Known T-loop		
	2DU3_D:952-960	Known T-loop		
	1WZ2_D:965-973	Known T-loop		
	4LCK_B:52-60	Known T-loop		
	1QTQ_B:953-961	Known T-loop		
	3AMU_B:53-61	Known T-loop		
	2AKE_B:53-61	Known T-loop		
	2AZX_D:553-561	Known T-loop		
	2DER_D:53-61	Known T-loop		
	1SER_T:53-61	Known T-loop		
31	5HR7_D:52-60	Known T-loop	0.93/6.64	U-A tW/H, U-A tS/H
	4V9K_AW:53-61	Known T-loop		
	5HR6_C:52-60	Known T-loop		
	4JXZ_B:953-961	Known T-loop		
	4V8N_CV:55-63	Known T-loop		
	5MRC_bb:53-61	New T-loop		
	6GSL_3L:54-62	New T-loop		
	5VPP_XV:55-63	New T-loop		
	5IB8_1L:53-61	New T-loop		
	4V5G_AY:53-61	New T-loop		
	5E81_3L:53-61	New T-loop		
	5VPP_QV:55-63	New T-loop		
	5E81_1K:53-61	New T-loop		
	6GSL_1K:54-62	New T-loop		
	6GSL_1L:54-62	New T-loop		
	5MGP_x:53-61	New T-loop		
	5B63_D:954-962	New T-loop		
	1H4S_T:53-61	New T-loop		
	6HD7_A:53-61	New T-loop		
	6D9J_5:4417-4425	Outlier		
	5XXB_1:2835-2843	Outlier		
	3J7Q_5:4301-4309	Outlier		
	3J7Q_5:4417-4425	Outlier		
	5OPT_E:818-826	Outlier		
	5XXB_1:2951-2959	Outlier		
	5T2A_B:1214-1222	Outlier		
	4ATO_G:16-24	Outlier		
	6AZ3_2:1279-1287	Outlier		
	5TBW_1:2840-2848	Outlier		
	4V91_1:2840-2848	Outlier		
	1F7U_B:953-961	Known T-loop		
	1J2B_C:954-962	Known T-loop		
	2BTE_B:53-61	Known T-loop		
	3OVB_C:12-20	Known T-loop		
	2NR0_E:53-61	Known T-loop		
	1FIR_A:53-61	Known T-loop		
	1GAX_D:952-960	Known T-loop		
	4RDX_C:53-61	Known T-loop		
	1QU2_T:53-61	Known T-loop		
32	5AH5_D:53-61	Known T-loop	0.68/8.17	U-A tW/H, U-A tS/H
	5LZS_3:53-61	New T-loop		
	6HA8_x:53-61	New T-loop		
	6D9J_3:64-72	New T-loop		
	6D90_3:64-72	New T-loop		
	6HA1_x:64-72	New T-loop		
	4WSM_3L:64-72	New T-loop		
	5O2R_x:53-61	New T-loop		
	6MTC_4:53-61	New T-loop		
	5CCB_N:53-61	Outlier		

Table S12: RNA motif subclusters belonging to T-loop motif family (continued)

Subcluster ID	Motif location	Motif family	Avg. 3D structure-based RMSD/AL	Common base-pair interactions
51	5J7L_AA:321-332<br>4V88_A6:393-404<br>4V5O_BA:384-395<br>6HA1_a:329-340<br>6ERI_BA:293-304<br>3J7P_S2:441-452<br>5NGM_Aa:329-340<br>5XXU_2:393-404<br>6D9J_2:441-452<br>4LFB_A:321-332<br>6FYY_2:392-403<br>2ZUE_B:913-922	Known T-loop<br>Known T-loop<br>Known T-loop<br>New T-loop<br>New T-loop<br>New T-loop<br>New T-loop<br>New T-loop<br>New T-loop<br>New T-loop<br>New T-loop<br>Outlier	0.94/11.12	U-A tW/H, C-A tW/H, G-G tS/H, C-C tH/W or tH/H
60	4YCO_D:53-61<br>3Q1Q_C:63-71<br>2DRA_B:12-20<br>3W3S_B:53-61<br>3WFS_A:53-61<br>3AM1_B:59-67<br>4V90_AV:53-61<br>3TUP_T:53-61<br>5E6M_C:53-61<br>4QEI_C:53-61<br>1N78_C:553-561<br>5MMM_z:53-61<br>3J7R_S5:53-61<br>5TCU_D:53-61<br>1P6V_B:45-53<br>5IMQ_5:53-61<br>5XYI_2:260-268<br>6HCJ_q3:53-61<br>5AJ0_Bv:53-61	Known T-loop<br>Known T-loop<br>Known T-loop<br>Known T-loop<br>Known T-loop<br>Known T-loop<br>Known T-loop<br>Known T-loop<br>Known T-loop<br>Known T-loop<br>Known T-loop<br>New T-loop<br>New T-loop<br>New T-loop<br>New T-loop<br>New T-loop<br>New T-loop<br>New T-loop<br>New T-loop	0.85/8.9	U-A tW/H, U-A tS/H
72	2CSX_C:53-61<br>3AL0_E:53-61<br>3A2K_C:54-62<br>1C0A_B:653-661<br>4MGN_B:52-60<br>3KFU_L:53-61<br>4WJ4_B:53-61<br>4X4V_B:12-20<br>4WJ3_S:53-61<br>4YCP_B:53-61<br>6HD7_B:53-61<br>3J9W_AX:53-61<br>5JTE_AX:53-61<br>5T5H_G:42-60	Known T-loop<br>Known T-loop<br>Known T-loop<br>Known T-loop<br>Known T-loop<br>Known T-loop<br>Known T-loop<br>Known T-loop<br>Known T-loop<br>Known T-loop<br>New T-loop<br>New T-loop<br>New T-loop<br>Outlier	0.62/7.67	U-A tW/H
89	4Y4O_2A:1281-1289<br>4GMA_Z:58-66<br>6CHR_A:31-39<br>4WF9_X:1318-1326<br>4IOA_X:1294-1302<br>6ERI_AA:1302-1310<br>5TBW_1:216-224<br>6HA1_A:1320-1328<br>4V91_1:216-224<br>6ERI_AA:336-344<br>5XXB_1:219-227<br>2AKE_B:13-22<br>3SKL_B:38-46<br>3AL0_E:13-22<br>2AZX_D:513-522<br>3SKI_A:39-47<br>6CU1_A:58-66<br>6ERI_BA:231-239	Known T-loop<br>Known T-loop<br>Known T-loop<br>Known T-loop<br>Known T-loop<br>New T-loop<br>New T-loop<br>New T-loop<br>New T-loop<br>New T-loop<br>New T-loop<br>Outlier<br>Outlier<br>Outlier<br>Outlier<br>Outlier<br>Outlier<br>Outlier	0.94/6.45	U-A tW/H
91	5C45_X:17-25<br>4FRG_B:28-36<br>6DN2_X:17-25<br>6DN2_Y:68-76<br>3F2X_X:17-25<br>3F2X_X:68-76<br>5C45_Y:68-76<br>4V88_A6:764-772	Known T-loop<br>Known T-loop<br>Known T-loop<br>Known T-loop<br>Known T-loop<br>Known T-loop<br>Known T-loop<br>New T-loop	1.03/7	U-A tW/H or C-G tW/W

Table S12: RNA motif subclusters belonging to T-loop motif family (continued)

Subcluster ID	Motif location	Motif family	Avg. 3D structure-based RMSD/AL	Common base-pair interactions
91	6FYY_2:764-772<br>3J7A_A:814-822<br>5TBW_1:2442-2505	New T-loop<br>New T-loop<br>New T-loop		
140	4JRC_B:51-63<br>4MGN_A:51-63	Known T-loop<br>Known T-loop	0.38/13	U-A tW/H
155	3ADD_C:53-61<br>3RG5_B:53-61<br>4FRN_B:56-64<br>4FRG_B:58-66<br>4ZDO_E:53-61<br>1IL2_C:953-961<br>6GAW_BA:1294-1302<br>5AN9_N:3057-3065	Known T-loop<br>Known T-loop<br>New T-loop<br>New T-loop<br>New T-loop<br>New T-loop<br>Outlier<br>Outlier	1.37/7.36	U-A tW/H
258	4GXY_A:49-57<br>5XYI_2:612-620<br>5Y85_D:11-19<br>6CU1_A:24-32<br>5J7L_DA:2304-2312<br>5LZS_3:13-22	Known T-loop<br>New T-loop<br>New T-loop<br>New T-loop<br>Outlier<br>Outlier	1.09/5.93	U-A or C-G tW/H
265	4GCW_B:53-61<br>3JBV_W:31-39	Known T-loop<br>New T-loop	1.82/8	U-A or U-C tW/H
279	4FRN_B:28-36<br>5UD5_C:53-61<br>5XXU_2:761-769<br>5XYI_2:1203-1211	Known T-loop<br>New T-loop<br>New T-loop<br>Outlier	1.01/6.0	U-A tW/H
308	4V9F_0:623-632<br>6HIX_AA:406-414<br>5MRC_A:1849-1857	Known T-loop<br>New T-loop<br>New T-loop	2.09/7.67	U-A tW/H or U-A tS/H or G-A tS/H
320	4V5O_BA:1522-1533	Known T-loop	0/12	U-A tW/H
412	4Y4O_2A:305-312	Known T-loop	0/8	U-A tW/H

For each subcluster, the average (avg.) 3D structure-based RMSD, alignment length (AL) and common base-pairs are generated after excluding the outliers.

Table S13: Base interaction variations within Sarcin-ricin subclusters

Subcluster id	16	27	29	34
Base interaction	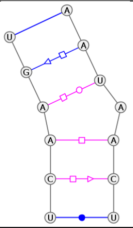	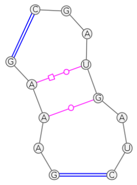	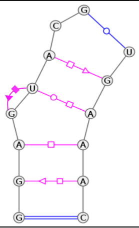	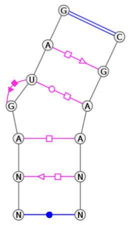
Total motifs	3	26	13	11
Known SR/New SR/Outliers	1/1/1	3/19/4	3/6/4	4/3/4
Avg. BP-based RMSD/AL	0.22/13	2.54/9.72	1.93/8.4	2.6/6.8
Avg. SC-based RMSD/AL	0.23/13	1.49/10.14	1.2/10.4	1.5/8.4
Common features	A-G tH/S,<br>U-A tW/H,<br>A-bulge,<br>A-A tH/H<br>C-C tS/H	A-G tW/W<br>or tH/W,<br>A-U tH/W	A-G tH/S or tW/S,<br>U-A tW/H,<br>G bulge, G-U cS/H,<br>A-A tH/H,<br>tS/H or tS/W	A-G tH/S,<br>U-A tW/H,<br>G-bulge, G-U cS/H,<br>A-A tH/H<br>tS/H or tW/H

Table S13: Base interaction variations within Sarcin-ricin subclusters (continued)

Subcluster id	63	70	89	95
Base interaction	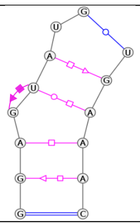	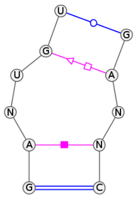	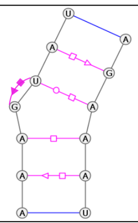	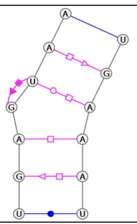
Total motifs	11	8	3	8
Known SR/New SR/Outliers	2/8/1	1/5/2	3/0/0	3/3/2
Avg. BP-based RMSD/AL	2.1/8.5	2.20/6.17	0.47/11.7	2.3/8.7
Avg. SC-based RMSD/AL	1.3/10.5	1.21/7.71	0.53/13.0	0.99/11.4
Common features	A-G tH/S,<br>U-A tW/H,<br>G-bulge,<br>G-U cS/H,<br>A-A tH/H,<br>C-C tS/H	A-U cH/H<br>or tH/H,<br>G-A tS/H	A-G or A-A tH/S,<br>U-A tW/H,<br>G bulge,<br>G-U cS/H,<br>A-A tH/H,<br>A-A or G-A tS/H	A-G tH/S,<br>U-A tW/H,<br>G-bulge, G-U cS/H,<br>A-A tH/H or tW/H,<br>G-A or U-A tS/H
Subcluster id	122	134	151	167
Base interaction	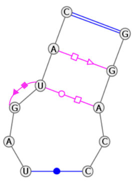	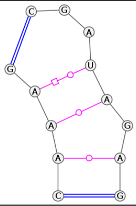	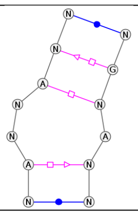	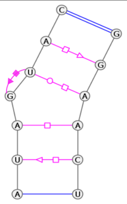
Total motifs	11	14	3	8
Known SR/New SR/Outliers	4/6/1	5/8/1	1/1/1	4/4/0
Avg. BP-based RMSD/AL	1.5/8.2	2.29/10.17	1.46/10.5	0.6/10.8
Avg. SC-based RMSD/AL	0.7/8.9	1.50/11.15	1.44/13	0.78/13
Common features	A-G tH/S,<br>U-A tW/H,<br>G-bulge,<br>G-U cS/H	A-A tW/W or tH/S,<br>A-A tW/W or tH/W,<br>A-U tH/W or tH/S	A-G tH/S,<br>A-A tH/H,<br>tS/H	A-G tH/S,<br>U-A tW/H, G bulge,<br>G-U cS/H, A-A tH/H,<br>U-C or A-C tS/H,
Subcluster id	191	205	247	249
Base interaction	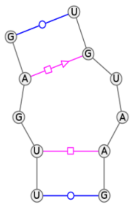	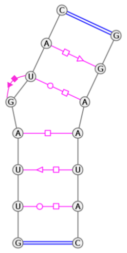	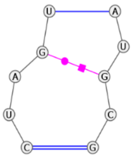	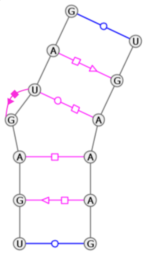
Total motifs	3	6	3	10
Known SR/New SR/Outliers	2/1/0	2/4/0	2/0/1	7/2/1
Avg. BP-based RMSD/AL	0.26/6.3	0.58/12.3	0.48/10	2.35/7.49
Avg. SC-based RMSD/AL	0.47/11	0.76/15	0.58/10	1.58/11.19
Common features	A-G tH/S,<br>G bulge,<br>U-A cH/H<br>or tH/H	A-G tH/S, U-A tW/H,<br>G bulge, G-U cS/H,<br>A-A tH/H,<br>U-U or U-G tS/H,<br>U-A or U-C tW/H	G-G cW/H	A-G tH/S,<br>U-A tW/H, G-bulge,<br>G-U cS/H or U-A cW/H,<br>A-A tH/H,<br>G-A tS/H

Table S13: Base interaction variations within Sarcin-ricin subclusters (continued)

Subcluster id	252	277	293	313
Base interaction	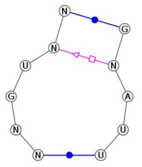	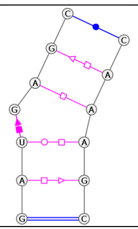	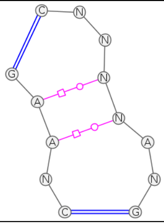	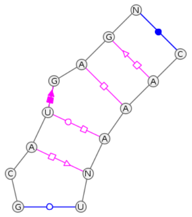
Total motifs	11	5	8	6
Known SR/New SR/Outliers	1/1/1	1/2/2	1/2/0	1/3/2
Avg. BP-based RMSD/AL	3.18/4	3.96/10.33	2.54/5.33	0.47/12.5
Avg. SC-based RMSD/AL	1.88/8	1.59/12.67	2.24/7.33	0.50/14.0
Common features	A-G tH/S	A-G tH/S,<br>U-A or A-A tW/H,<br>G-bulge,<br>G-U cS/H	U-A or A-A tW/H,<br>A-A tH/H	A-A or A-G tH/S,<br>U-A tW/H,<br>G bulge, G-U cS/H,<br>A-A tH/H, G-A tS/H
Subcluster id	358	362	392	
Base interaction	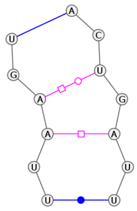	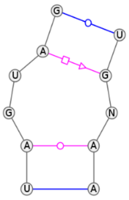	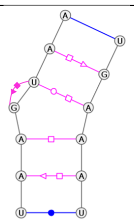	
Total motifs	3	14	8	
Known SR/New SR/Outliers	1/1/1	5/8/1	4/4/0	
Avg. BP-based RMSD/AL	2.24/11.5	2.29/10.17	0.6/10.8	
Avg. SC-based RMSD/AL	0.87/15.0	1.50/11.15	0.78/13	
Common features	A-A tH/H,<br>U-A tW/H	A-A tW/W or tH/S,<br>A-A tW/W or tH/W,<br>A-U tH/W or tH/S	A-G tH/S,<br>U-A tW/H, G bulge,<br>G-U cS/H, A-A tH/H,<br>U-C or A-C tS/H,	

For each subcluster, the average (avg.) base interaction-based RMSD along with alignment length (AL), 3D structure-based RMSD along with alignment length and common features are generated after excluding the outliers.

Table S14: RNA motif subclusters belonging to Tetraloop-receptor motif family

Subcluster ID	Motif location	Motif family	Avg. 3D structure-based RMSD/AL	Common base-pair interactions
61	1KXK_A:15-19_54-57<br>1U6B_B:61-65_80-83<br>2R8S_R:223-227_247-250<br>3Q1Q_C:32-35_44-48<br>4K27_U:19-23_34-37<br>4WZJ_XXX:46-49_56-60<br>5BTM_A:19-23_34-37<br>5M0H_A:14-18_29-32<br>6CHR_A:518-521_532-536<br>5Y7M_D:3-6_46-50<br>6AZ4_A:13-17_28-31<br>5XTM_B:3-6_41-45<br>3JB9_C:13-14_78-84<br>5J7L_DA:275-280_360-362<br>6HA1_A:891-895_977-980<br>6AZ1_1:1788-1790_1808-1813<br>5TC1_R:2783-2787_2791-2794	Known Tetraloop-receptor<br>Known Tetraloop-receptor<br>Known Tetraloop-receptor<br>Known Tetraloop-receptor<br>Known Tetraloop-receptor<br>Known Tetraloop-receptor<br>Known Tetraloop-receptor<br>Known Tetraloop-receptor<br>Known Tetraloop-receptor<br>New Tetraloop-receptor<br>New Tetraloop-receptor<br>New Tetraloop-receptor<br>Outlier<br>Outlier<br>Outlier<br>Outlier<br>Outlier	0.66/9.0	A-A cS/H,<br>U-A tW/H

For each subcluster, the average (avg.) 3D structure-based RMSD, alignment length (AL) and common base-pairs are generated after excluding the outliers.

Table S15: RNA motif subclusters belonging to reverse Kink-turn motif family

Subcluster ID	Motif location	Motif family	Avg. 3D structure-based RMSD/AL	Common base-pair interactions
18	4V8P_D1:1222-1232_1326-1340<br>5TBW_1:1195-1205_1299-1313<br>4V91_1:1195-1205_1299-1313<br>6AZ3_3:28-36_187-203<br>5AN9_N:1430-1440_1534-1548<br>5XY3_1:918-928_1022-1036<br>4N0T_B:39-59_88-92<br>5AN9_N:2472-2482_2495-2503<br>6GAZ_AA:306-321_389-396	Known reverse Kink-turn<br>Known reverse Kink-turn<br>New reverse Kink-turn<br>New reverse Kink-turn<br>New reverse Kink-turn<br>New reverse Kink-turn<br>Outlier<br>Outlier<br>Outlier	1.29/21.73	A-G or A-A tH/S,<br>A-A tH/H,<br>G-G cS/H,<br>U-C cH/H
26	4Y4O_2A:1024-1030_1124-1140<br>6HA1_A:1070-1076_1170-1186<br>4WF9_X:1068-1074_1168-1184<br>6ERI_AA:1052-1058_1152-1167<br>3J7Q_5:4239-4243_4267-4285	Known reverse Kink-turn<br>New reverse Kink-turn<br>New reverse Kink-turn<br>New reverse Kink-turn<br>Outlier	1.18/23.17	A-G tH/S,<br>A-A tH/H,<br>G-U cS/H or G-A cS/W,<br>C-G tW/H
195	3J7Q_5:1934-1944_2038-2052<br>6EK0_L5:1934-1944_2038-2052<br>6D9J_5:1934-1944_2038-2052<br>5G2X_A:2447-2456_2465-2475	Known reverse Kink-turn<br>New reverse Kink-turn<br>New reverse Kink-turn<br>Outlier	0.99/25.33	A-G tH/S,<br>A-A tH/H,<br>G-G cS/H,<br>U-C cH/H
246	5J7L_DA:1024-1030_1124-1140<br>5O60_A:1142-1148_1242-1258<br>5V7Q_A:1153-1159_1253-1269<br>3J7Q_5:4755-4765_4869-4878	Known reverse Kink-turn<br>New reverse Kink-turn<br>New reverse Kink-turn<br>Outlier	0.76/24.0	A-G tH/S,<br>A-A tH/H,<br>G-U cS/H,<br>C-G tW/H
284	3J79_A:1323-1333_1427-1441<br>5XXB_1:1282-1292_1386-1400<br>6GAW_BA:4-8_132-147	Known reverse Kink-turn<br>New reverse Kink-turn<br>Outlier	0.56/26.0	A-G tH/S,<br>A-A tH/H, G-G cS/H,<br>U-C cH/H
373	5MRC_A:950-957_1091-1105<br>3JCS_3:28-33_192-203<br>5MRC_aa:1278-1287_1324-1331	Known reverse Kink-turn<br>New reverse Kink-turn<br>Outlier	2.39/12.0	A-G or A-A tH/S,<br>A-A tH/H

For each subcluster, the average (avg.) 3D structure-based RMSD, alignment length (AL) and common base-pairs are generated after excluding the outliers.

Table S16: RNA motif subclusters belonging to L1-complex motif family

Subcluster ID	Motif location	Motif family	Avg. 3D structure-based RMSD/AL	Common base-pair interactions
92	1U63_D:10-13_33-40<br>2VPL_B:10-13_33-40<br>4V8P_D1:457-462_516-521	Known L1-complex<br>Known L1-complex<br>Outlier	0.41/12.0	A-G tS/S,<br>G-A tH/S
164	1MZP_B:20-23_32-45<br>3U4M_B:2124-2127_2161-2174<br>4Y4O_2A:2124-2127_2161-2174<br>4V91_1:2466-2469_2476-2489	Known L1-complex<br>Known L1-complex<br>New L1-complex<br>New L1-complex	1.16/18.0	G-A tS/H,<br>G-A tS/S,<br>U-A tW/H,<br>G-U tW/H

For each subcluster, the average (avg.) 3D structure-based RMSD, alignment length (AL) and common base-pairs are generated after excluding the outliers.

Table S17: RNA motif subclusters belonging to Rope-sling motif family

Subcluster ID	Motif location	Motif family	Avg. 3D structure-based RMSD/AL	Common base-pair interactions
241	4LFB_A:504-511_540-541<br>5V93_a:495-502_531-532<br>5ZEB_a:484-491_520-521<br>4V9F_0:705-709_719-723<br>5XXB_1:809-812_832-836	Known Rope-sling<br>New Rope-sling<br>New Rope-sling<br>Outlier<br>Outlier	0.79/10.0	C-A cS/H or cS/W
256	4Y4O_2A:1004-1011_1150-1151<br>5V7Q_A:1133-1140_1280-1281	Known Rope-sling<br>New Rope-sling	0.32/10.0	C-A cS/H
309	5J7L_DA:1004-1011_1150-1151<br>6HA1_A:1050-1057_1196-1197	Known Rope-sling<br>New Rope-sling	0.39/10.0	A-A cS/H

For each subcluster, the average (avg.) 3D structure-based RMSD, alignment length (AL) and common base-pairs are generated after excluding the outliers.

Table S18: List of top 10 RNA motif subclusters (excluding pre-defined subclusters) based on base interaction similarity

Subcluster ID	Motif location	Motif family	Avg. 3D structure-based RMSD/AL	Common base-pair interactions
269	5J7L_DA:481-484_496-509<br>4IOA_X:492-495_506-519	New Double-kink-turn<br>New Double-kink-turn	0.56/18.0	A-A tH/S, A-A cS/W, A-A tH/H, G-G tS/S,<br>G-G tW/H, U-A tW/H, G-A tS/H
53	6ERI_AA:493-496_507-520<br>4V91_1:376-379_390-403<br>5TBW_1:376-379_390-403	New Double-kink-turn<br>New Double-kink-turn<br>New Double-kink-turn	0.88/18.0	A-A tH/S, A-A cS/W, A-A tH/H,<br>G-G tS/S, G-G tW/H,<br>U-A tW/H, G-A tS/H
288	6CB3_B:7-14_91-96<br>4Y1J_A:7-14_91-96	New Sarcin-ricin<br>New Sarcin-ricin	0.67/14.0	A-A tH/S, U-A tW/H, G-U cS/H,<br>G-G tW/H, G-A tS/H
226	4Y4O_2A:481-484_496-509<br>4V9F_0:487-490_502-515<br>6HA1_A:528-531_542-555	New Double-kink-turn<br>New Double-kink-turn<br>New Double-kink-turn	1.03/18.0	A-A or A-G tH/S, A-A or C-A cS/W,<br>A-A tH/H, G-G tS/S,<br>G-G tW/H, U-A tW/H, G-A tS/H
294	5O60_A:1378-1384_2235-2240<br>5V7Q_A:1394-1400_2249-2254	New Sarcin-ricin<br>New Sarcin-ricin	0.43/13.0	A-G tH/S, U-A tW/H, G-U cS/H,<br>A-A tH/H, G-A tS/H
238	6ERI_BA:464-470_477-485<br>5MRC_aa:629-635_642-650	New Kink-turn<br>New Kink-turn	0.5/16.0	A-G tH/S, A-C tW/S,<br>C-U cW/S, U-A tW/H
184	5OOL_A:3120-3126_3131-3136<br>6EK0_L5:4597-4603_4608-4613<br>3J7Q_5:4597-4603_4608-4613	New Sarcin-ricin<br>New Sarcin-ricin<br>New Sarcin-ricin	0.72/12.33	A-G tH/S, U-A tW/H,<br>G-U cS/H, A-A tH/H,<br>C-C tS/H
321	5T5H_A:1612-1620_1638-1644<br>6AZ3_1:1479-1487_1505-1511	New Kink-turn<br>New Kink-turn	0.32/16.0	A-G tH/S, A-G tH/S, G-A tS/H,<br>A-G cW/S, G-G tS/S
377	4V91_1:517-525_567-573<br>5TBW_1:517-525_567-573	New 3-point-turn<br>New 3-point-turn	0.63/16.0	A-A tH/W, A-A tH/W,<br>A-U tH/W, G-A cH/W
174	4KQY_A:17-21_31-38<br>5XY3_1:1385-1391_1401-1407	New Sarcin-ricin<br>New Sarcin-ricin	0.37/13.0	A-G tH/S, U-A tW/H, G-U cS/H,<br>or cS/W, A-A tH/H, G-A tS/H

For each subcluster, the average (avg.) 3D structure-based RMSD, alignment length (AL) and common base-pairs are generated after excluding the outliers.

Table S19: List of top 10 RNA motif subclusters (excluding pre-defined subclusters) based on 3D structure similarity

Subcluster ID	Motif location	Motif family	Avg. 3D structure-based RMSD/AL	Common base-pair interactions
11	5T2A_B:81-84_519-520<br>6EK0_L5:2847-2850_3840-3841<br>5XXB_1:2001-2004_2449-2450<br>3J7Q_5:2847-2850_3840-3841<br>5V7Q_A:1901-1904_2231-2232<br>4V9F_0:1744-1747_2034-2035<br>5MRC_A:1618-1621_1893-1894<br>5OOL_A:2470-2473_2656-2657<br>5O60_A:1884-1887_2217-2218<br>4WF9_X:1710-1713_2020-2021<br>6AZ3_2:82-85_582-583<br>6GAW_BA:801-804_988-989<br>6ERI_AA:1702-1705_2007-2008<br>5XY3_1:1563-1566_1854-1855<br>5J7L_DA:1666-1669_1993-1994<br>4Y4O_2A:1666-1669_1993-1994<br>4V8P_D1:1922-1925_2331-2332<br>4IOA_X:1683-1686_1976-1977<br>4V91_1:1898-1901_2336-2337<br>5T5H_B:82-85_682-683<br>6HA1_A:1711-1714_2022-2023<br>5TBW_1:1898-1901_2336-2337<br>3J79_A:2166-2169_2629-2630	New Anchor<br>New Anchor	0.23/5.83	G-A cS/H,<br>G-U tS/H

Table S19: List of top 10 RNA motif subclusters (excluding pre-defined subclusters) based on 3D structure similarity (continued)

Subcluster ID	Motif location	Motif family	Avg. 3D structure-based RMSD/AL	Common base-pair interactions
20	5XXU_2:50-52_428-429	New Bow-loop	0.28/5.0	A-G tH/S
	3J7A_A:50-52_434-435	New Bow-loop		
	4LFB_A:54-56_356-357	New Bow-loop		
	5XYI_2:48-50_359-360	New Bow-loop		
	6D9J_2:49-51_476-477	New Bow-loop		
	5J7L_AA:54-56_356-357	New Bow-loop		
	3J9M_AA:694-696_819-820	New Bow-loop		
	6AZ1_1:50-52_471-472	New Bow-loop		
	5OPT_E:50-52_475-476	New Bow-loop		
	6FYY_2:50-52_427-428	New Bow-loop		
	6GAZ_AA:48-50_175-176	New Bow-loop		
	6HA1_a:56-58_364-365	New Bow-loop		
	6EK0_S2:49-51_476-477	New Bow-loop		
	6ERI_BA:56-58_328-329	New Bow-loop		
	5MRC_aa:61-63_360-361	New Bow-loop		
	5V93_a:57-59_355-356	New Bow-loop		
	4V88_A6:50-52_428-429	New Bow-loop		
	4V5O_BA:49-51_420-421	New Bow-loop		
	5ZEB_a:58-60_356-357	New Bow-loop		
45	3J7Q_5:1737-1738_1790-1792	New Parabolic-loop	0.46/5.0	A-U tS/H, U-U cS/H
	6D9J_5:1737-1738_1790-1792	New Parabolic-loop		
	6EK0_L5:1737-1738_1790-1792	New Parabolic-loop		
	6AZ3_1:1048-1049_1101-1103	New Parabolic-loop		
	4V8P_D1:1023-1024_1076-1078	New Parabolic-loop		
	4V91_1:997-998_1050-1052	New Parabolic-loop		
	5TBW_1:997-998_1050-1052	New Parabolic-loop		
	5XXB_1:1086-1087_1139-1141	New Parabolic-loop		
323	4WF9_X:1695-1700_2031-2033	New Tau-loop	0.34/9.0	A-A or A-G tH/S, cW/H
	6ERI_AA:1687-1692_2018-2020	New Tau-loop		
157	3J7Q_5:3927-3929_4181-4184	New Beta-loop	0.38/7.0	A-G tH/S
	5J7L_DA:2081-2083_2236-2239	New Beta-loop		
	6D9J_5:3927-3929_4181-4184	New Beta-loop		
	5AN9_N:2689-2691_2937-2940	New Beta-loop		
	4V91_1:2423-2425_2604-2607	New Beta-loop		
	6EK0_L5:3927-3929_4181-4184	New Beta-loop		
	5TBW_1:2423-2425_2604-2607	New Beta-loop		
	3J79_A:2716-2718_2943-2946	New Beta-loop		
393	6AZ3_2:402-407_419-426	New Double-handle loop	0.4/14.0	G-U tW/W, A-U tW/W, G-A tS/S
	5T2A_B:339-344_356-363	New Double-handle loop		
198	6ERI_AA:2474-2476_2510-2511	New Parabolic-loop	0.26/3.87	G-A cS/H
	5O60_A:2681-2683_2717-2718	New Parabolic-loop		
	4Y4O_2A:2457-2459_2493-2494	New Parabolic-loop		
	4IOA_X:2436-2438_2472-2473	New Parabolic-loop		
	5J7L_DA:2457-2459_2493-2494	New Parabolic-loop		
	5O60_A:1312-1313_1362-1364	Outlier		
243	4Y4O_2A:738-740_757-758	New Cross-loop	0.29/4.29	G-U cS/H
	5OOL_A:1948-1950_1967-1968	New Cross-loop		
	4IOA_X:751-753_770-771	New Cross-loop		
	5MRC_A:629-631_648-649	New Cross-loop		
	4V91_1:869-871_889-890	New Cross-loop		
	3J7Q_5:1586-1588_1606-1607	New Cross-loop		
	6EK0_L5:1587-1589_1605-1606	Outlier		
143	5MRC_A:1834-1845_1861-1864	New Pi-loop	0.44/16.0	A-U cH/W, A-G tW/S
	6HA1_A:1963-1974_1990-1993	New Pi-loop		
	4WF9_X:1961-1972_1988-1991	New Pi-loop		
	6ERI_AA:1948-1959_1975-1978	New Pi-loop		
299	5T2A_B:703-709_945-949	New Sine-loop	0.44/12.0	U-A cW/S
	6AZ3_2:766-772_1010-1014	New Sine-loop		
	5T5H_B:866-872_1141-1145	New Sine-loop		

For each subcluster, the average (avg.) 3D structure-based RMSD, alignment length (AL) and common base-pairs are generated after excluding the outliers.
